# De Novo Design of Multivalent α/β‐Peptides Mimicking Transcription Factors Targeting the CBP KIX Domain

**DOI:** 10.1002/chem.71218

**Published:** 2026-06-02

**Authors:** Márk V. Tresztián, Vencel L. Petrovicz, Edit Wéber, Tamás A. Martinek, Zsófia Hegedüs

**Affiliations:** ^1^ Department of Medical Chemistry University of Szeged Szeged Hungary; ^2^ Biomimetic Systems Research Group HUN‐REN SZTE Szeged Hungary

**Keywords:** α/β‐peptides, peptidomimetics, transcription factors, dynamic covalent chemistry

## Abstract

Protein‐protein interactions that regulate gene expression in the nucleus are increasingly recognized as potential therapeutic targets but present unique challenges. Effective targeting requires molecules capable of interacting with large protein surfaces while remaining small enough to pass through the nuclear pore, for which peptidomimetics are promising candidates. *De novo* design strategies often focus on projecting hot‐spot residues using well‐defined, rigid scaffolds; however, other critical properties—such as solubility, charge, or local flexibility—are often overlooked. Our approach employs α/β‐peptidomimetics to enable fine‐tuning of these properties by generating manageable‐sized soluble libraries. Using the KIX domain of the coactivator proteins p300/CBP as a model system, we demonstrate that our strategy allows modular optimization of hot‐spot, solvent‐exposed, and structure‐inducing residues, thereby tuning affinity and binding‐site selectivity. Coactivator proteins often use multivalency to enhance affinity and selectivity, which we exploit by creating dimers from our initial hits. We show that both static libraries and template‐directed dynamic covalent chemistry facilitate the screening of multivalent ligands that closely mimic the native interaction partners. Our design strategy, built on α/β‐peptide building blocks, represents a promising approach to develop de novo ligands against proteins characterized by high plasticity and multivalent interactions.

## Graphical abstract

A modular, *de novo* α/β‐peptide design strategy allows fine‐tuning of ligand properties, resulting in multivalent ligands that simultaneously target separate binding sites on the protein surface.

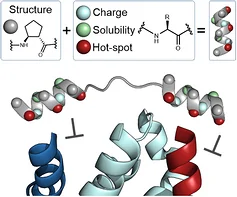

## 1 Introduction

Cells respond to different stimuli by tuning gene expression modulated by protein‐protein interactions (PPIs), such as those between transcription factors (TFs) and coactivators [1]. Malfunction of these pathways can lead to disease, making these PPIs attractive therapeutic targets [2, 3]. A major obstacle for designing ligands is the nuclear localisation of these targets, which is further complicated by the high prevalence of intrinsically disordered regions (IDRs), [4, 5, 6] where induced folding [7, 8], multivalency [9] and cooperativity [10, 11, 12] play significant roles. Small molecules often fail to achieve high‐affinity binding or disrupt interactions with complex molecular mechanisms, while the use of large therapeutic biomolecules is limited due to the size restriction of the nuclear pore complex.

Peptidomimetic structures that can reproduce protein‐like binding at a smaller molecular weight could overcome these limitations [13, 14, 15]. Top‐down design of peptidomimetics, based on native sequences, often provides selective and high‐affinity ligands [16, 17, 18, 19], but may not overcome inherent limitations of the native ligand. In vitro expression systems are promising alternatives to include non‐natural amino acids, but the use of multiple monomers with artificial backbones is still limited within a sequence [20]. On the other hand, *de novo* design and library screening strategies could offer the expansion of chemical space and create ligands by combining local surface mimetics [21], similar to a fragment‐based approach [22, 23]. *De novo* designs rely on artificial scaffolds projecting proteinogenic side‐chains [13] and are often stabilized by hydrophobic residues [24, 25, 26, 27], leading to poor solubility and aggregation. Using such well‐folded, rigid structures also limits the optimisation of the solvent‐exposed surface, charge, or local flexibility, leading to the loss of potentially important interactions. A strategy addressing these limitations could provide more efficient ligands.

In our approach, we move beyond conventional design approaches that focus solely on hot‐spot residues projected from rigid scaffolds and include optimisation steps for solvent‐exposed and structure‐inducing residues. To achieve this, we keep the number of structure‐inducing hydrophobic amino acids to a minimum and generate mixed α/β‐peptide sequences. So far, such sequences have been developed exclusively based on native sequence modification [28, 29, 30], but their potential in a *de novo* design setting has not been investigated. We hypothesise that using such biomimetics could be advantageous for targeting PPIs involving IDRs, because the less rigid ligand would allow adaptation to the protein surface, whereas the close mimicry of the overall amino acid content could recreate important transient electrostatic or cooperative interactions.

**FIGURE 1:**
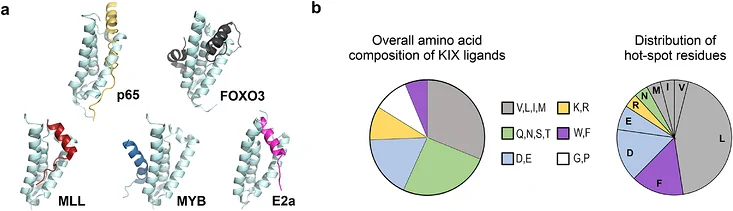
(a) Structure of KIX bound to different transcription factors (p65 PDB:5U4K, Foxo3 conformation 1 PDB:2LQI; MLL PDB:2LXS; Myb PDB:1SB0; E2a PDB:2KWF). (b) Overall amino acid composition of KIX ligands (Table S1) and occurrence of hot‐spot residues (%) relative to all identified hot‐spots using computational alanine scanning (BAlaS [31]) (Figure S1).

To test this approach, we selected the KIX domain of the CBP/p300 protein [1, 32, 33], which binds multiple TFs at two different, allosterically coupled sites, denoted as MYB‐site and MLL‐site (Figure 1). It is mainly responsible for haematopoiesis, cell proliferation, differentiation, and survival, and is a possible target in certain types of cancer, such as acute myeloid leukaemia or small cell lung cancer [4, 34, 35, 36, 37]. Despite its high therapeutic potential, the multiple binding sites, the shallow surface of KIX, and the plasticity of its interactions have made inhibitor design approaches especially challenging. Amino acid mutation studies [34, [35], retro‐inverso peptides [38], sulfonyl γ‐peptides [39], small molecule antagonists [40, 41, 42, 43], phage display [44], and disulphide tethering approaches [45, 46] have been described to target the KIX domain, but most of these resulted in weak affinity ligands. Using non‐natural side‐chains has been proven to be efficient in finding cryptic binding pockets on one site of the protein, resulting in improved affinity [46]. Taking advantage of the positive allostery between the MYB‐ and MLL‐sites on KIX, ligands created by coupling native peptides that simultaneously target both sites can reach *K*_D_ values in the low nanomolar to picomolar range [47, 48].

Here, we show that *de novo* designed α/β‐peptide sequences that mimic the overall characteristics of native ligands of the KIX domain can efficiently inhibit its interaction with MLL and Myb transcription factors. Based on the analysis of native interaction partners, α/β‐peptide libraries could be generated with good water solubility in synthetically and analytically manageable sizes. Using non‐natural fluorinated amino acids allowed us to tune the binding site selectivity of the ligands, which have already reached the affinity of the native interaction partners. To exploit multivalency, we create dimeric ligands from the initial hits either by using template‐directed dynamic combinatorial libraries or static libraries focusing on binding site preference. We propose that this approach can generally be applied to nuclear protein targets involved in interactions with IDRs that rely on multivalency.

## 2 Results and Discussion

### 2.1 Design and Screening of α/β‐peptide Libraries

**FIGURE 2:**
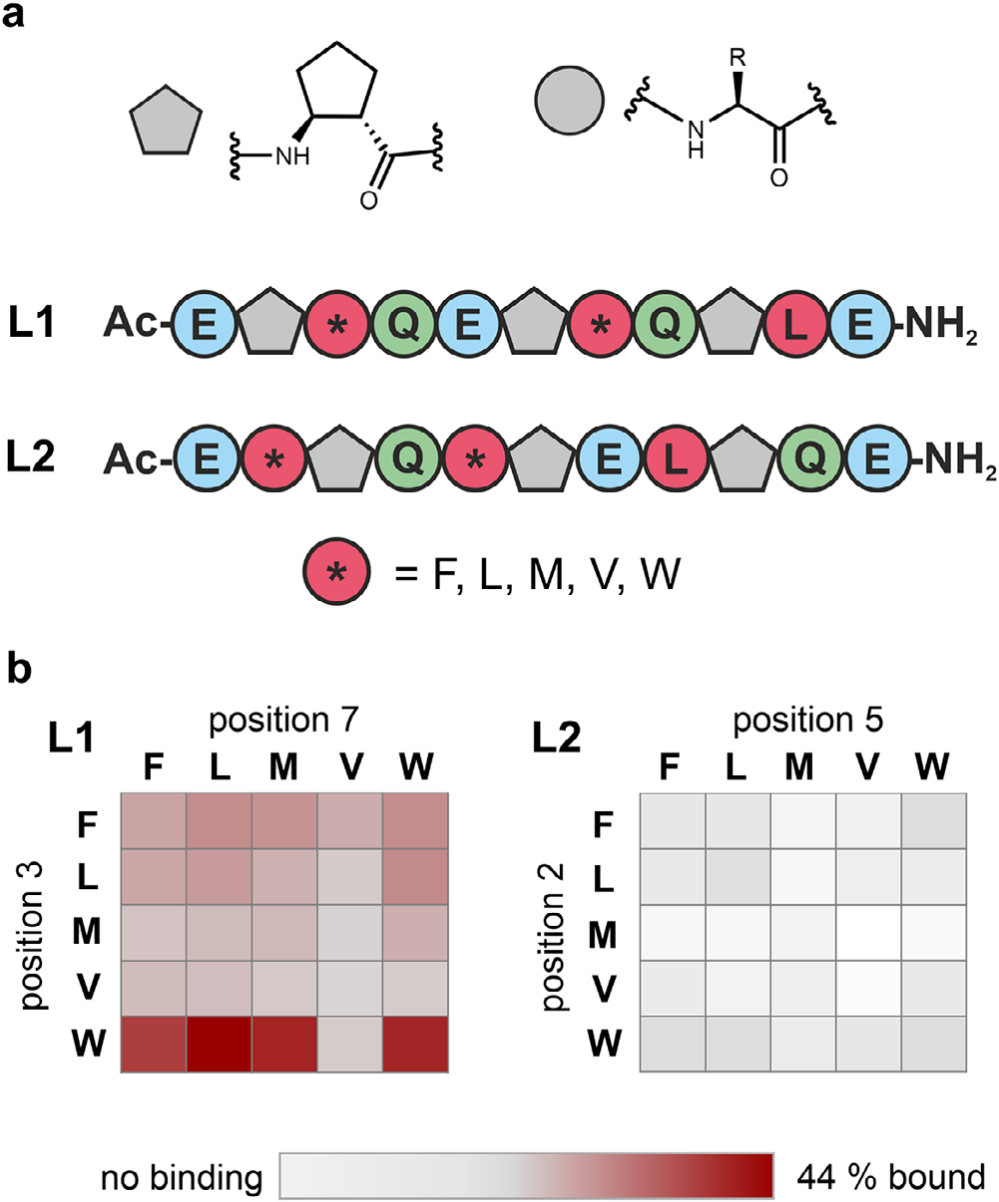
Structure of the foldamer libraries **L1‐L2** and results of the pull‐down assay. (a) Amino acid structures used in the α/β‐peptides, with the pentagon denoting the cyclic ACPC residues and the circles denoting proteinogenic α residues and the sequences of the foldamer peptides. Blue indicates negatively charged, green indicates hydrophilic, and red indicates the hydrophobic or aromatic amino acids serving as hot‐spots for binding. The stars indicate variable positions. (b) Heat maps were constructed based on the variable hot‐spot residues of the library members. The intensity of the cells’ color represents fractions that are bound to the KIX domain. Bound % have been calculated relative to GST control based on the following formula: (AUC_GST_‐AUC_KIX_) / AUC_GST_ *100, where AUC (area under curve) is the integrated area of the LC‐MS chromatogram peak associated with the library member (Figure S2).

To inform our peptidomimetic design targeting the KIX domain, we analysed the overall amino acid composition of multiple ligands and performed computational alanine scanning to identify hot‐spot residues. The KIX domain folds into a globular bundle of three α‐helices that form two distinct binding sites. These interact with disordered ligands, folding into helical structures (Figure 1a), with a positive allosteric effect between the two sites [34, 35, 36, 49]. Among others, the phosphorylated KID domain of CREB and Myb transcription factors are known to bind to the Myb site, while the multiple lineage leukaemia protein (MLL) binds to the opposite (Figure 1a and Table S1). Some proteins, such as the tumour suppressor p53 and FOXO3a, interact with both sites simultaneously, sometimes in multiple possible angles [34, 35, 36, 40]. The amino acid content of KIX binding ligands has a high proportion of hydrophilic and negatively charged residues, which is frequent among disordered activation domains [50, 51] (Figure 1b). In silico alanine scanning was performed using BAlaS [31, 52], which determines the binding energy difference between the native sequence and a single alanine mutant based on available structures. The residues responsible for generating the majority of the binding affinity (ΔΔG > 4 kJ/mol) are mostly hydrophobic, with Leu and Phe being the most common (Figure 1b and S1). On the basis of this, our design principle was the following: construct peptide libraries that i) have a helix‐forming propensity, ii) contain a high proportion of hydrophilic and negatively charged residues, and iii) include hydrophobic/aromatic side chains as hot‐spots. We selected two α/β amino acid scaffolds with the pattern of ααβαααβ heptad repeat (**L1**) and ααβααβ (**L2**) (Figure 2a), which have the ability to form helical structures and exhibit higher resistance to proteases [28, 29, 30]. S,S‐aminocyclopentane‐carboxylic acid (S,S‐ACPC) was applied to enhance helix formation, and Glu and Gln were incorporated into the sequence to mimic the hydrophilicity and negative charge of the native ligands. Three positions were selected to mimic hot‐spot residues using hydrophobic/aromatic amino acids (Figure 2a).

Our goal was to conduct an initial screening that does not necessitate the synthesis of individual peptides and provides an accessible method for selecting binders. We achieved this by synthesising peptide library mixtures and combined it with an affinity‐based selection using a pull‐down assay. For the library synthesis, we optimised a microwave‐assisted protocol for the rapid, efficient synthesis of α/β‐peptide mixtures. For α‐amino acids, standard protocols using DIC and Oxyma coupling reagents and 5 equivalents of amino acid excess (2 min coupling time, double couplings) were implemented [53], while for S,S‐ACPC residues, 3 equivalents with a single 4‐minute coupling were used to lower the required amount of the expensive reagent. At variable positions, amino acid mixtures were coupled, with the total amino acid concentration set at 0.8 equivalents relative to resin loading, with double 4‐minute couplings to account for potential differences in amino acid reactivity. This method yielded libraries with close to uniform incorporation at the mixture positions and resulted in quasi‐equimolar mixtures, where deletion products—primarily from less reactive β‐residues – were readily separated from full‐length peptides by HPLC. Estimated purities of the libraries were between 50–91%, which was acceptable for initial screening purposes (Figures S13–26).

To test the binding of the α/β‐libraries to the KIX domain, pull‐down assays were performed. KIX was immobilized through a GST tag, incubated with the libraries, and the supernatant was analysed using LC‐MS relative to a control in which only the GST tag was bound to the resin. The immobilised protein concentration was chosen to exceed the total peptide concentration of the libraries to avoid competition between ligands and to account for potential concentration differences arising from synthetic differences. The library members which could bind to the KIX domain appeared less abundant in the supernatant. Bound% was calculated for each individual compound based on LC‐MS peak area integration of samples incubated with GST‐KIX relative to the GST control (Figure 2). **L1** with the ααβαααβ pattern resulted in a larger amount of binding fragments, indicating that the amino acid pattern has a strong influence on binding (Figures 2 and S2), which aligns with previous results emphasising the applicability of the heptad repeat pattern in helix mimicry [30]. Hot‐spot residues showed a high preference for Trp at position 3 with aromatic, large hydrophobic residues at position 7. Interestingly, this pattern does not resemble the native hot‐spot distribution of the KIX ligands (Figure 1b). However, it has been shown that substitution with Trp in the MLL sequence can contribute to higher affinity [34].

Isothermal titration calorimetry (ITC) for the best ligand having Trp in position 3 and Leu in positions 7 and 10 (termed **L1‐WLL**) revealed a *K*_D_ of 87 µM (Table 1 and Figure S3), a magnitude higher than the affinity of the native ligands. Native MS measurements showed that the KIX domain binds **L1‐WLL** in 1:1 and 1:2 ratios (Figure S4), indicating a potential interaction with both MLL and Myb binding sites. NMR measurements showed that **L1‐WLL** is folded into a helical conformation, projecting hot‐spot residues from one face of the ligand, with potentially some flexibility at the termini, indicated by the lack of long‐range NOE interactions in those regions (Figure S5).

**TABLE 1:** Binding affinity and thermodynamic parameters of the characterized ligands, determined by ITC. The raw ITC and fitted thermograms are included in the Supplementary Information, Figures S3, S8, and S12. *K*_D_ and Δ*H* values are best‑fit parameters from SEDPHAT; 68% confidence intervals, obtained using the automatic confidence‑interval search (error‑surface projection), are given in parentheses. IC_50_ values determined using fluorescence anisotropy (FA) assays against fluorescently labelled MLL and MYB peptides. IC_50_ values and their errors were obtained by fitting averaged titration curves (n = 3) with a dose‐response model, and the tabulated errors correspond to the standard errors of the fitted IC_50_ parameters, unless otherwise stated.

	*K*_D_ (µM)	Δ*H* (kcal mol^−1^)	ΔS (cal mol^−1^ K^−1^)	IC_50_ competition with Flu‐MLL (µM)	IC_50_ competition with Flu‐MYB (µM)
**MLL**	2.2 (2.0–2.4)	−9.5 (−9.3 to −9.9)	−6.3	5.2 ± 0.3^c^	n.a.
**MYB**	6.3 (6.2–7.4)	−8.2 (−9.4 to −7.3)	−3.7	n.a.	8.9 ± 1.9^c^
**L1–WLL** ^a^	*K*_D1_ = 87 (66–117) *K*_D2_ > 200	Δ*H*_1_ *=* −1.3 (−1.1 to −1.6)	Δ*S*_1_ *=* 14.1	11 ± 0.9	13 ± 2.5
L7–W(F_3_CF)_2_^a^	6.9 (2.5 – 9.4) *K*_D2_ > 500	Δ*H*_1_ *=* −1.8 (−2.1 to −1.7)	Δ*S*_1_ *=* 17.5	14 ± 0.8	35 ± 4
**WLL‐WWL dimer** ^b^	*K*_D1_ = 1.6 (1.2 – 2.3) *K*_D2_ = 31.9 (26.5 – 38.2)	Δ*H*_1_ *=* −3.4 (−3.8 to −3.0) Δ*H*_2_ *=* −7.2 (−7.9 to −6.6)	Δ*S*_1_ *=* 15.2 Δ*S*_2_ *=* −3.6	3.7 ± 0.7	3.7 ± 0.6
**W(F_3_CF)_2_ – WLL dimer** ^a,b^	*K*_D1_ = 2.1 (0.8 – 4.1) *K*_D2_ = 128 (77 – 286)	Δ*H*_1_ *=* −1.65 (−2.4 to −0.8)	Δ*S*_1_ *=* 20.4	8.8 ± 0.96	5.9 ± 0.6

^a^ Due to the low affinity (low c‐value of the ITC experiments), the fitting error for *K*_D2_ and Δ*H*_2_, Δ*S*_2_ could not be accurately determined.

^b^ ITC Fitted globally to forward and reverse titrations (Figure S12).

^c^ Average IC_50_ and standard deviation of two independent experiments (Figures 5 and 6e,f).

**FIGURE 3:**
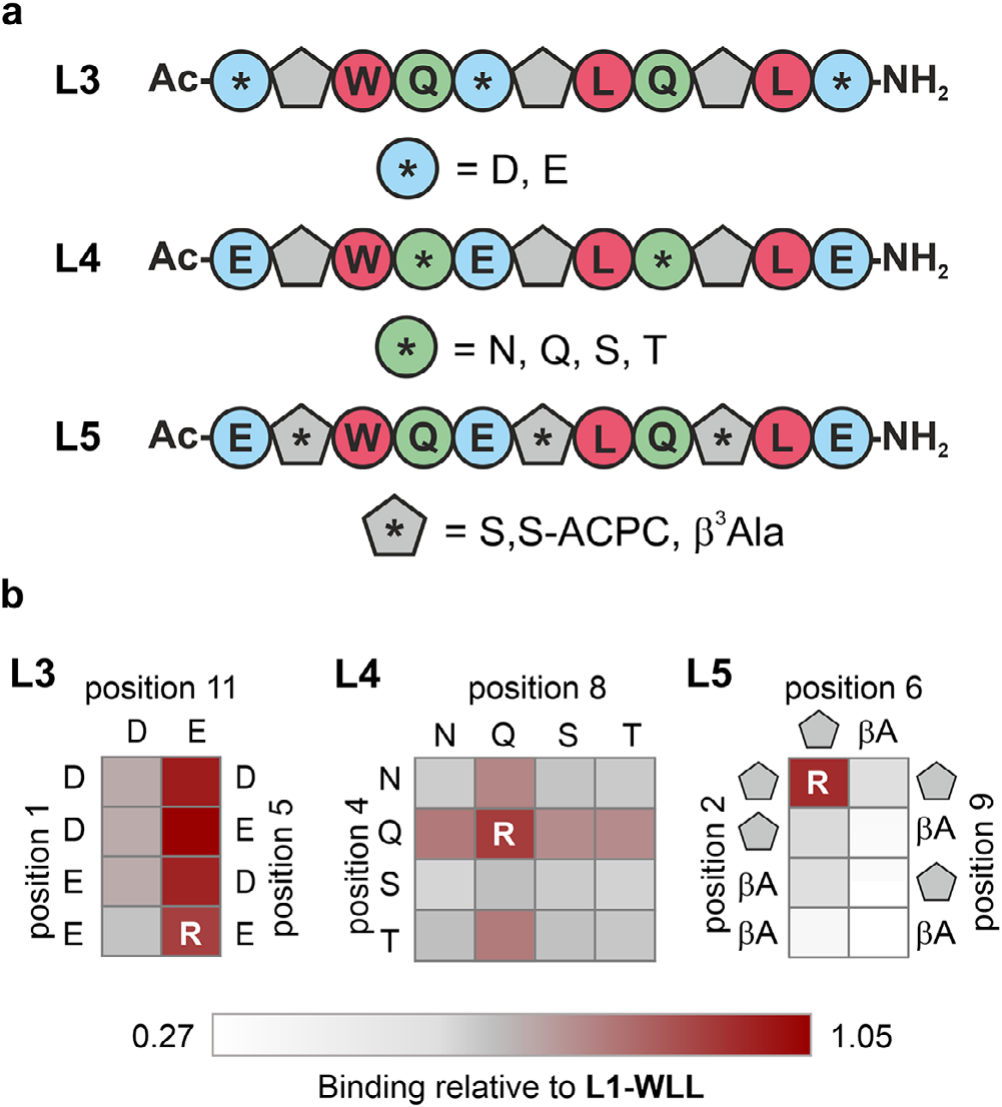
Structure of the foldamer libraries and results of the pull‐down assay. (a) sequences of libraries **L3‐L5**. The hot‐spot residues were fixed as the most successful WLL combination, while the variable positions are indicated by stars. (b) Heat maps representing the results of pull‐down assays, with the coloration of the cells representing the binding success relative to the original **L1‐WLL** fragment. Binding was calculated from the Bound % values relative to the reference **L1‐WLL** fragment, indicated by ‘R’ on the heatmaps (Figure S6).

Next, keeping the hot‐spots fixed, we varied the negatively charged (**L3**), the hydrophilic (**L4**), and the β‐amino acid positions (**L5**) (Figure 3a) to determine whether a better combination of amino acids results in enhanced affinity. For negatively charged residues, position 11 showed a slightly higher preference for glutamic acid, while the other two positions (1 and 5) tolerated aspartic acid as well. The hydrophilic residue screen revealed that sequences containing glutamines at positions 4 and 8 have the highest affinity (Figures 3b and S6). In **L5**, the library members had certain cyclic ACPC amino acids switched to β^3^‐alanine residues, which have decreased helix‐inducing potency, thus resembling more closely the intrinsically disordered native ligands of the KIX domain. However, our results indicate that the presence of cyclic residues is essential for binding. Previous studies have shown that despite the KIX domain's preference for IDRs, enhanced helicity of the ligands may improve their binding affinity [35], which is in line with our observations.

**FIGURE 4:**
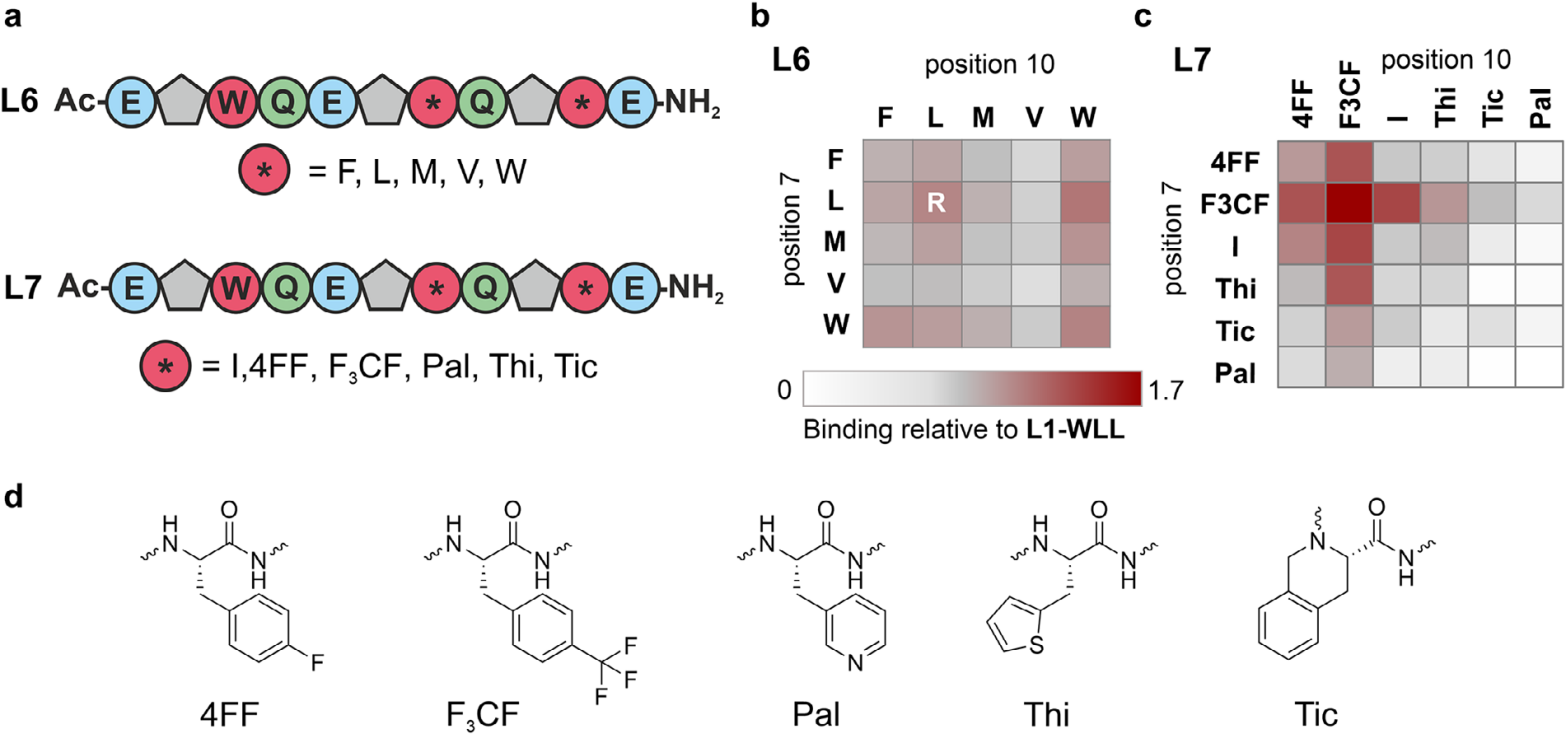
Structure of the foldamer libraries **L6** and **L7** and the results of the pull‐down assays. (a) Sequences of libraries L6 and L7. Bound % values for (b) **L6** and (c) **L7** relative to the original **L1‐WLL** (Bound%_compound_ / Bound%**_L1‐WLL_**) (Figure S7). (d) The molecular structures of the non‐natural side chains of the **L7** library.

Since none of these libraries yielded any significant improvement over the original **L1‐WLL**, we turned toward the optimization of the hot‐spot residues, including non‐natural side chains. **L6** was created to test the third hotspot position, with position 3 now fixed as Trp. A slight improvement was observed with bound % when position 10 was also a Trp (**L6‐WLW**), but it was not significant over the original **L1‐WLL** (Figures 4a,b, and S7). Using non‐natural side‐chains can be advantageous in creating further contacts with the protein surface, due to their ability to reach binding pockets unavailable for proteinogenic side‐chains [46, 54]. The **L7** library, therefore, contained fluorinated and heterocyclic side‐chain modifications (Figure 4c,d). Compared to the **L1‐WLL**, fluorinated amino acid‐containing sequences showed improved binding with the best compound containing trifluoromethyl‐phenylalanine as hot‐spots (**L7‐W(F_3_CF)_2_**), showing 1.7‐fold improvement in binding, relative to **L1‐WLL** (Figures 4c and S7). ITC measurements revealed that **L7‐W(F_3_CF)_2_** binds to the KIX domain with a *K*_D_ of 6.9 µM, which is a 10‐fold improvement over the original hit and closer to the native MLL and Myb sequences (Table 1 and Figure S8)

### 2.2 Influence of Hot‐Spot Composition on Binding Site Selectivity

**FIGURE 5:**
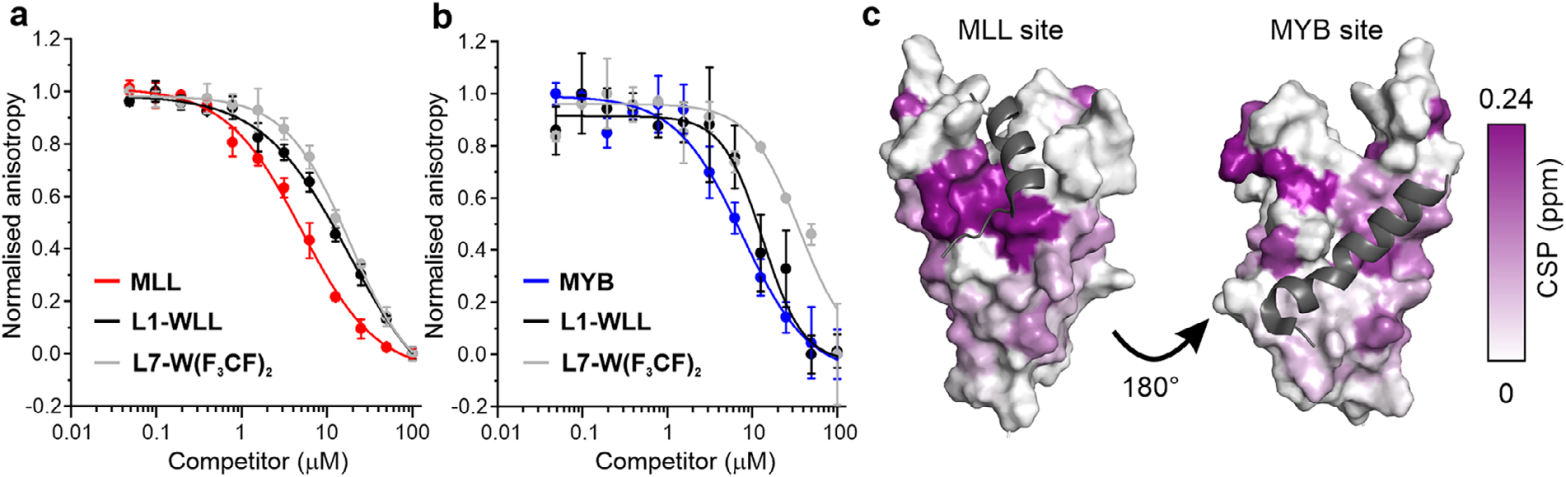
FA competition assays of **L1‐WLL** and **L7‐W(F_3_CF)_2_** against (a) fluorescently labelled MLL and (b) MYB peptides. Measurements were performed using a 3 µM KIX concentration and 50 nM fluorescent tracer with a ½ dilution series of the competitor ligand, starting from 100 µM as the highest concentration, in 40 mM Na‐phosphate, 100 mM NaCl, 0.01% triton X, pH 7 buffer at 25 °C. Each titration point is the mean of triplicates; error bars indicate standard deviation (n = 3). (c) Significant amide chemical shift changes upon addition of 2 equivalent **L7‐W(F_3_CF)_2_** mapped onto the KIX domain structure (PDB: 2AGH) CSP = [(Δδ_H_)^2^ + (0.14Δδ_N_)^2^]^1/2^; CSP_average_ = 0.13 ppm, σ = 0.12 ppm; KIX residues that shifted significantly (CSP > 0.9CSP_average_ + σ) are labeled purple.

With the two best libraries (**L1** and **L7**) in hand, we repeated the pull‐down assays in the presence of MLL or Myb ligands to assess the binding site‐preference of the α/β‐peptides. For both **L1** and **L7,** an approximately 30% decrease in the bound fraction was observed in the presence of 150 µM MLL for the 10 best compounds, indicating a competition between the ligands (Figure S9). In the presence of Myb, the two libraries revealed different behaviour. Bound % for **L1** decreased with an average of 10%, whereas no change, or a slight increase, was observed for the 10 best compounds in **L7** (Figure S9). This indicated that the ligands preferentially bind to the MLL site and that this preference is enhanced when fluorinated amino acids are used. Since the side‐chains of the native MLL do not fully occupy the close pockets [46], using large, more bulky side‐chains reaching these unoccupied surfaces can result in the shift in binding site preference, over the shallower Myb site. To confirm these observations, fluorescence anisotropy measurements were conducted by competing fluorescently labelled MLL or Myb. **L1‐WLL** competed with both ligands, with a slightly higher IC_50_ (11 and 12 µM) in comparison with the native MLL or Myb (Table 1 and Figure 5). In line with the competition pull‐down results, the **L7‐W(F_3_CF)_2_** performed worse in competing with the Myb site, and although it had an overall higher binding affinity to the KIX domain, its competition with the MLL did not improve over **L1‐WLL**. This could indicate a partially shared or allosteric binding site that does not result in efficient competition with MLL. The binding of **L7‐W(F_3_CF)_2_** was characterised by solution NMR using ^13^C‐, ^15^N‐labelled KIX in a ^15^N‐HSQC titration experiment. The calculated chemical shift perturbations (CSPs) using the assigned ^1^H and ^15^N resonances indicate that the peptide affects the entire structure of the KIX domain and may interact with both the MLL and Myb sites (Figures 5c and S10). Larger chemical shift changes were observed for residues at the MLL site, indicating a stronger interaction at this site, which is consistent with our competition data. Nevertheless, an allosteric effect of the peptide on the KIX domain, leading to structural changes detected by NMR, cannot be ruled out.

### 2.3 Affinity Enhancement Through Multivalency

**FIGURE 6:**
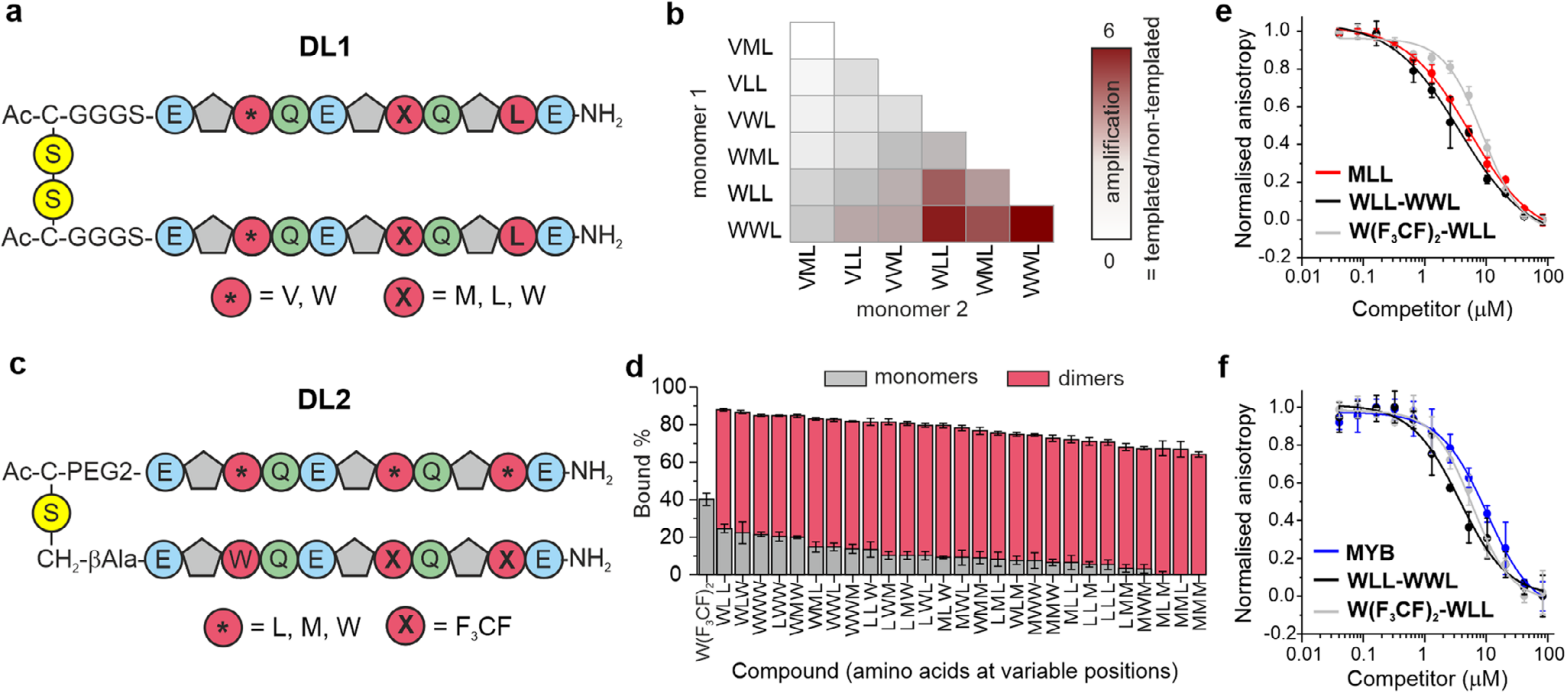
Screening and activity of multivalent ligands. (a) Schematic structure of the disulphide dimers created by DCL from 6 different monomers from **L1**. (b) Results of the DCL experiment, showing amplification factors relative to a nontemplated control. Monomers are denoted with amino acids at the variable positions; the amplification factor was calculated based on LC‐MS peak integration (AUC_templated_/AUC_control)_ after 4 days of equilibration (Figure S11). (c) Schematic structure of **DL2** dimers. (d) Results of the pull‐down assay for **DL2** showing the average bound% for the individual monomers and dimers. Error bars represent the standard deviation obtained from two independent experiments (n = 2). (e) FA assay of selected dimers against Flu‐MLL ligand. (f) FA assay of selected dimers against the Flu‐MYB ligand. Measurement conditions are the same as in Figure 5. Each titration point is the mean of triplicate measurements; error bars represent standard deviation (n = 3).

Multivalency is a common feature of disordered TFs interacting with their target protein [55]. The KIX domain itself has ligands that interact with both binding sites (Figure 1a), in multiple different conformations [36]. Linking MLL and Myb to create hybrid peptides was shown to be an efficient way to obtain selective, high‐affinity ligands [47, 48]. To create multivalent ligands from the libraries presented here, we tested two different approaches in parallel: i) a template‐directed assembly of dimers (**DL1**) and ii) a static library of dimers focusing on binding site preference (**DL2**). Linker lengths between the two monomers were selected based on previous studies [47, 48], resulting in spacers comprising 32 or 24 nonhydrogen atoms between the monomers for **DL1** and **DL2,** respectively (Figure 6).

In the template‐directed approach, a disulphide exchange‐based dynamic covalent chemistry (DCC) strategy was used [22, 56]. DCC has the advantage of an in situ synthesis of a diverse library from monomers that are in constant exchange with each other in an equilibrium mixture, which can be shifted by the addition of a template toward the best binders. Six monomers were selected from the **L1** library containing valine, leucine, and tryptophan hot‐spot residues, and synthesised with a CGGGS linker, with cysteine at the N terminus (**DL1**, Figure 6a). The compounds were mixed in a glutathione redox buffer, allowing the formation of disulfide dimers in an equilibrium reaction. KIX was added as the protein template, and the mixtures are analysed by LCMS relative to a nontemplated control experiment, from which amplification factors were determined. The best dimers showed a sixfold amplification after 4 days of equilibration compared to a nontemplated experiment (Figures 6b and S11). These included monomers enriched in tryptophan and leucine residues, which is in line with their previously observed higher affinity relative to the other selected compounds. For further characterization, we synthesised a dimer from **L1‐WLL** and **L1‐WWL** using a more stable thioether linkage (**WLL‐WWL**).

In our second approach, heterodimers were created by fixing one of the monomers as the **L7‐W(F_3_CF)_2_** equipped with a chloroacetic acid moiety and coupled to a library of thiol‐containing peptides (**DL2,** Figure 6c), including the best combinations of side chains from the original **L1** library. The rationale behind this design was that the higher affinity **L7‐W(F_3_CF)_2_**, which has a preference for the MLL site, could act as a tether for the lower affinity fragment that could interact with the Myb site. The generated dimers showed a fourfold improvement in bound % values compared to the monomers, in our pull‐down assays (Figure 6d), with **W(F_3_CF)_2_‐WLL** dimer as the highest affinity combination.

ITC measurements showed that both the **WLL‐WWL** and the **W(F_3_CF)_2_‐WLL** dimers have improved affinity for the KIX domain (Table 1 and Figure S12), indicating that the chosen linker lengths are appropriate for enhancing binding. The titrations revealed that the dimers interact with a higher affinity 1:1 interaction with the KIX domain, which is followed by a second, lower affinity binding step. This may be due to the dimers binding simultaneously to the KIX domain in two different conformations or possibly through crosslinking between multiple ligands and domains via opposite binding sites. In FA competition assays, both dimers competed efficiently with the MLL and Myb ligands, showing IC_50_ values close to those of the native ligands (Figure 6e,f, and Table 1), with a more pronounced improvement in competition at the Myb site. These data indicate that both strategies can be beneficial for creating multivalent ligands efficiently targeting multiple binding sites simultaneously.

## 3 Discussion

Therapeutically relevant PPIs within the nucleus present challenging targets for drug development due to the high prevalence of interactions involving complex molecular mechanisms often relying on multivalency [4, 5, 6]. In addition, the size of the nuclear pores further limits the effective delivery of therapeutics. Peptidomimetics offer a valuable approach to targeting these PPIs due to their ability to recreate protein‐like binding at a smaller molecular weight [13, 14, 15].

Despite the wide variety of available peptidomimetic scaffolds, the *de novo* design strategies that result in close mimicry of the native ligands remain challenging, especially when cooperative binding sites are involved. Previous strategies primarily focus on projecting hot‐spot residues from rigid, prearranged scaffolds, targeting a single binding site on the protein, and often result in ligands with solubility and selectivity issues. Our hypothesis was to use a less ordered scaffold that shows helix formation propensity, use amino acids that reproduce the overall physicochemical properties of the native ligands, and implement steps for optimising solvent‐exposed residues. After an initial screening of potential binders followed by validation and determination of binding site preference, a multivalent ligand can be constructed to improve affinity.

We achieve this by creating short α/β‐peptide libraries and modularly optimising structure‐inducing, hot‐spot, and solvent‐exposed residues. The use of cyclic beta amino acid residues in a pattern ααβαααβ or ααβααβ produces sequences that have a general propensity to form helices and protect the peptides against potential proteolytic degradation [28, 29, 30]. The use of mixed sequences has the additional advantage of broadening the chemical space by incorporating α‐amino acids with non‐natural side‐chains, otherwise unavailable for homologues. To ease the initial screening process, we used a library mixture synthesis combined with an affinity‐based pull‐down assay, which does not necessitate the isolation of individual peptides at the screening stage and provides a fast method for obtaining information on the best amino acid combinations.

Initial screening efforts revealed that the heptad repeat (ααβαααβ) amino acid pattern is crucial for binding (Figure 2). We continued to optimise hot‐spot and solvent‐exposed residues using this pattern. With the help of fluorinated side chains, we were able to tune the binding site preference of the ligands, with the best monomer (**L7‐W(F_3_CF)_2_**) showing a *K*_D_ of 6.9 µM to the KIX domain (Figure 5 and Table 1). As a next step, we took advantage of multivalency and created dimeric ligands using two different approaches. The template‐directed dynamic covalent assembly of the dimers allowed the synthesis and selection of the ligands in a single step, resulting in the amplification of the best dimers in the presence of KIX as the template (Figure 6a,b), providing a valuable tool for the assembly of multivalent peptides. In our second approach, a static library was synthesised using the highest affinity, MLL‐site selective monomer as a tether, coupled to a library of monomers to target the opposite binding sites of the protein (Figure 6c,d). Both approaches yielded enhanced affinity ligands (*K*_D_ = 1.6–2.2 µM, Table 1) and better competition efficiency with native interaction partners (Figure 6e,f), with further optimization of linker length and chemistry likely to provide additional improvements.

In conclusion, our *de novo* design strategy using α/β‐peptide scaffolds as building blocks of multivalent ligands is a promising approach to target proteins with multiple binding sites. This approach allows for the fine‐tuning of residues that are not in direct contact with the protein, which was often neglected in previous designs. Moreover, by combining the readily available α‐ and β‐amino acid residues, modified side chains can be incorporated, resulting in enhanced properties of the ligand. We believe that our strategy is advantageous for targeting nuclear proteins involved in the transcription machinery, where structural plasticity, cooperativity, and multivalency are critical.

## Conflicts of Interest

The authors declare no conflicts of interest.

## Supporting information



Supplementary figures and tables, materials and methods, and compound characterization data. The authors have cited additional references within the Supporting Information [57, 58, 59, 60, 61, 62].
