# Supplementary material for: De Novo Design of Multivalent α/β‐Peptides Mimicking Transcription Factors Targeting the CBP KIX Domain
Source: Chemistry. 2026 Jun 2;32(35):e71218. doi: 10.1002/chem.71218 (PMC13587646; doi:10.1002/chem.71218)
Supplement: Supplementary file 1 [file CHEM-32-e71218-s001.pdf]

Supplementary Information for

De Novo Design of Multivalent α/β-Peptides Mimicking Transcription Factors Targeting the CBP KIX Domain

Márk V. Tresztián[a], Vencel L. Petrovicz[a], Edit Wéber[a,b], Tamás A. Martinek[a,b], Zsófia Hegedüs[a]*
[a] University of Szeged, Department of Medical Chemistry, 8 Dóm tér, Szeged, 6720 Hungary
[b] HUN-REN SZTE Biomimetic Systems Research Group, 8 Dóm tér, Szeged, 6720 Hungary
hegedus.zsofia@med.u-szeged.hu

Table of contents

1 Supplementary Figures and Tables 3

Fig. S1 Computational alanine scan results of KIX-interacting ligands using BAlaS[1]. 4
Fig. S2 Pull-down results for L1 and L2 5
Fig. S3 Raw ITC data and fitted thermogram for direct binding of MLL, MYB and L1-WLL. 6
Fig. S4 Native MS measurement of KIX in complex with L1-WLL 6
Fig. S5 ^1H assignment of L1-WLL and detected long-range interactions. 7
Fig. S6 Pull-down results for L3-L5 8
Fig. S7 Pull-down results for L6-L7 9
Fig. S8 Raw ITC data and fitted thermogram for direct binding of L7-W(F_3CF)$_2$ 10
Fig. S9 Screening of L1 and L7 for binding to CBP KIX in presence of MLL or MYB ligands 10
Fig. S10 ^{15}N HSQC spectra of titrating L7-W(F_3CF)$_2$ to CBP KIX 11
Fig. S11 Structure of the disulfide dimers and results of the DCL experiment. 12
Fig. S12 Raw ITC data and fitted thermogram for direct binding of dimeric ligands to KIX 13

2 Materials and Methods 14

2.1 Protein expression 14
2.2 Peptide synthesis 14

2.2.1 Acetylation/Chloroacetylation 15
2.2.2 Fluorescent labelling 15
2.2.3 Cleavage 15
2.2.4 Peptide purification 15
2.2.5 Thioether conjugation 16

2.3 LC-MS characterization and peak area integration of peptide libraries 16
2.4 Pull-down assay 16
2.5 Isothermal titration calorimetry 17
2.6 Fluorescence anisotropy 17
2.7 Native MS measurements 18
2.8 NMR measurements 18

2.8.1 Secondary structure determination 18
2.8.2 Binding site determination 18

2.9 Dynamic covalent chemistry 18

3 Library characterization 19

Fig. S13 LCMS chromatograms of L1. 19
Fig. S14 Estimated equimolarity of L1. 21
Fig. S15 LCMS chromatograms of L2 22
Fig. S16 Estimated equimolarity of L2 24
Fig. S17 LCMS chromatograms of L3 25
Fig. S18 Estimated equimolarity of L3. 26
Fig. S19 LCMS chromatograms of L4. 27
Fig. S20 Estimated equimolarity of L4 28
Fig. S21 LCMS chromatograms of L5 29
Fig. S22 Estimated equimolarity of L5). 30
Fig. S23 LCMS chromatograms of L6. 31
Fig. S24 Estimated equimolarity of L6. 33
Fig. S25 LCMS chromatograms of L7 34
Fig. S26 Estimated equimolarity of L7 36

4 Compound characterization 40
5 References 48

1 Supplementary Figures and Tables

Table S1. Transcription factor activation domain sequences that are used to determine the overall amino acid composition for KIX binding ligands, and available PDB structures used for computational alanine scanning (Fig. S1)

Name	Sequence	PDB	Used for Ala scanning
p65 (RelA)	PGYPNGLLSGDEDFSSIADMDFSALLSQISS	5U4K	yes
MLL	GNILPSDIMDFVLKNTP	2LXS	yes
cMYB	KEKRIKELELLLMSTENELKGQQAL	1SB0	yes
E2a	GTDKELSDLLDFSAMFS	2KWF	yes
FOXO3a CR2C – CR3	SMSHYGNQTLQDLLTSDSLSHSD…LECDMESIIRSELMDA	2LQH (conformation1)	yes
FOXO3a CR2C – CR3	SMSHYGNQTLQDLLTSDSLSHSD…LECDMESIIRSELMDA	2LQI (conformation 2)	yes
p53 AD1	LSQETFSDLWKLLPE	n.a.	no
p53 AD2	MLSPDDIEQWFTEDPG	n.a.	no
CREB pKID	TDSQKRREILSRRPSYRKILNDLSSDAP	1KDX	no
c-JUN	LASPELERLIIQSSN	n.a.	no
HTLV-1 Tax	YIDGRVIGSALQFLIPRLP	n.a.	no

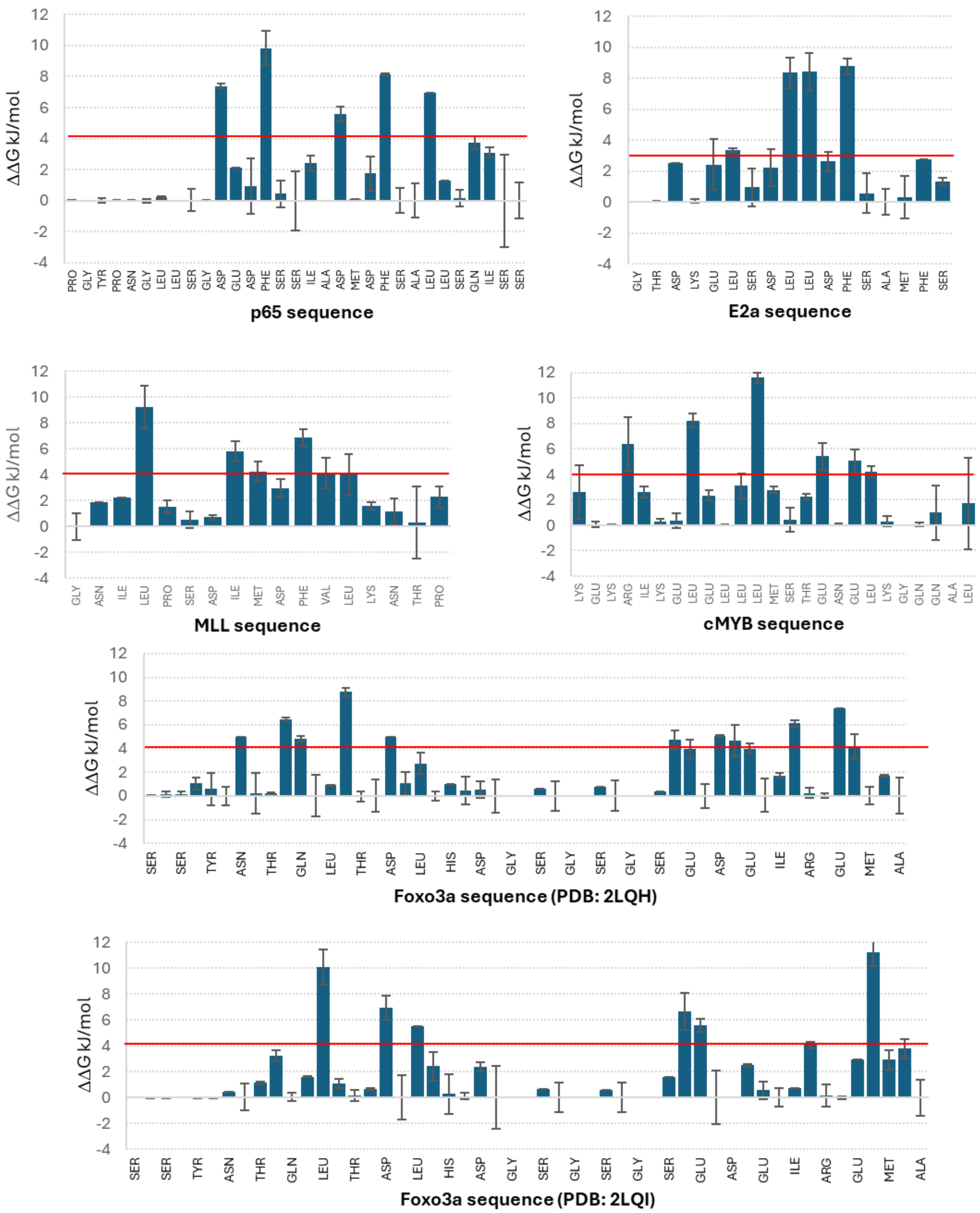

Fig. S1 Computational alanine scan results of KIX-interacting ligands using BAlaS[1].
The following PDB structures were used: p65 PDB:5U4K, Foxo3 conformation 1 PDB:2LQI; Foxo3 conformation 2: 2LQH, MLL PDB:2LXS; cMYB PDB:1SB0; E2a PDB: 2KWF. All structures are NMR ensembles; error bars represent the standard deviation of ΔΔG calculated for all structures in the ensemble. Hot-spot residues are defined as ΔΔG > 4kJ/mol, indicated by a red line.

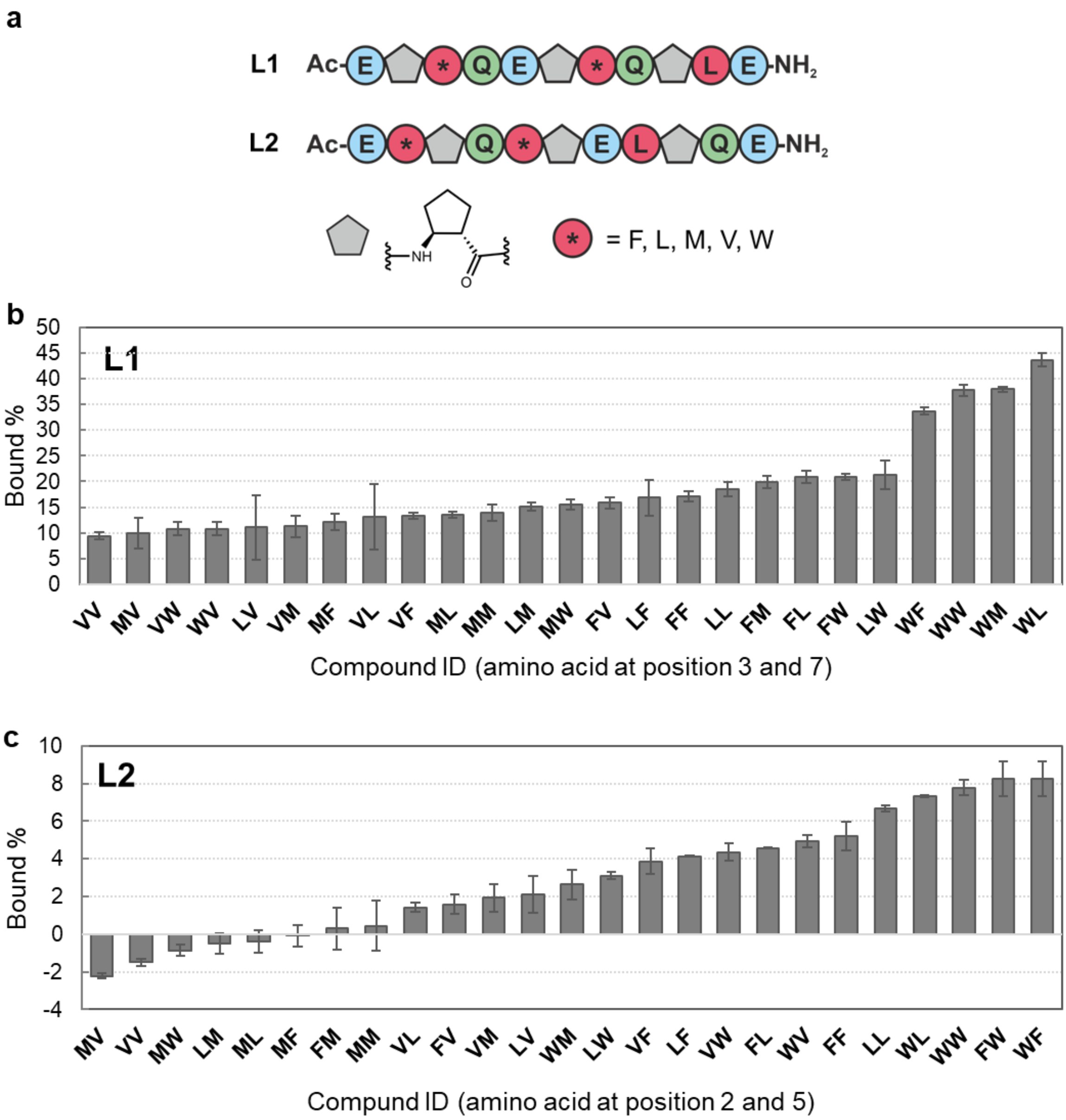

Fig. S2 Pull-down results for L1 and L2

a) Structure of **L1** and **L2**, stars indicate variable positions in which the following amino acids were used: F, L, V, M, W. b) Screening of **L1** and **L2** for binding to CBP KIX using a pull-down assay, libraries were incubated with GST or GST-KIX bound to glutathione magnetic beads, the supernatant was analysed using LC-MS. Bound % have been calculated based on LC-MS peak area integration (AUC) using the following formula: $(AUC_{GST}-AUC_{KIX}) / AUC_{GST}$ *100, the error bars represent the standard deviation of duplicate measurements. Negative values are attributed to nonspecific binding. The compound ID is based on the variable position amino acids.

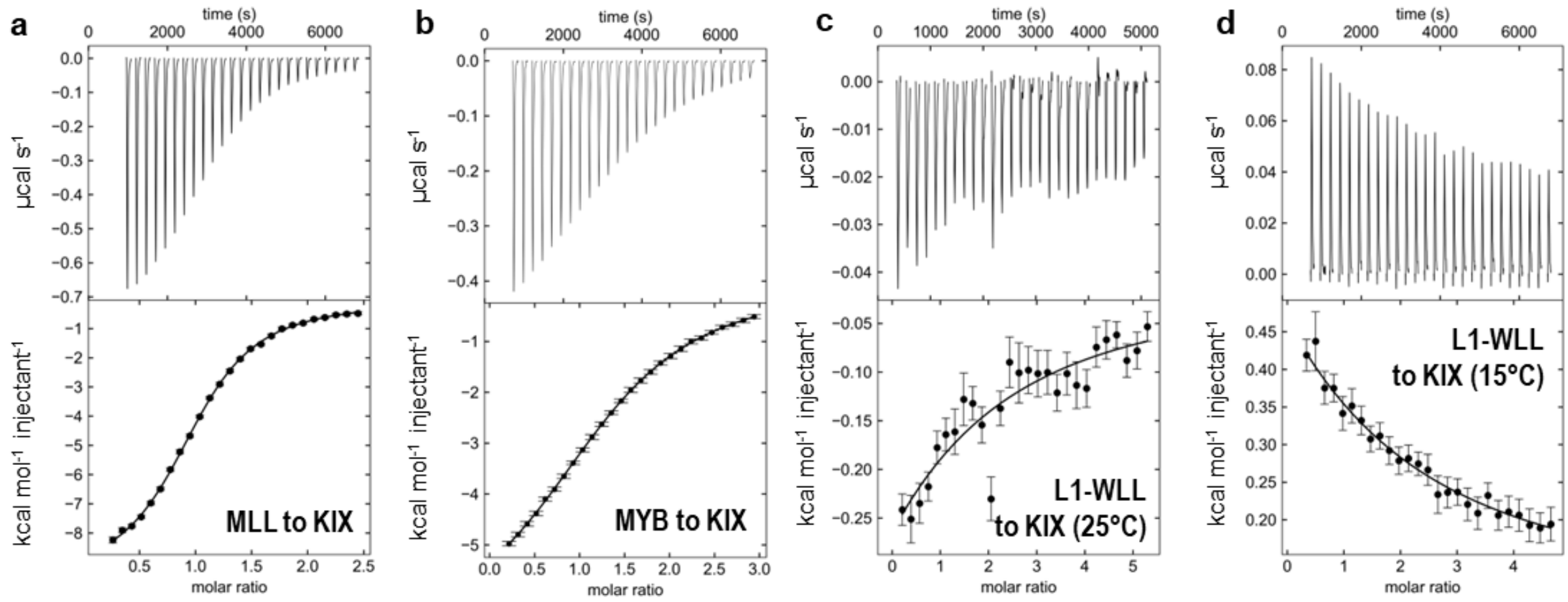

Fig. S3 Raw ITC data and fitted thermogram for direct binding of MLL, MYB and L1-WLL.
a) MLL b) MYB c) **L1-WLL** titrated to KIX at 25°C and d) **L1-WLL** to KIX at 15°C to KIX. Error bars on the thermograms represent integration error, estimated by NITPIC. For the MLL and MYB interactions data were fitted using Sedphat to a 1:1 interaction hetero-association model for MYB and MLL. For **L1-WLL** interaction data were fitted to a 1:2 interaction model, with two non-symmetric sites. All fittings included baseline and incompetent protein fraction fit. Titrations were performed in 50 mM Na-phosphate, 50 mM NaCl, 1 mM DTT, pH 7 buffer. Thermodynamic parameters are summarised in Table 1 for a-c, thermodynamic parameters for d: K_{D1} = 99 µM (89-111) K_{D2} > 200 µM ΔH_1 = 1.8 (1.7-1.95) kcal/mol, ΔS_1 = 24.7 cal/mol/K.

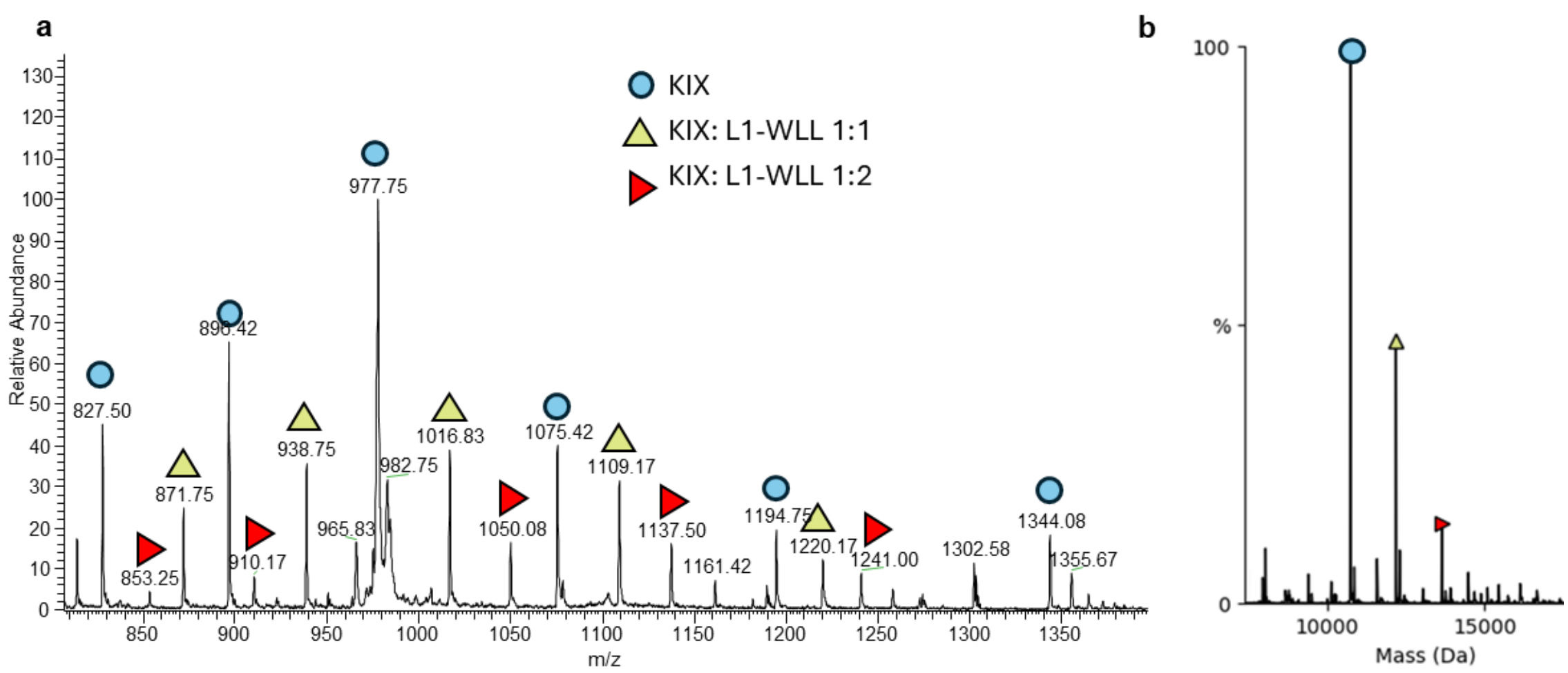

c

	Expected Mass (Da)	Detected Mass (Da)	Relative intensity (%)
KIX	10 743.3	10 744	100.0
KIX: L1-WLL 1:1	12 191.6	12 191	47.2
KIX: L1-WLL 1:2	13 640.2	13 639	14.2

Fig. S4 Native MS measurement of KIX in complex with L1-WLL
a) Raw MS data of 3 µM KIX mixed with 3 equivalents of **L1-WLL** in 10 µM NH_4Ac pH7, using direct infusion. b) Deconvoluted MS spectrum using UNIDEC software[2] showing the native KIX, 1:1 and 1:2 complexes with **L1-WLL**. c) Expected and detected (deconvoluted) mass of the protein and complexes, with relative intensity rations calculated by UNIDEC.

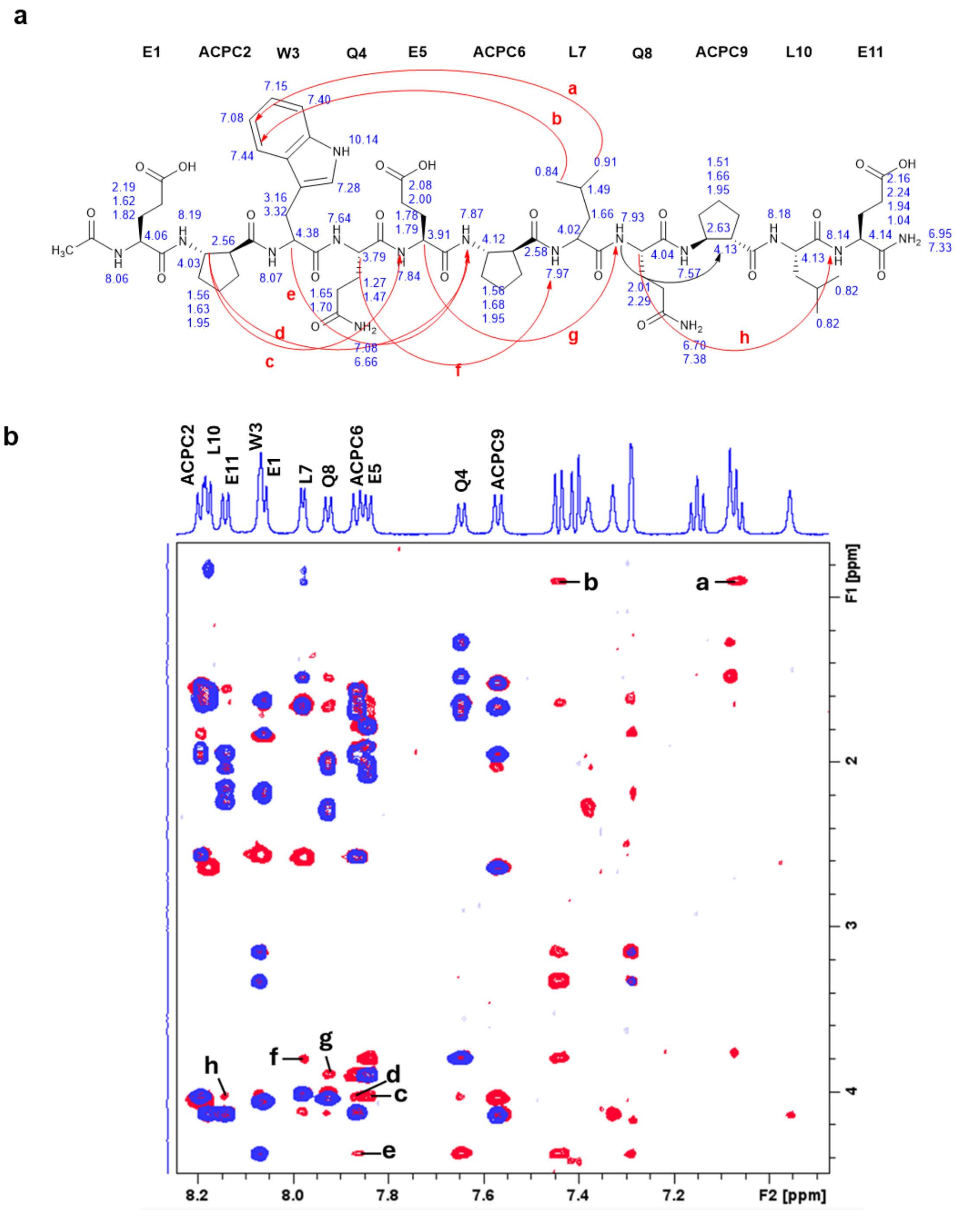

Fig. S5 ^{1}H assignment of L1-WLL and detected long-range interactions.

a) Structure of **L1-WLL** with assigned ^{1}H chemical shifts, red arrows indicate detected long range NOE interactions. b) Amide region of overlayed TOCSY (blue) and ROESY (red) spectra of **L1-WLL**. Letters correspond to the detected long range NOE interactions indicated on the structure.

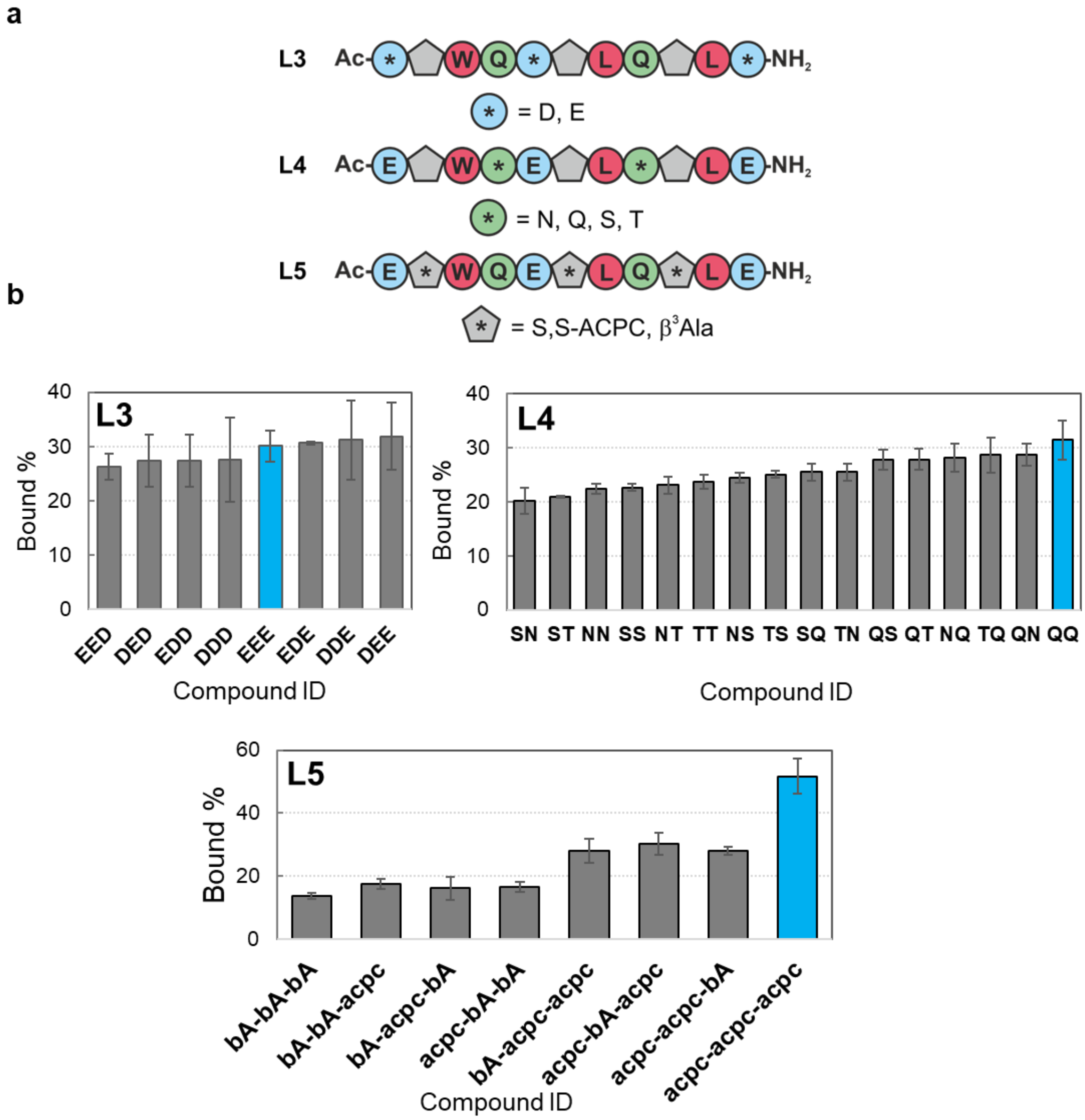

Fig. S6 Pull-down results for L3-L5

a) Structure of **L3**, **L4** and **L5**, stars indicate variable positions where the following amino acids were used: F, L, V, M, W. **b)** Screening of **L3**-**L5** for binding against CBP KIX using pull-down assay, libraries were incubated with GST or GST-KIX bound to glutathione magnetic beads, the supernatant was analysed using LC-MS. Bound % have been calculated based on LC-MS peak area integration (AUC) using the following formula: $(AUC_{GST}-AUC_{KIX}) / AUC_{GST} *100$, error bars represent the standard deviation of duplicate measurements.

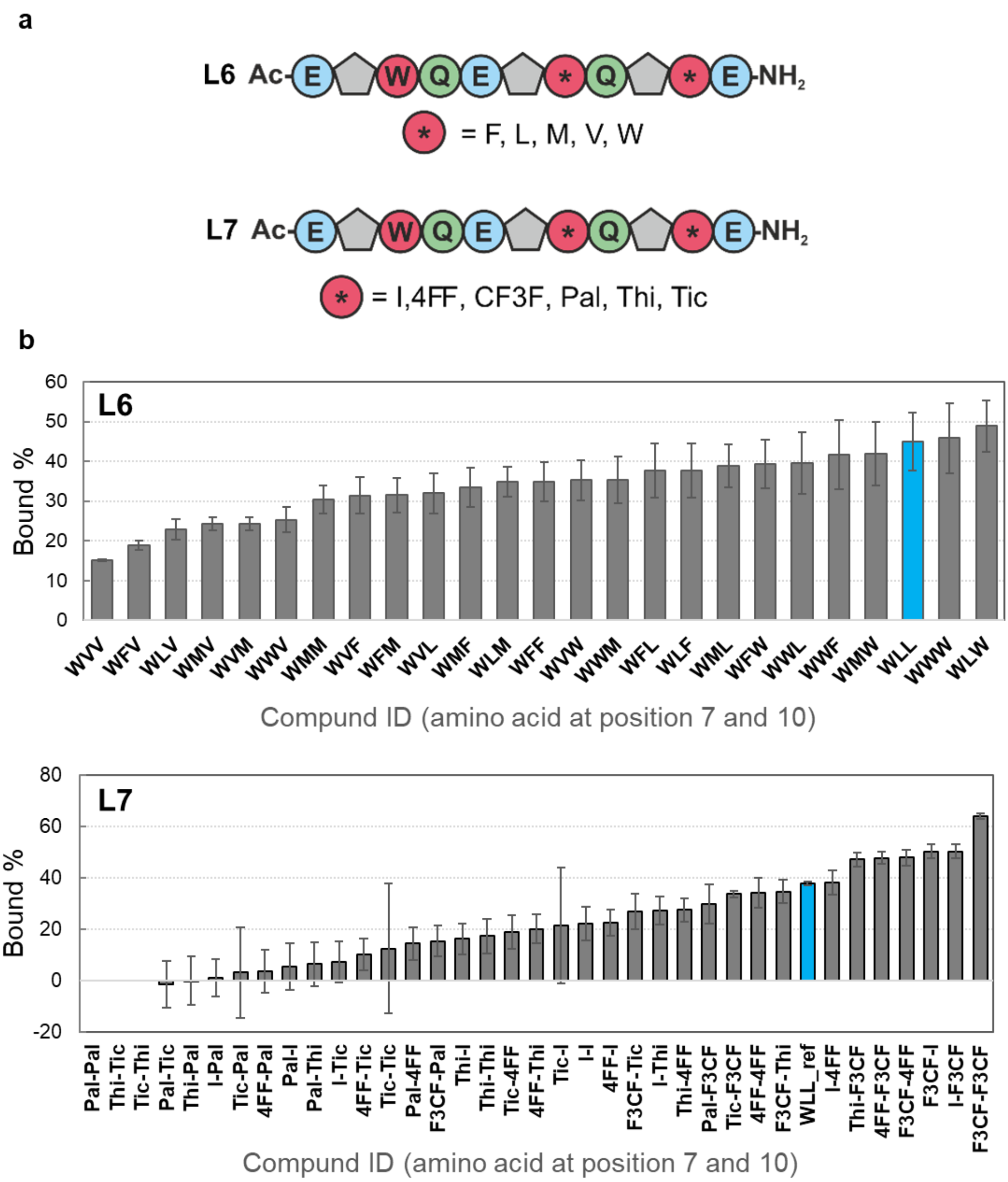

Fig. S7 Pull-down results for L6-L7.

Structure of **L6** and **L7**, stars indicate variable positions. b) Screening of **L6** and **L7** for binding against CBP KIX using pull-down assay, libraries were incubated with GST or GST-KIX bound to glutathione magnetic beads, the supernatant was analysed using LC-MS. Bound % have been calculated based on LC-MS peak area integration (AUC) using the following formula: $(AUC_{GST}-AUC_{KIX}) / AUC_{GST} *100$, error bars represent the standard deviation of duplicate measurements. I: isoleucine, Pal: 3-(3-pyridyl)-L-alanine, Thi: 3-thienyl-L-alanine, Tic: (S)-1,2,3,4-tetrahydroisoquinoline-3-carboxylic acid, 4FF: 4-fluorophenylalanine, F3CF: 4-trifluoromethyl-L-phenylalanine.

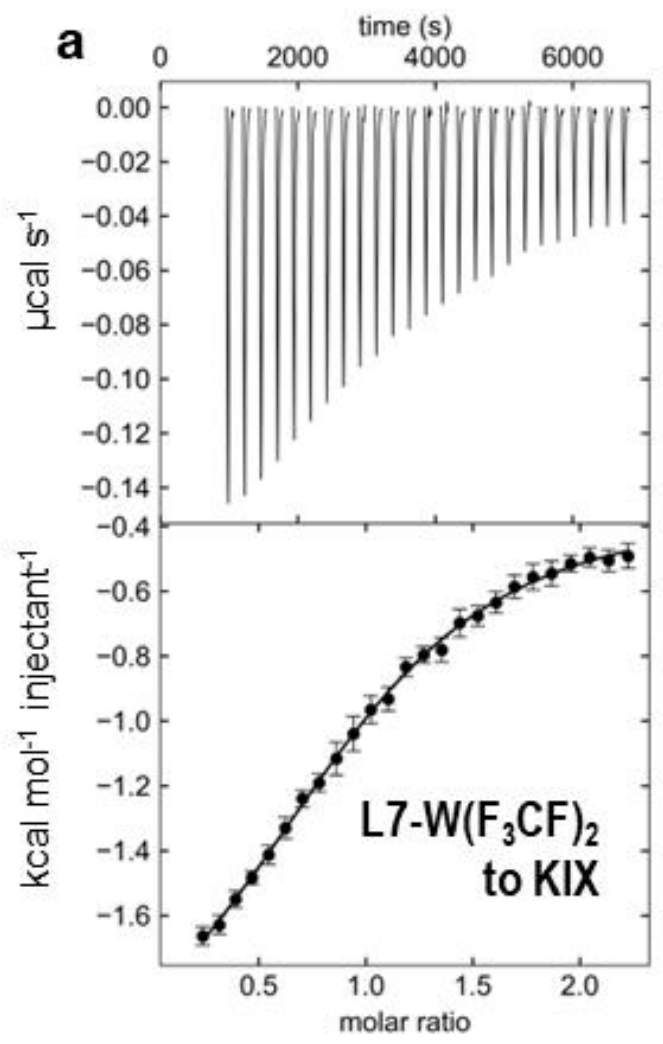

Fig. S8 Raw ITC data and fitted thermogram for direct binding of **L7-W(F$_3$CF)$_2$** to CBP KIX. Titrations were performed in 50 mM Na-phosphate, 50 mM NaCl, 1 mM DTT, pH 7 buffer at 25°C**.** Heats per injection were obtained by NITPIC integration; error bars represent NITPIC-estimated integration errors for each injection

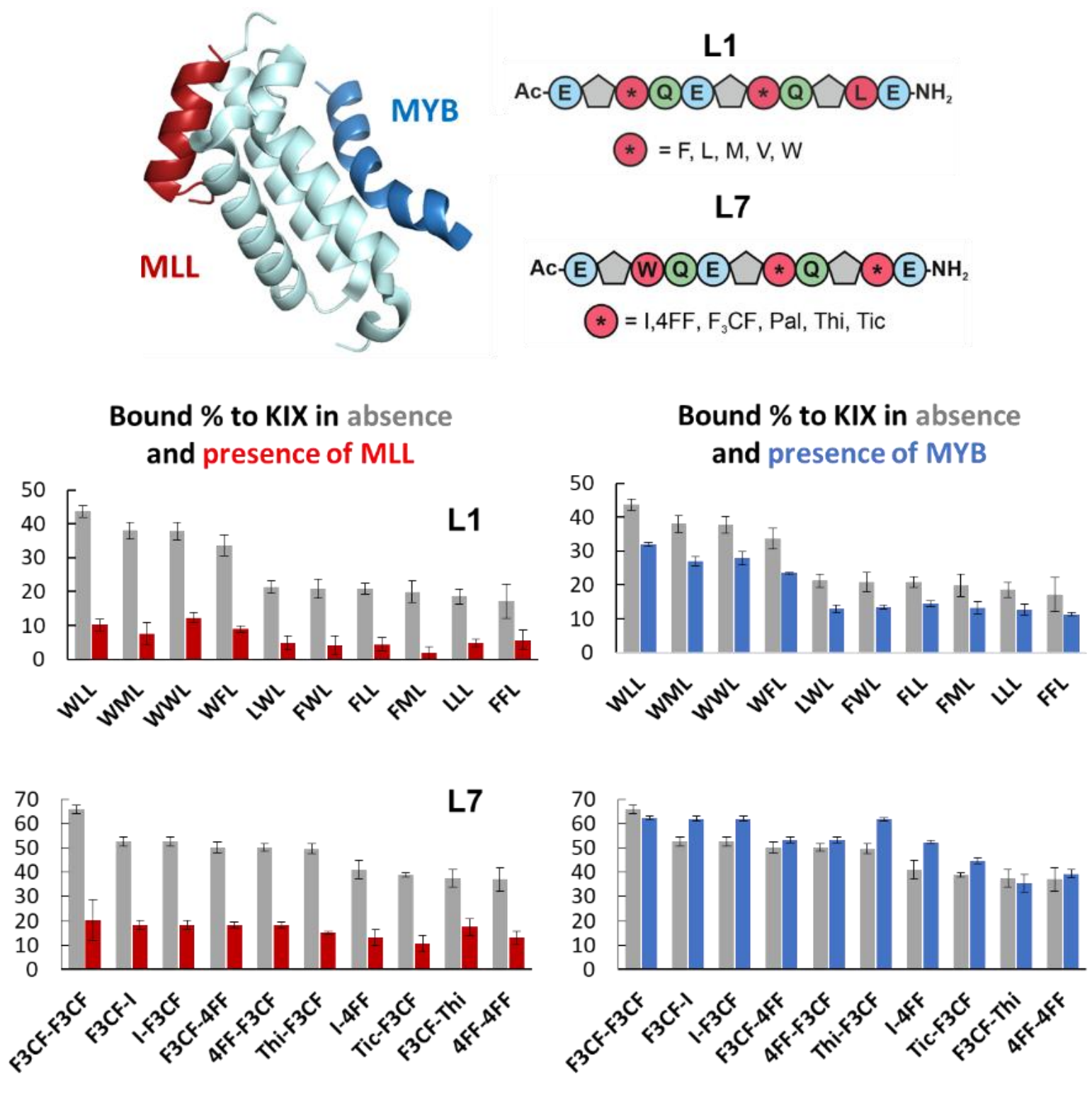

Fig. S9 Screening of L1 and L7 for binding to CBP KIX in presence of MLL or MYB ligands
Libraries were incubated with GST or GST-KIX bound to glutathione magnetic beads, in presence of 150 µM MLL or MYB, the supernatant was analysed using LC-MS. Bound % have been calculated based on LC-MS peak area integration (AUC) using the following formula: (AUC$_{GST}$-AUC$_{KIX}$) / AUC$_{GST}$ *100, error bars represent the standard deviation of duplicate measurements. Compounds are indicated by their hot-spot amino acids.

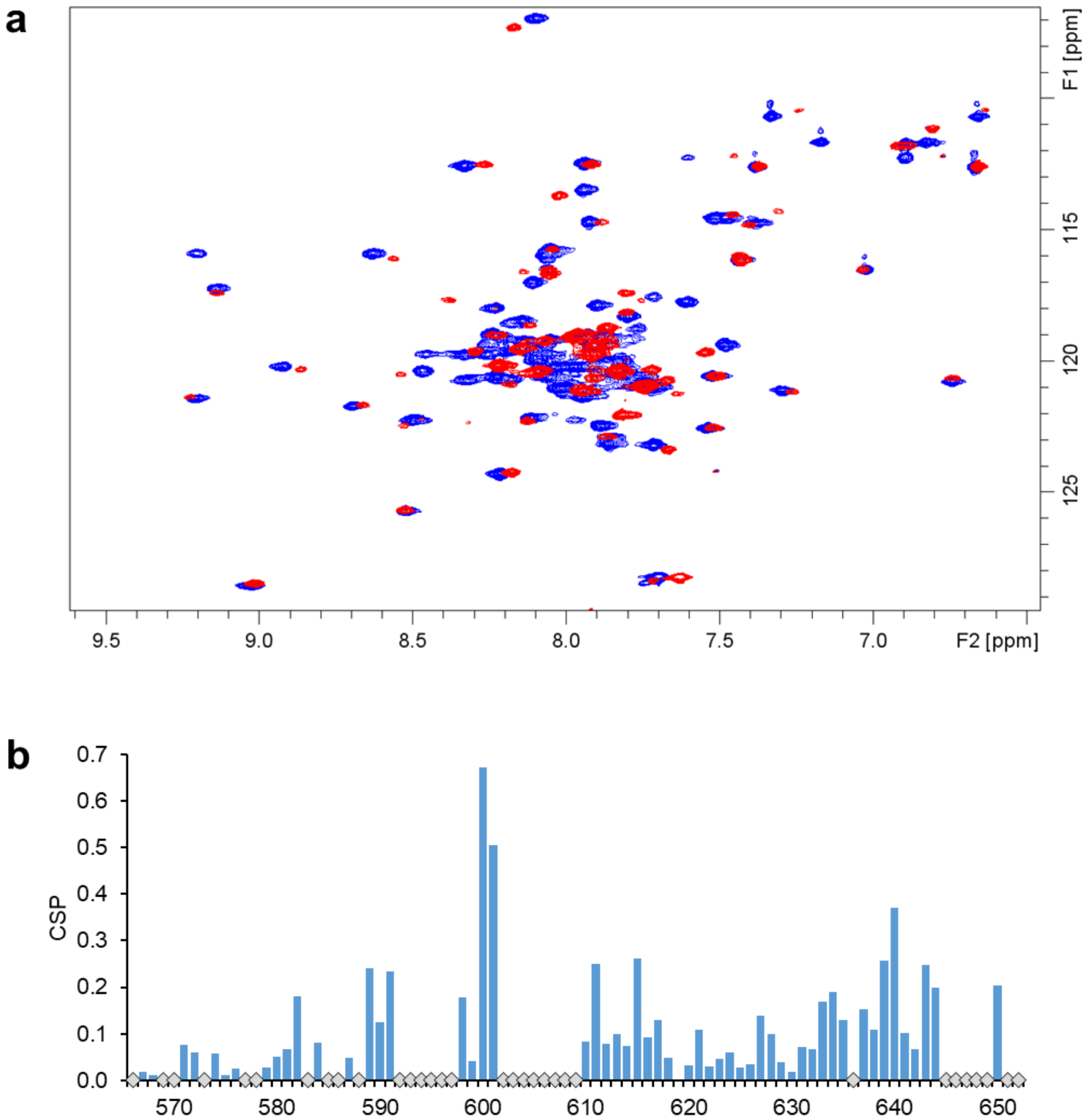

Fig. S10 **^{15}N HSQC spectra of titrating L7-W(F_3CF)$_2$ to CBP KIX.** a) Overlaid 2D ^{15}N-^{1}H HSQC spectra of apo KIX (blue) and KIX in the presence of 2 equivalents of **L7-W(F_3CF)$_2$** (red). b) Chemical shift perturbations (CSPs) of KIX residues upon addition of the ligand. Residues that could not be assigned due to overlapping or missing signals are marked with a diamond. CSP values were calculated using the following formula: $CSP = \sqrt{[\Delta\delta_H^2 + 0.14\Delta\delta_N^2]}$.

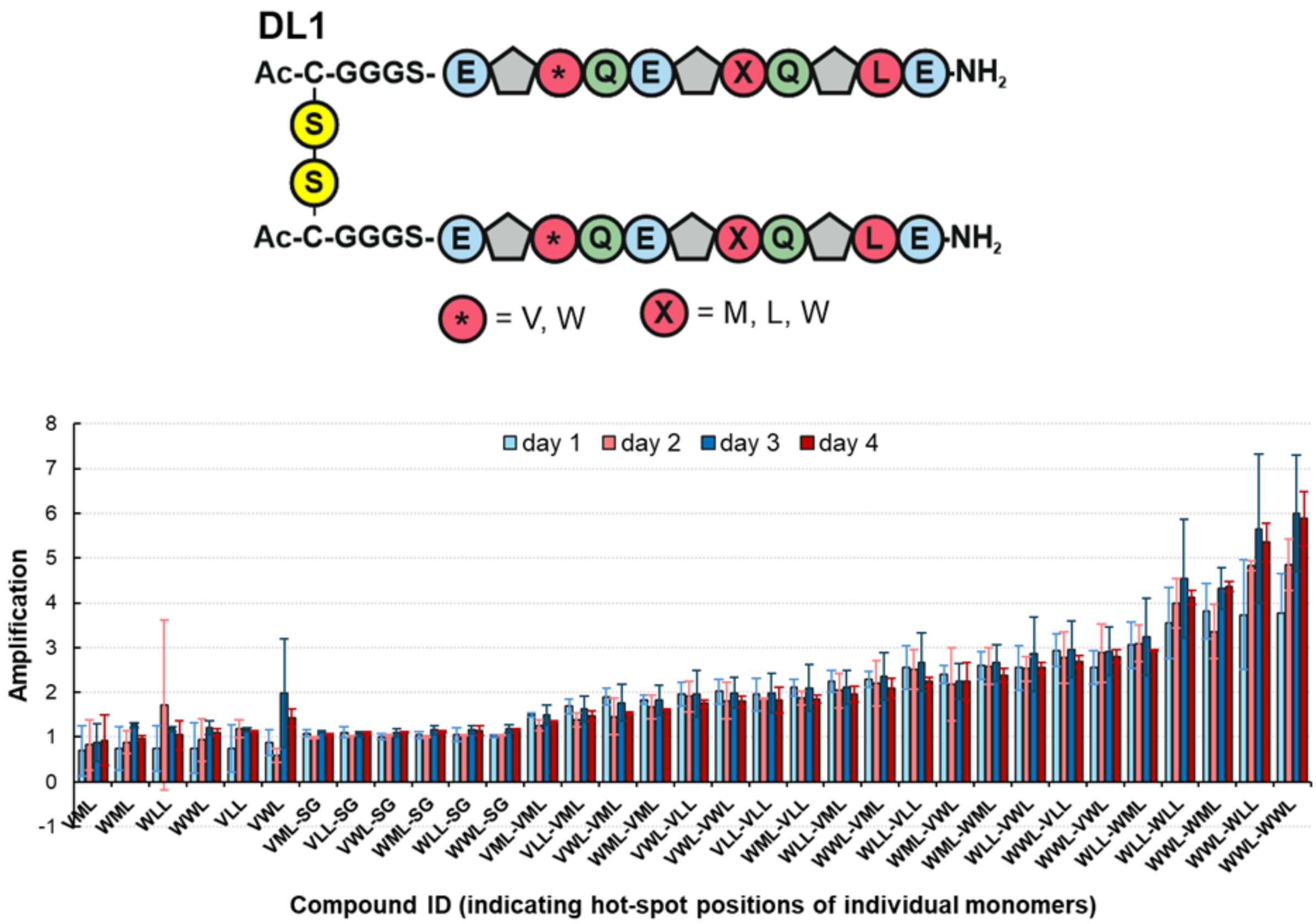

Fig. S11 **Structure of the disulfide dimers and results of the DCL experiment.**
6 monomers from L1 containing a Cys residue at the N terminus at 10 µM individual concentration was equilibrated in a glutathione red-ox buffer (50mM Na-phosphate, 50 mM NaCl at pH 7, 125 µM oxidized, 500 µM reduced glutathione, 0.02 % NaN_3) in the presence (templated) and absence (non-templated) of 5 µM KIX, at 37 °C. Equilibrium mixtures were analysed using LC-MS for 4 days. Amplification factors were calculated using: $AUC_{templated}/AUC_{non-templated}$, where AUC is the LCMS peak area integration result of an individual compound. Compound IDs represent the amino acid in the hot-spot positions, -SG indicate glutathione adducts. Error bars represent the standard deviation of amplification factors calculated from duplicate experiments.

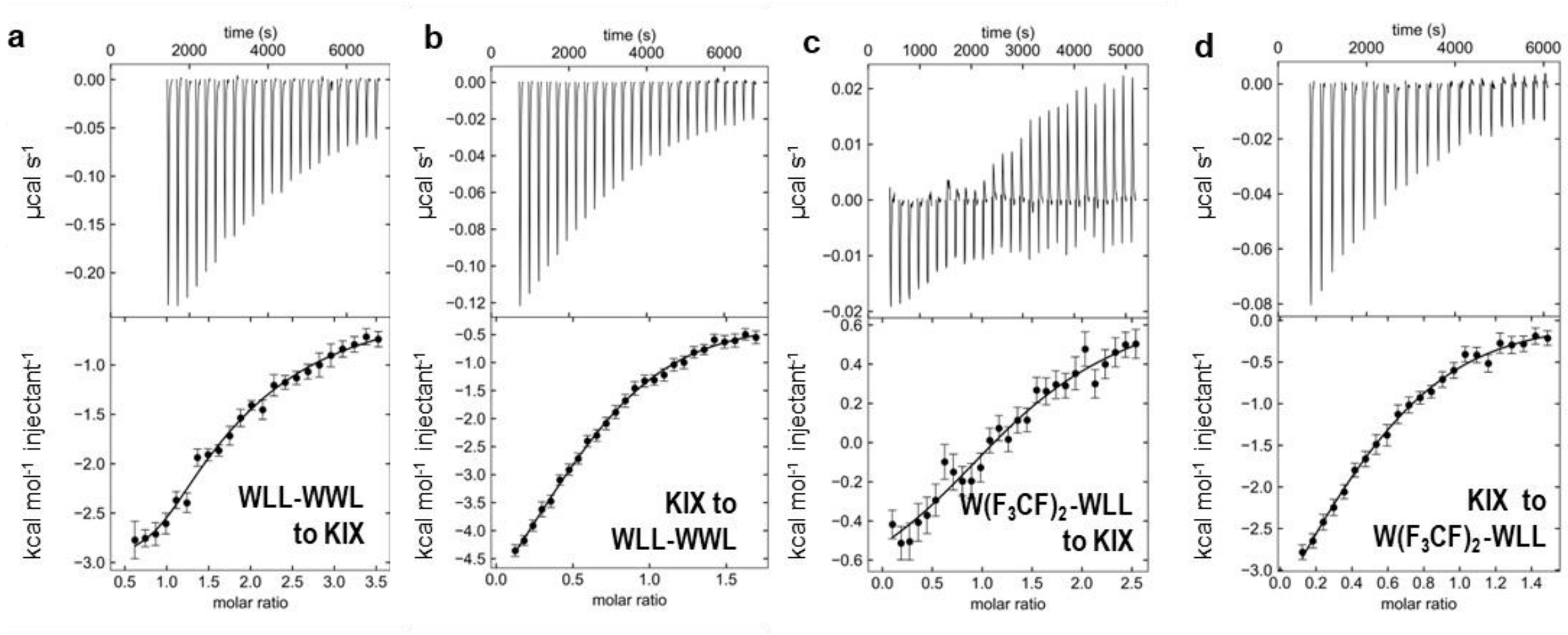

Fig. S12 Raw ITC data and fitted thermogram for direct binding of dimeric ligands to KIX
a) **WLL-WWL** dimer to CBP KIX and b) reverse titration of KIX to **WLL-WWL** dimer. c) **W(F$_3$CF)$_2$-WLL** titration to KIX and d) reverse titration of KIX to **W(F$_3$CF)$_2$-WLL** dimer. Titrations were performed at 25°C, in 50 mM Na-phosphate, 50 mM NaCl, 1 mM DTT, pH 7 buffer. Heats per injection were obtained by NITPIC integration; error bars represent NITPIC-estimated integration errors for each injection.

2 Materials and Methods

2.1 Protein expression

All incubation steps were carried out in an Excella E24R (New Brunswick) incubator shaker at 160 rpm. Centrifugation steps were carried out at 4°C using an Avanti JXN-26 centrifuge equipped with JLA 8.1 or JLA 25.50 rotor. Sonication was performed using Qsonica Q500 Sonicator equipped with a ½" probe. Size exclusion chromatography was carried out on an ÄKTApurifier (GE Healthcare) system controlled by Unicorn software. Proteins were concentrated using Amicon Ultra-15 10K concentrators at 4°C using a Hettich 320R centrifuge equipped with a swing-out rotor. The Antibiotic concentrations used for protein expression were as follows: chloramphenicol: 50 µg/ml carbenicillin: 100 µg/ml.
The DNA sequence of human CBP (UniProtKB - Q92793) containing the KIX (KID (Kinase Inducable Domain) Interacting Domain) residues 587-673 was cloned into pGEX-6P-2 plasmids using BamHI/XhoI restriction sites. BL21 E.Coli pLysS transformed with pGEX-6P-2-KIX were grown at 37 °C to OD600 0.6-0.8, induced with 0.1 mM IPTG and incubated overnight at 18 °C. Cells were harvested, lysed by sonication, and centrifuged at 25.000 g for 15 minutes at 4 °C. The supernatant was applied to glutathione beads (Glutathione Sepharose 4B, GE Healthcare) and washed with buffers containing 50 mM Tris, pH 8, 150 mM NaCl, 1 mM DTT, and 50 mM Tris, pH 8, 500 mM NaCl, 1 mM DTT. GST-KIX was eluted using 20 mM glutathione in a 50 mM Tris, 150 mM NaCl, 1 mM DTT, pH 8 buffer. GST was cleaved using Prescission protease[1] at room temperature for 2-4 hours. Cleavage was followed by SDS-PAGE. The cleaved protein was concentrated and purified by size-exclusion chromatography on S75 26/60 pg column in 50 mM Na-phosphate, 50 mM NaCl, 1 mM DTT, pH 7 buffer. Pure protein was analyzed by SDS-PAGE and mass spectrometry. GST-tagged protein was prepared similarly, without the protease cleavage step. Concentration was determined by UV-VIS spectroscopy using Nanodrop One (Thermo Scientific) using the extinction coefficient at 280 nm: 12950 M^{-1} cm^{-1} for KIX, 43110 M^{-1} cm^{-1} for GST and 55810 M^{-1} cm^{-1} for GST-KIX, as determined by Expasy ProtParam.

2.2 Peptide synthesis

Peptides were synthesized at 0.05 or 0.1 mmol scale on Tentagel S RAM resin (resin loading 0.24 mmol/g, Rapp Polimere) using CEM Liberty Blue synthesizer. Couplings were performed using 5 equivalent amino acid excesses for α-amino acids, and 3 equivalent excesses for β-amino acids. Coupling reagents were DIC (Diisopropylcarbodiimide, Fluorochem) and Oxyma (Ethyl cyano(hydroxyimino)acetate, Fluorochem) dissolved in DMF (VWR). The deprotection solution contained 10% (w/v) piperazine (Molar Chemicals) dissolved in 10% ethanol/NMP mixture. The amount of the necessary reagents was calculated using the built-in reagent calculator of Liberty Blue. All amino acids were coupled using high swelling (HS) standard Liberty Blue methods, using double couplings for α-amino acids at 0.05 mmol scale with the following settings. Step 1: Deprotection: 3mL, Microwave method: Standard Deprotection (Bubble: 2s on, 3s off; 75°C 175 W 15s, 90°C 30W 50s); Step 2-5: Wash: 4 mL; Step 6-7: Coupling: Amino acid 1.25 mL, Activator 1 mL, Activator Base 0.5 mL, manifold wash volume 2 mL, microwave method: Standard coupling (Bubble 2s on, 3s off; 75 °C 183 W 15s; 90°C 30W 110s); Step 8-9: Wash: 4 mL [4]. For β-amino acids, the following modified cycle (0.05 mmol scale) was used: Step 1: Standard Deprotection: 3mL, Bubble 2s on, 3s off; 75 °C 160W 15s 2°C ΔT; 90°C 30W 50s

---

[1]pET-NT*-HRV3CP was a gift from Gottfried Otting (Addgene plasmid # 162795; http://n2t.net/addgene:162795 ; RRID:Addgene_162795)[3]

1°C ΔT); Step 2-5: Wash: 4mL, Step 6: Coupling: Amino acid 1.25 mL, Activator 1 mL, Activator base 0.5 mL, manifold wash volume 2 mL, Microwave method: 90°C 4min Coupling (Bubbles 2s on, 3s off; 75°C 160W 15s 2°C ΔT; 90°C 30W, 230 s, 1°C ΔT).

Coupling in the variable positions for the libraries was performed using amino acid mixtures at 0.8 equivalent of total amino acid concentration relative to the synthesis scale, using modified double coupling methods (Step 1: Standard Deprotection: 3mL, Bubble 2s on, 3s off; 75 °C 160W 15s 2°C ΔT; 90°C 30W 50s 1°C ΔT); Step 2-5: Wash: 4mL, Step 6-7: Coupling: Amino acid 1.25 mL, Activator 1 mL, Activator base 0.5 mL, manifold wash volume 2 mL, Microwave method: 90°C 4min Coupling (Bubbles 2s on, 3s off; 75°C 160W 15s 2°C ΔT; 90°C 30W, 230 s, 1°C ΔT; Step 8-9: Wash: 4mL). Libraries were synthesized at 0.05 mmol scale, using 0.04 mmol amino acids (total concentration of 0.032 M) at the mixture positions, individual amino acid concentrations are listed in the table below.

Library	Amino acids in mixture positions	Concentration of individual amino acids
L1, L2, L6	F, L, M, V, W	0.0064 M
L3	D, E	0.016 M
L4	N, Q, S, T	0.008 M
L5	ACPC, β3Ala	0.016 M
L7	I, 4FF, F3CF, Pal, Thi, Tic	0.0053 M
L8	L, M, W	0.01067 M

2.2.1 Acetylation/Chloroacetylation

All peptides were acetylated using 10 equivalents of acetic anhydride and DIPEA in DCM:DMF 1:1 in a fritted SPE tube for 20 minutes 2 times at room temperature. For peptides having a chloroacetyl moiety at the N terminus, chloroacetic anhydride was coupled using 5 equivalents with DIPEA in DMF for 3 hours.

2.2.2 Fluorescent labelling

Fmoc-aminohexane carboxylic acid (Ahx) was coupled manually to the N terminus of the peptide using 5 equivalent amino acid excess, HATU as the activator, and DIPEA as the base for 3 hours at room temperature. Fmoc deprotection was carried out using 2% DBU, 2% piperazine solution in DMF for 5 and then 15 minutes. The resin was washed with DMF and DCM. 5(6)-carboxyfluorescein (Flu) was coupled using 3 equivalent excess, HATU, and DIPEA as coupling reagents overnight at room temperature. The resin was washed with the deprotection solution before cleavage to remove non-specific carboxyfluorescein esters.

2.2.3 Cleavage

Peptides were cleaved using TFA:DTT:TIS:H_2O mixture (90:2.5:2.5:5) for 3 hours, after which TFA was evaporated, and the crude peptide was precipitated in ice-cold diethyl-ether. For peptides containing chloracetyl moiety cleavage mixture was TFA:TIS:phenol:H_2O (90:2.5:5:2.5) The precipitate was washed with ice-cold diethylether, then redissolved in ACN:H_2O:AcOH mixture and lyophilized.

2.2.4 Peptide purification

Preparative RP-HPLC was carried out on a JASCO PU-4180 system equipped with a diode array detector (MD-4015) and an automatic fraction collector (Advantec, CHF122SC). Fraction collection was

monitored and programmed using ChromNAV software. Peptides were dissolved in acetonitrile/water mixture and purified on a Reprospher C18 100 A (250 x 10 mm or 250 x 20 mm) column using appropriate gradients. The purity of the peptides was characterized by HPLC-UV and HPLC-MS measurements using Dionex Ultimate 3000 HPLC system interfaced with an LTQ XL (Thermo Scientific) ion trap mass spectrometer. UV-HPLC measurements were carried out on an Agilent 1260 Infinity II system. For analytical measurements Aeris Widepore C4 (5 mm, 500 Å, 250 x 4.6 mm) or Kinetex C18 (5 mm, 100 Å, 250 x 4.6 mm) column were used, with the following gradients: 0 min 5% B, 25 min 100% B, 1 ml/min flow rate or 0 min 20% B, 25 min 100% B, 1 ml/min flow rate. The following eluents were used for HPLC-UV methods: A: 0.1% TFA/H_2O; B: 0.1% TFA/ACN.

2.2.5 Thioether conjugation

To construct dimers using thioeter linkage, the monomer containing a chloroacetyl moiety at the N terminus was mixed with the monomer (or monomers for library construction) having cysteine residue at the N terminus at 1 mM concentration 1:1.5 molar ratio in 100 mM Na-phosphate pH7-7.4 buffer and was incubated under argon atmosphere overnight, at room temperature. The conjugate was purified as described above.

2.3 LC-MS characterization and peak area integration of peptide libraries

The LC-MS characterization of peptide libraries was carried out sing Dionex Ultimate 3000 HPLC system interfaced with an LTQ XL (Thermo Scientific) ion trap mass spectrometer using a Phenomenex Aeris C4 250x4.6mm analytical column. Eluents were: A: 0.1% HCOOH/H_2O; B: 0.1% HCOOH/ACN. Gradients for each library were optimized to ensure appropriate resolution. Mass spectra were recorded in positive ionization mode.

Compounds were identified based on $[M+H]^+$ and $[M+2H]^{2+}$ ions. For compounds with the same molecular weight LC-MS/MS spectra were recorded, using CID method with an activation energy of 35, and compounds were identified based on indicative fragment ions (Table S2-11.) Peak area integrations of the highest intensity ions were carried out in Xcalibur software, with an ICIS peak identification algorithm using identified retention times, 15 smoothing points and baseline setting of 80-150. Equimolarity was estimated using the following formula $AUC_{compound}$ / (AUC_{total}/number of compounds).

2.4 Pull-down assay

The foldamer libraries were screened through pull-down assays using Glutathione Magnetic Agarose (Pierce, Thermo scientific) with a capacity of > 10 mg GST/ml. The experiments were performed in a buffer containing 50mM Na-phosphate, 50 mM NaCl and 1mM DTT at pH 7. Samples were prepared using ~35 µl settled beads, to which 100 µL 100 µM GST or GST-KIX was bound. Protein concentration was chosen to exceed the total peptide concentration of the libraries to avoid competition between ligands. For one library 150µl ~50% slurry (70µl settled resin) was pipetted into protein lobind eppendorfs, and was washed 3 times with the buffer. The magnetic beads were separated with a centrifuge and the magnetic rack. 200 µl of GST or GST-KIX was bound at 100µM concentration, and the mixture was left to rotate for 10 minutes. Protein binding was confirmed using Nanodrop from the supernatant. The protein-bound resin was washed 3 times with 500 µl buffer. Afterwards, 150 µl buffer was added to the dry resin and after vortexing and divided into two 70 µl samples (~35 µl settled resin) The foldamer libraries were added to the protein bound samples, at 100 µl volumes and 1 µM concentrations per library member. The samples were incubated at room temperature with shaking for 10 minutes. Samples were prepared in duplicates.

Samples were processed in Xcalibur Quan Browser with the prepared processing methods. Peak area integration was carried out using ICIS peak detection algorithm with 2 Da mass range for the $[M+H]^+$ ion, 15 smoothing points, 80-150 baseline, using expected retention times (Tables S2-11.) Integrations were corrected manually where it was necessary. Bound % have been calculated based on LC-MS peak area integration (AUC) using the following formula: $(AUC_{GST}-AUC_{KIX}) / AUC_{GST} *100$. For libraries that contained the initial hit **L1-WLL**, relative values were calculated using: $Bound\%_{compound} / Bound\%_{L1-WLL}$

2.5 Isothermal titration calorimetry

The ITC measurements were performed with the following buffers: 50 mM Na-phosphate, 50 mM NaCl, 1 mM DTT, pH 7. Experiments were carried out using a Microcal VP ITC instrument, with 20 µM protein in the cell and 250 µM ligand in the syringe at 25 °C, using 10 µl injections and 180s or 240s spacing between injections, for 28 injections. Reverse titrations were carried out using 10 µM ligand in the cell and 80 µM KIX in the syringe using 10 µl injections, 240 s spacing. Raw data integration was performed through SVD analysis implemented in NITPIC[5] and fitted using SEDPHAT.[6] For MLL and MYB titrations a single binding site model was used with a fixed 1:1 stoichiometry (A + B <-> AB Hetero association model in SEDPHAT), with log*K*a, Δ*H*, baseline and incompetent protein fraction as adjustable parameters. For all other titrations a non-symmetric binding, protein:ligand 1:2 model was used (A+B+B <-> AB + B <-> BA + B <-> BAB; with 2 non-symmetric sites, microscopic K model in SEDPHAT) with $\log K_{AB}$, ΔH_{AB}, $\log(K_{BA}/K_{AB})$, $\Delta H_{BA}/\Delta H_{AB}$, baseline and incompetent protein fraction as adjustable parameters. Forward and reverse titrations (where available) were fitted together, globally to the same model. Fitting error was determined using the automatic confidence interval search implemented in SEDPHAT (p=0.683). Figures were prepared by GUSSI.[7]

2.6 Fluorescence anisotropy

Fluorescence anisotropy was measured using a Clariostar Plus microplate reader in a 384-well black plate with excitation at 493 nM and emission at 517 nm at 25°C. The buffer used for the experiments was 50 mM Na-phosphate, 50 mM NaCl, 0.01% Triton-X, pH 7. Three repeats were performed for each titration, and in parallel, a control experiment was prepared where the fluorescently labelled CFU-linked peptide was replaced with buffer in the reaction mixture, which was subtracted from the raw data. The competitor peptides were diluted by mixing 20 µL peptide solution with 20 µL buffer in the first well and then serially diluted over 12 points on the plate (1/2 dilution series) resulting in 100 µM as the highest concentration in the first well. To each well, KIX at a final concentration of 2 µM and tracer CFU-MLL or CFU-MYB at a final concentration of 50 nM was added.
Fluorescence intensity and anisotropy were calculated according to the following equations as previously described [4].

$$I = (2PG)+S \quad Eq.\ 1.$$

$$r = (S-PG) \quad Eq.\ 2.$$

r = anisotropy, I = total intensity, P = perpendicular intensity, S = parallel intensity, G = an instrument factor set to 1. Anisotropy values were normalized to fraction bound using:

$$(r- r_{min})/(r_{max} -r)+ r- r_{min} \quad Eq.\ 3$$

The calculated average anisotropy values and their standard deviation were then fitted to a sigmoidal logistic model (Equation 4) using OriginPro, which provided the IC50 and error values.

$$y = r_{max} + (r_{min}-r_{max})/(1+(x/x_o)^p) \quad Eq.\ 4$$

2.7 Native MS measurements

For native MS measurements KIX was dialysed into a 10 µM NH_4Ac pH7 buffer. Samples were prepared using 3 µM KIX mixed with 9 µM **L1-WLL** and infused directly to the mass spectrometer (Thermo LTQ XL) using a 500 µL syringe at 10 µL/min flow rate with the following ESI source settings: Source voltage: 2 kV; Source temperature 65 °C; Capillary temperature 200 °C; Sheath gas flow rate: 20; Aux gas flow rate: 10; Capillary voltage: 10 V; Tube lens voltage: 86 V. An average of 80 scans were taken in positive ion mode, from 400-2000 m/z value. Mass spectra were deconvoluted using UNIDEC[2].

2.8 NMR measurements

2.8.1 Secondary structure determination

NMR measurements were performed on a Bruker Avance III 600 MHz spectrometer equipped with a 5 mm CP-TCI triple-resonance cryoprobe. **L1-WLL** was dissolved at 1mM concentration in 50 mM Na-phosphate, 50 mM NaCl, pH 7 buffer. Measurements were carried out at 298K. 2D-TOCSY and ROESY spectra were recorded using excitation sculpting solvent suppression pulse scheme implemented in Topspin 4.1 (dipsi2esgpph and roesyesgpph pulse programs). TOCSY mixing time was 80 ms, ROESY mixing time 250 ms.

2.8.2 Binding site determination

Samples were prepared in 50 mM Na-phosphate, 25 mM NaCl, 10% D_2O, 0.02% NaN_3 buffer using ^{15}N-^{13}C-KIX at 210 µM concentration, to which 2 equivalents of **L7-W(F_3CF)$_2$** were added. NMR experiments were carried out on a Bruker Avance III 600 MHz spectrometer equipped with a 5 mm CP-TCI triple-resonance cryoprobe. Measurements were carried out at 303 K. For signal assignments the following spectra were recorded: ^1H-^{15}N-HSQC, HNCO, HNCA, HN(CO)CA, CBCA(CO)NH using pulse schemes implemented in Topspin 4.1 (Bruker), with excitation sculpting water suppression. Processing was carried out using Topspin 4.1, and data were analyzed using NMRFAM-Sparky[8]. Chemical shift perturbation (CSP) was calculated using $\boldsymbol{CSP} = \sqrt{\left[\boldsymbol{\Delta\delta_H^2} + \mathbf{0.14}\boldsymbol{\Delta\delta_N^2}\right]}$.

Significance level was determined at $CSP > 0.9\ CSP_{average} + \sigma$.

2.9 Dynamic covalent chemistry

Dynamic covalent chemistry experiments were performed with foldamers containing free SH groups. The reactions contained 10 µM of each library member and 5 µM of KIX in a buffer made of 50 mM NaP, 50mM NaCl, 3mM NaN_3, 125 µM reduced glutathione and 500 µM oxidised glutathione. A control sample was prepared similarly, without the template protein. The mixture was incubated at 37°C and samples were taken every 24 hours. Disulphide exchange was quenched by adding 100 µl 10% TFA to the 100 µl sample. The contents of the sample were measured with HPLC MS.

3 Library characterization

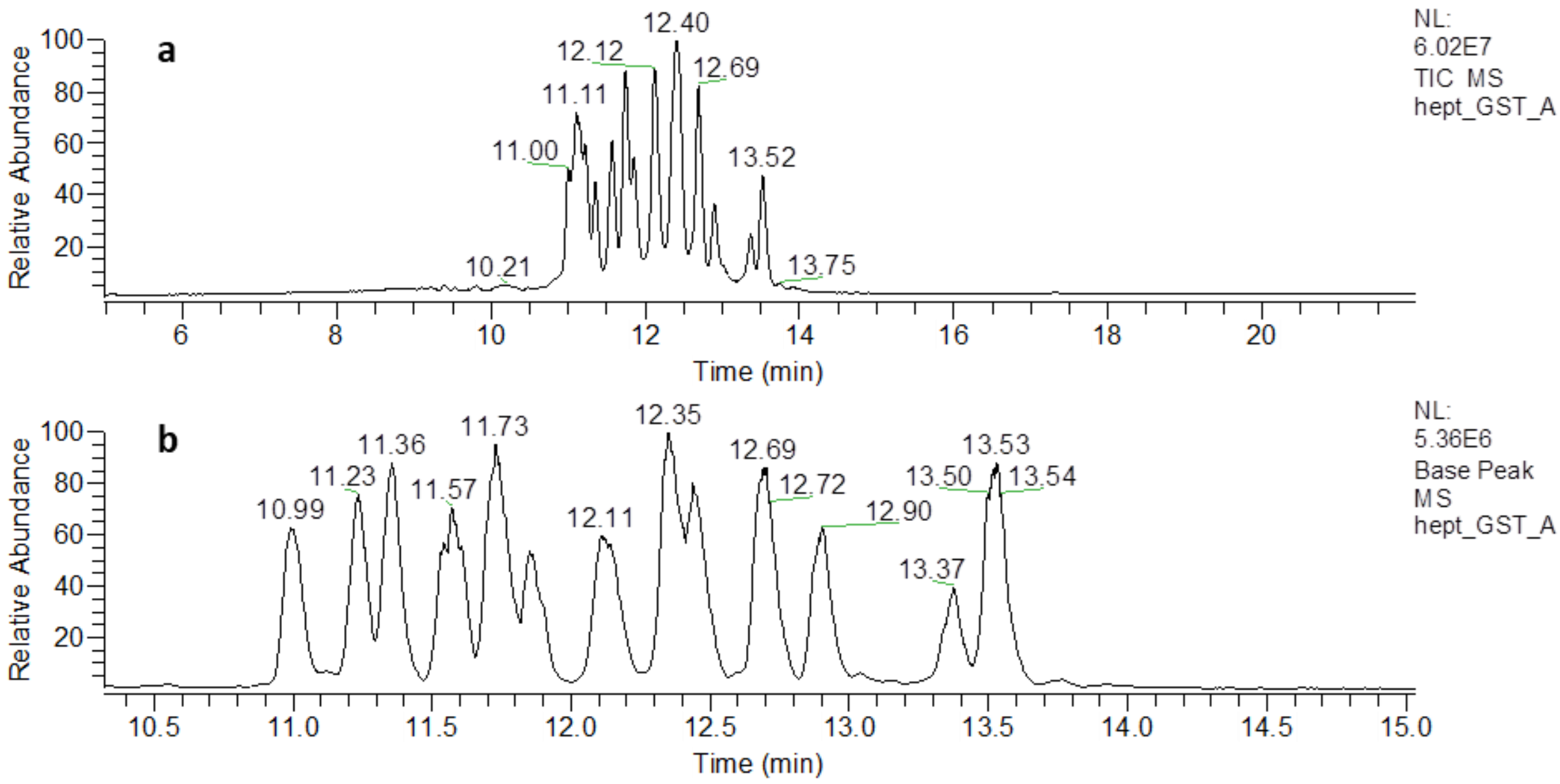

Fig. S13 LCMS chromatograms of **L1**. a) Total ion chromatogram, b) Base-peak chromatogram of compounds in the relevant retention time window. Estimated purity: 85% (Purity estimation was based on integration of total ion chromatogram of the compounds in the relevant retention time window, relative to total peak area of the entire chromatogram.)

Table S2. LC-MS characterization of Library 1 (**L1**), with the following amino acid pattern: αβαααβααβαα including 2 variable hot-spot positions. The listed MS^2 ions were used to identify peptides with the same molecular mass.

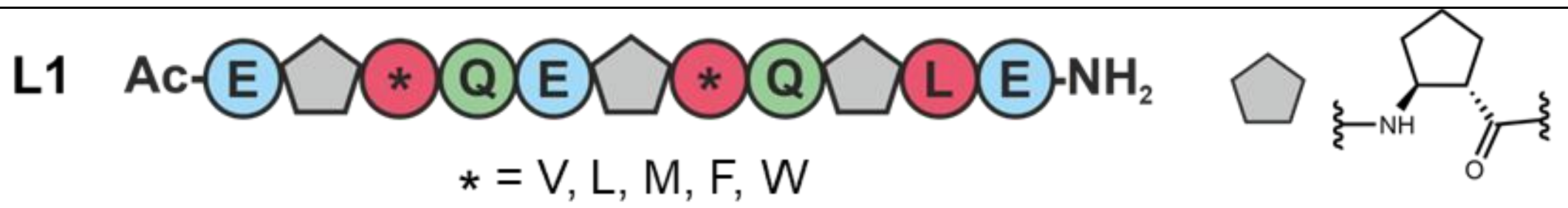

**Amino acid at position:**		**Molecular weight**	**Exact mass**	**Detected ions m/z**				**Retention time (min)[a]**
**3**	**7**			$[M+H]^+$	$[M+2H]^{2+}$	y8	$b8-NH_2$	
V	V	1347.33	1346.63	1347.39	675.15			11.37
L	V	1361.36	1360.65	1361.48	681.59	382.14	963.63	12.35
V	L	1361.36	1360.65	1361.41	682.21	396.15	949.54	12.47
L	L	1375.38	1374.66	1375.46	689.14			13.55
M	V	1379.39	1378.60	1379.40	690.59	414.24	949.46	11.01
V	M	1379.39	1378.6	1379.47	690.60	382.15	981.59	11.60
L	M	1393.42	1392.62	1393.34	698.07		963.70	11.77
M	L	1393.42	1392.62	1393.49	697.24	396.08	981.55	12.72
F	V	1395.37	1394.63	1395.55	699.08	430.26	949.71	11.88
V	F	1395.37	1394.63	1395.55	699.08	382.20		12.18
F	L	1409.40	1408.65	1409.45	705.35	430.16	963.62	12.72
L	F	1409.40	1408.65	1409.43	705.39	396.19	997.58	13.40
M	M	1411.46	1410.58	1411.31	706.28			11.25
F	M	1427.44	1426.60	1427.39	714.56	430.20		11.77
M	F	1427.44	1426.60	1427.34	714.22	414.11	997.56	12.13
V	W	1434.41	1433.64	1434.29	717.90	382.24		11.75
W	V	1434.41	1433.64	1434.29	717.90	469.21	949.55	11.75
F	F	1443.41	1442.63	1443.41	722.31			12.42
L	W	1448.44	1447.66	1448.43	725.20	469.19		12.46
W	L	1448.44	1447.66	1448.36	724.97	396.18	1036.61	12.91
M	W	1466.47	1465.62	1466.38	734.07	414.31		11.56
W	M	1466.47	1465.62	1466.27	734.08	469.25	981.55	11.88
F	W	1482.45	1481.64	1482.27	741.93	469.16		12.15
W	F	1482.45	1481.64	1482.32	742.09	430.17	1036.63	12.41
W	W	1521.49	1520.65	1521.36	761.61			12.13

[a] Retention time based on measurement on Aeris Widepore C4 250 x 4.6 mm column, using a gradient of 5-40-65 % B over 25 min (0 min 5% B, 5 min 40% B, 25 min 65% B, 0.7 ml/min.)

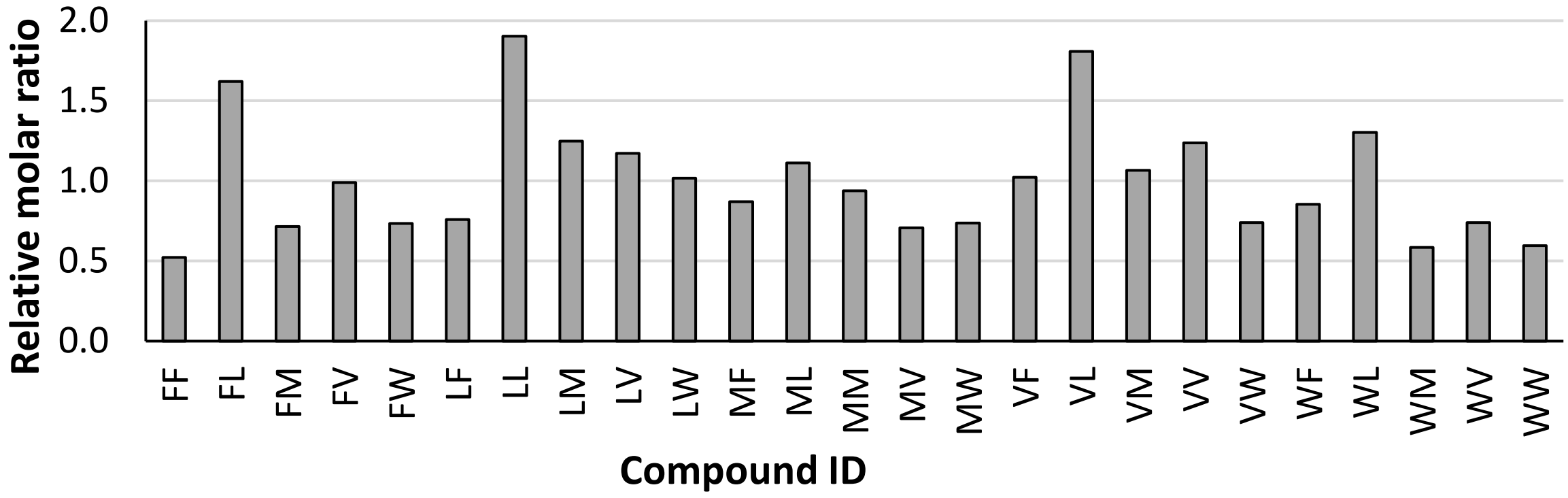

Fig. S14 Estimated equimolarity of **L1** based on LC-MS peak area integration (AUC) of selective ion chromatograms using $[M+H]^+$ charge states with 2 Da window. Compound IDs are generated using the single letter amino acid codes at the variable positions 3 and 7. Relative molar ratio is estimated using the following formula: $AUC_{compound}$ / (AUC_{total}/25). Due to insufficient separation VW and WV integrated together.

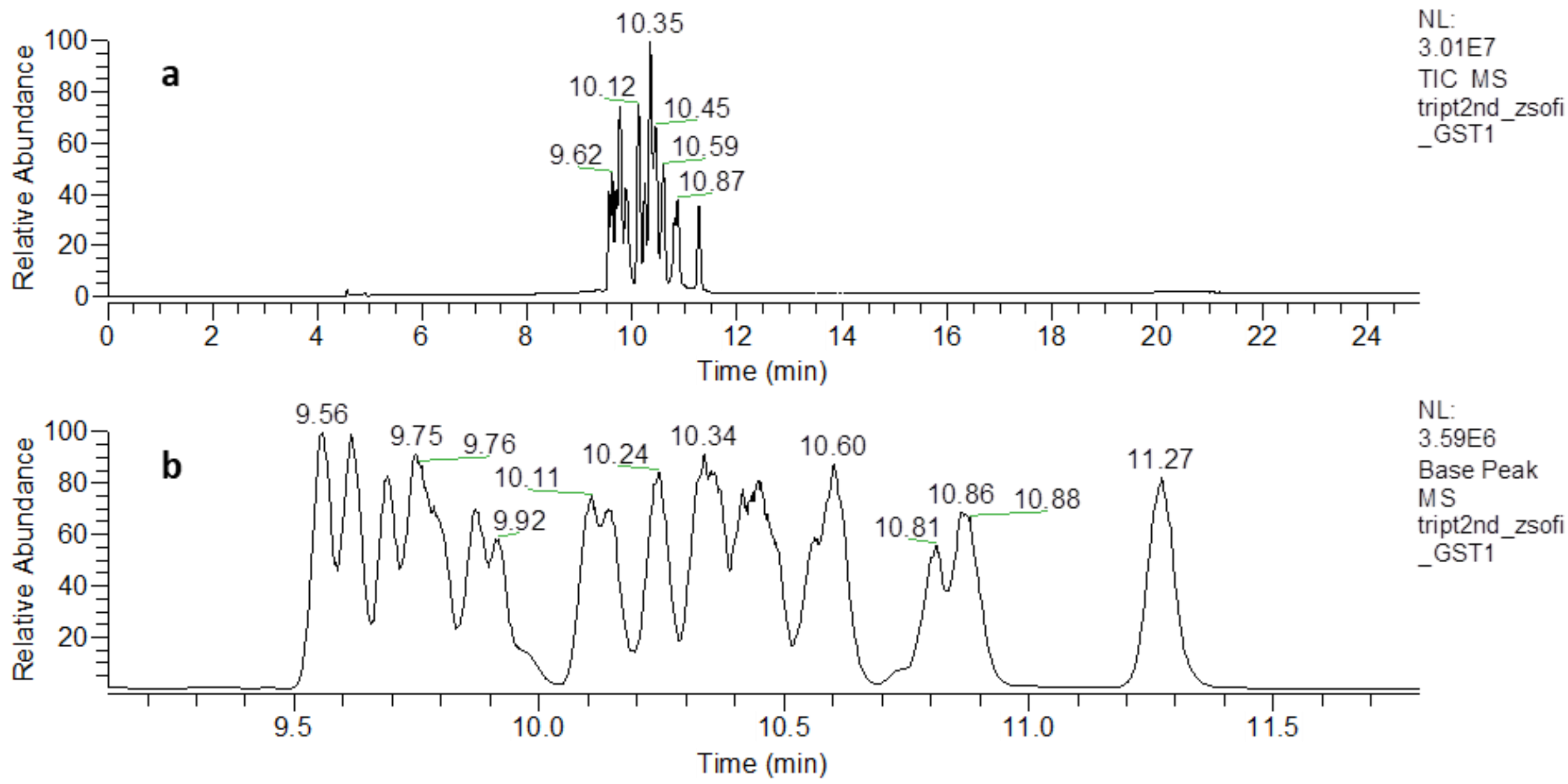

Fig. S15 LCMS chromatograms of **L2**. a) Total ion chromatogram, b) Base-peak chromatogram of compounds in the relevant retention time window. Estimated purity: 87% (Purity estimation was based on integration of total ion chromatogram of the compounds in the relevant retention time window, relative to total peak area of the entire chromatogram.)

Table S3. LC-MS characterization of Library 2 (**L2**), with the following amino acid pattern: ααβααβααβαα including 2 variable hot-spot positions. The listed MS^2 ions were used to identify peptides with the same molecular mass.

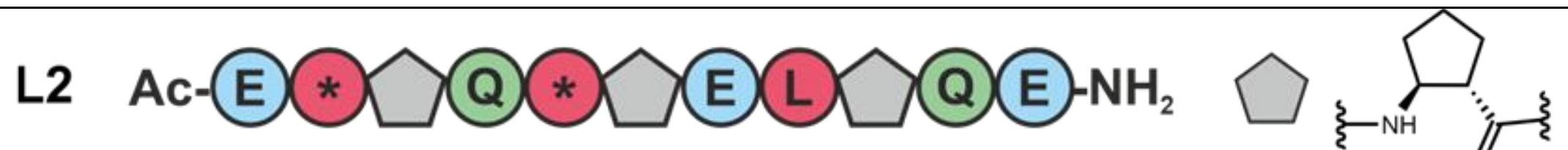

$*$ = V, L, M, F, W

Amino acid at position:		Molecular weight	Exact mass	Detected ions m/z			Retention time (min)[a]
2	5			$[M+H]^+$	$[M+2H]^{2+}$	y7	
V	V	1347.33	1346.63	1347.32	674.64		9.7
L	V	1361.36	1360.65	1361.5	681.5	821.3	10.6
V	L	1361.36	1360.65	1361.44	681.54	835.24	10.3
L	L	1375.38	1374.66	1375.4	688.7		11.3
M	V	1379.39	1378.60	1379.37	690.55	821.46	9.8
V	M	1379.39	1378.6	1379.4	690.6	853.3	9.6
L	M	1393.42	1392.62	1393.4	697.6	853.4	10.3
M	L	1393.42	1392.62	1393.4	697.5	852.4	10.1
F	V	1395.37	1394.63	1395.4	698.5	821.4	10.4
V	F	1395.37	1394.63	1395.4	698.5	868.4	9.9
F	L	1409.40	1408.65	1409.4	705.6	845.4	10.9
L	F	1409.40	1408.65	1409.4	705.5	869.4	10.5
M	M	1411.46	1410.58	1411.4	706.6		9.6
F	M	1427.44	1426.60	1427.4	714.5	853.4	10.1
M	F	1427.44	1426.60	1427.4	714.5	869.0	9.8
V	W	1434.41	1433.64	1434.41	718.1	908.27	9.9
W	V	1434.41	1433.64	1434.4	718.01	821.43	10.6
F	F	1443.41	1442.63	1443.4	722.6		10.4
L	W	1448.44	1447.66	1448.4	725.1	908.3	10.5
W	L	1448.44	1447.66	1448.47	725.04	835.48	10.8
M	W	1466.47	1465.62	1466.4	734.0	908.41	9.8
W	M	1466.47	1465.62	1466.32	734.1	853.58	10.1
F	W	1482.45	1481.64	1482.4	742.0	908.4	10.4
W	F	1482.45	1481.64	1482.36	742.01	869.28	10.4
W	W	1521.49	1520.65	1521.38	761.51		10.4

[a] Retention time based on measurement on Aeris Widepore C4 250 x 4.6 mm column, using a gradient of 5-35-45 % B over 25 min (0 min 5% B, 5 min 35% B, 25 min 45% B, 0.7 ml/min.)

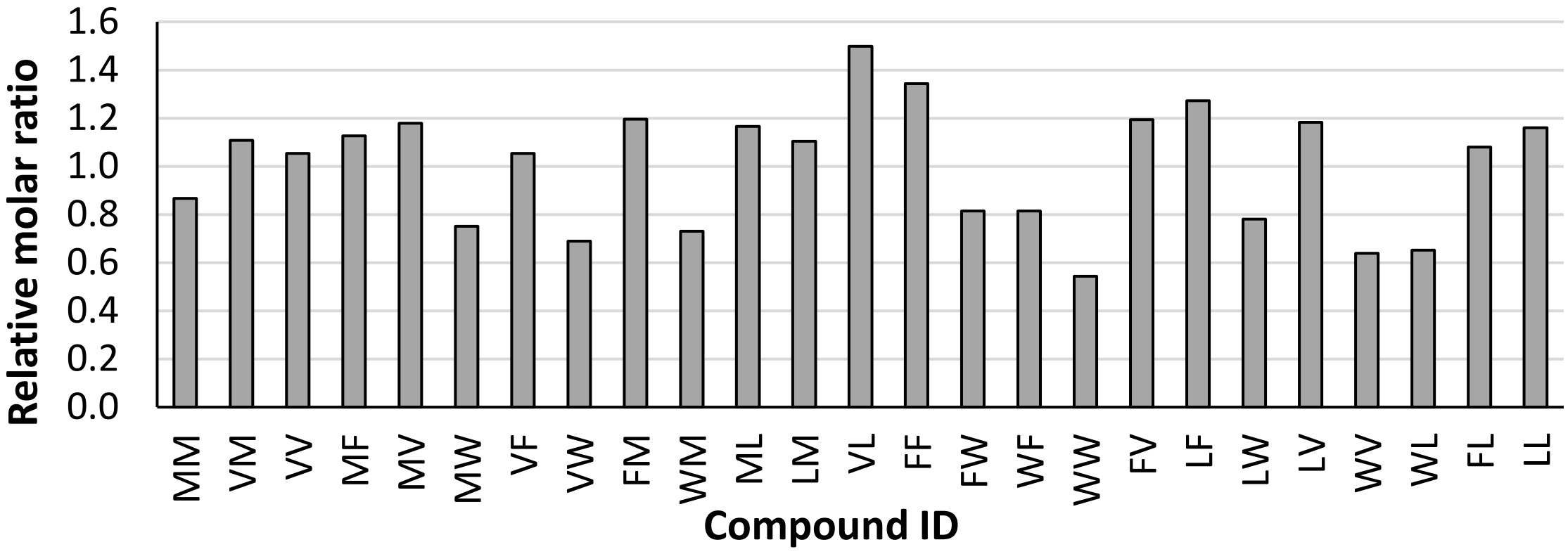

Fig. S16 Estimated equimolarity of **L2** based on LC-MS peak area integration (AUC) of selective ion chromatograms using $[M+H]^+$ charge states with 2 Da window. Compound IDs are generated using the single letter amino acid codes at the variable positions 2 and 5. Relative molar ratio is estimated using the following formula: $AUC_{compound}$ / (AUC_{total}/25).

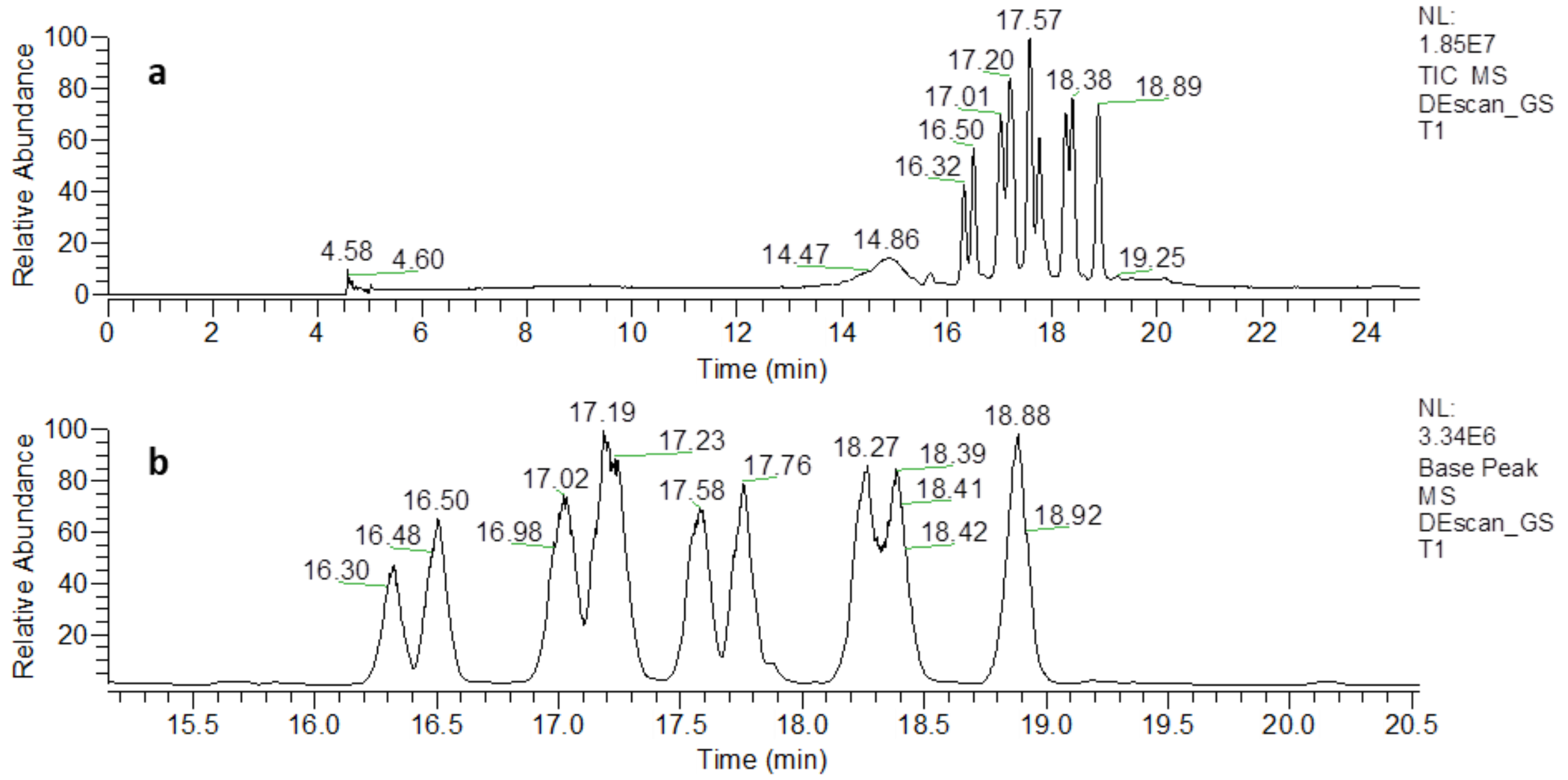

Fig. S17 LCMS chromatograms of **L3**. a) Total ion chromatogram, b) Base-peak chromatogram of compounds in the relevant retention time window. Estimated purity: 74% (Purity estimation was based on integration of total ion chromatogram of the compounds in the relevant retention time window, relative to total peak area of the entire chromatogram.)

Table S4. LC-MS characterization of Library 3 (**L3**), with the following amino acid pattern: ααβααβααβαα including 3 variable positions at the negatively charged amino acids. The listed MS^2 ions were used to identify peptides with the same molecular mass.

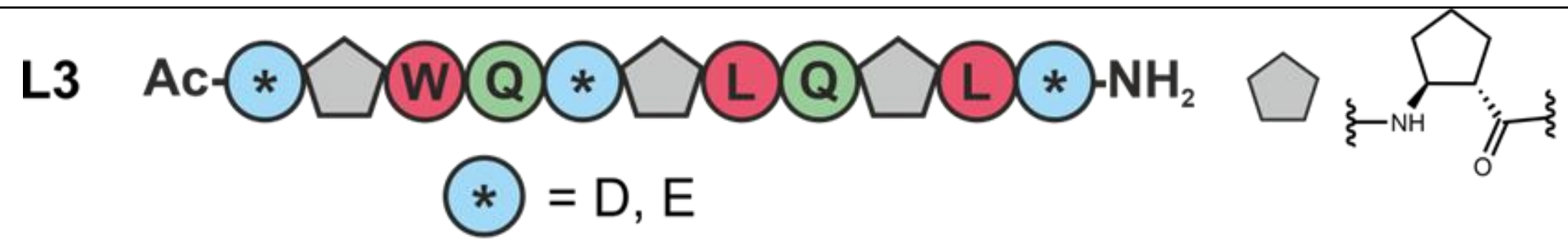

Amino acid at position:			Molecular weight	Exact mass	Detected ions m/z					Retention time (min)
1	5	11			$[M+H]^+$	$[M+2H]^{2+}$	b1	b4	b8	
D	D	D	1406.35	1405.61	1406.40	703.95				16.40
D	E	D	1420.38	1419.63	1420.40	710.97	1288.54	936.44	468.37	17.10
E	D	D	1420.38	1419.63	1420.40	710.97	1288.57	936.34	455.19	17.10
D	D	E	1420.38	1419.63	1420.40	711.03	1274.54	922.41	455.22	17.52
E	E	D	1434.41	1433.64	1434.39	718.02	1302.52	950.53	468.33	17.65
D	E	E	1434.41	1433.64	1434.41	718.01	1288.54	936.42	455.24	18.16
E	D	E	1434.41	1433.64	1434.39	718.02	1288.60	936.44	469.26	18.32
E	E	E	1448.44	1447.66	1448.42	725.04				18.74

[a] Retention time based on measurement on Aeris Widepore C4 250 x 4.6 mm column, using a gradient of 5-35-45 % B over 25 min, 0.7 ml/min.

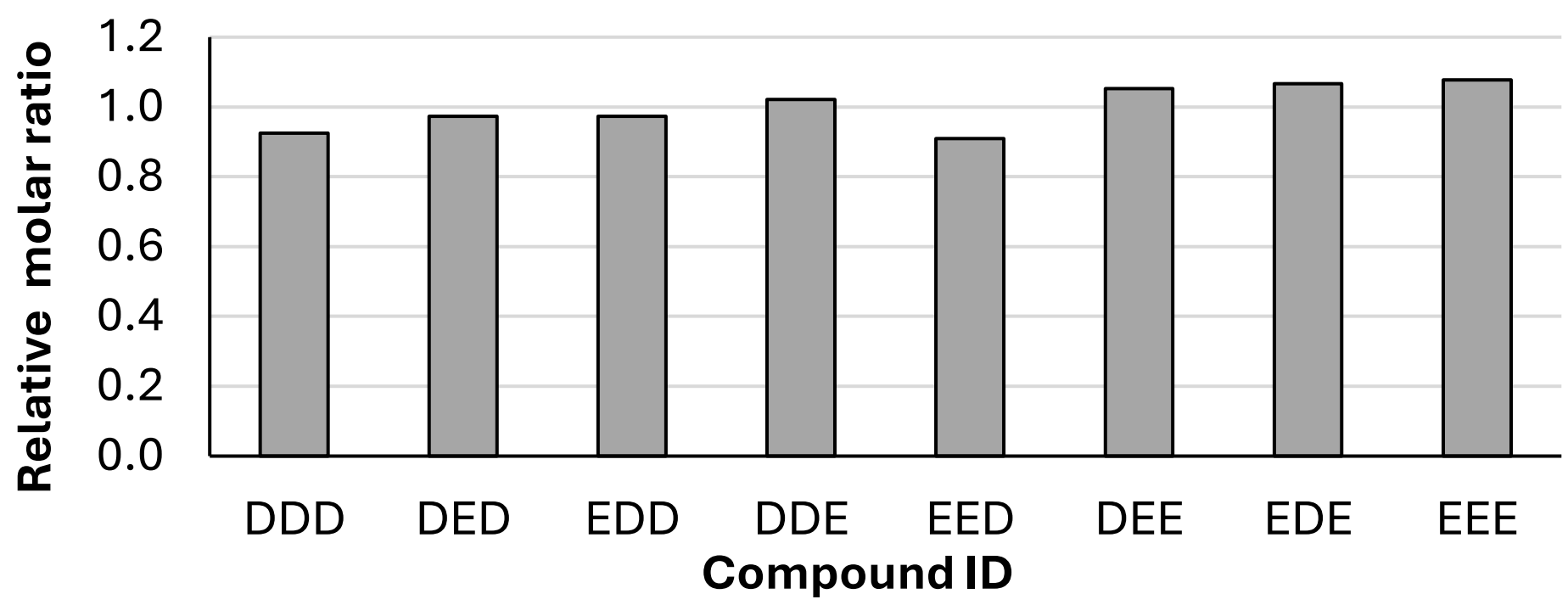

Fig. S18 Estimated equimolarity of **L3** based on LC-MS peak area integration (AUC) of selective ion chromatograms using $[M+H]^+$ charge states with 2 Da window. Compound IDs are generated using the single letter amino acid codes at the variable positions 1, 5 and 11. Relative molar ratio is estimated using the following formula: $AUC_{compound}$ / (AUC_{total}/8). Due to insufficient separation DED and EDD were integrated together.

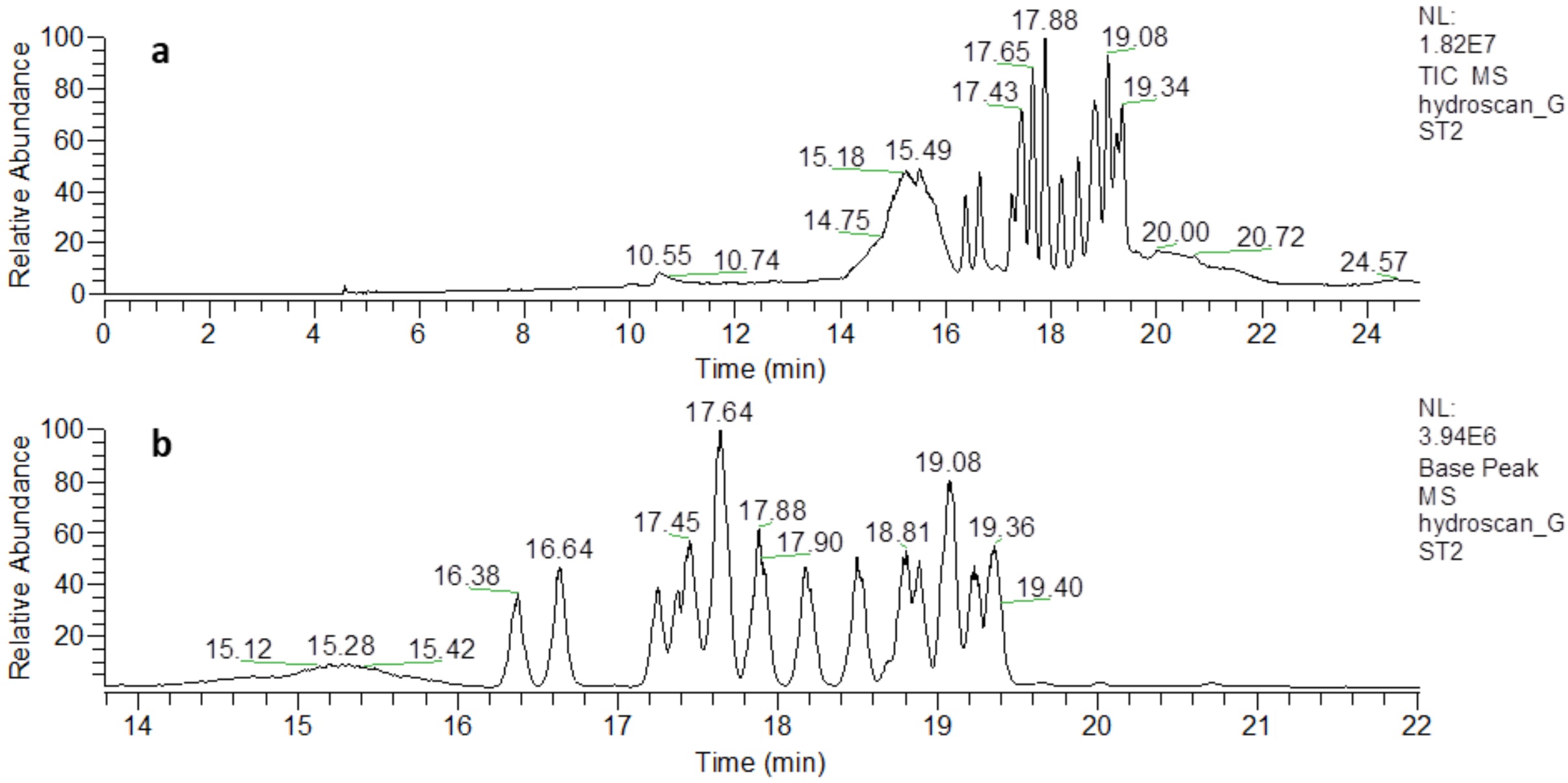

Fig. S19 LCMS chromatograms of **L4**. a) Total ion chromatogram, b) Base-peak chromatogram of compounds in the relevant retention time window. Estimated purity: 55% (Purity estimation was based on integration of total ion chromatogram of the compounds in the relevant retention time window, relative to total peak area of the entire chromatogram.)

Table S5. LC-MS characterization of Library 4 (**L4**), with the following amino acid pattern: ααβααβααβαα including 2 variable positions at the hydrophilic amino acids. The listed MS^2 ions were used to identify peptides with the same molecular mass.

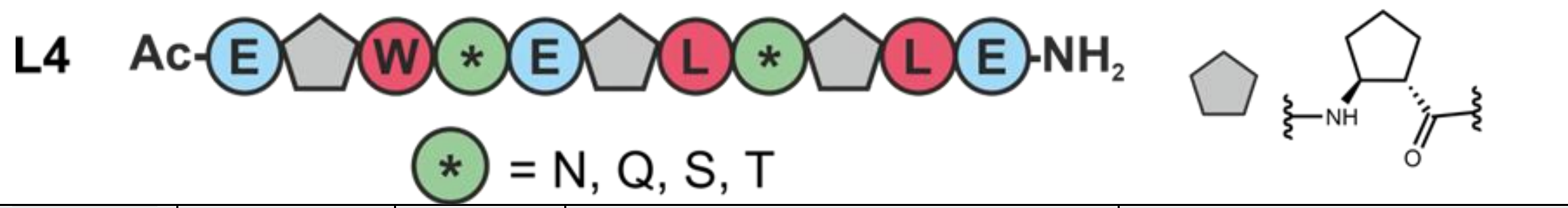

Amino acid at position:		Molecular weight	Exact mass	Detected ions m/z			Retention time (min)[a]
4	8			$[M+H]^+$	$[M+2H]^{2+}$	b4	
S	S	1366.33	1365.61	1366.47	684.09		17.8
S	T	1380.36	1379.62	1380.47	691.08	909.30	17.8
T	S	1380.36	1379.62	1380.46	691.13	923.34	18.8
S	N	1393.36	1392.62	1393.28	697.62	909.35	16.6
N	S	1393.36	1392.62	1393.47	698.05	936.32	17.4
T	T	1394.38	1393.64	1394.36	698.53		19.1
Q	S	1407.38	1406.63	1407.40	705.17	951.33	19.2
N	T	1407.38	1406.63	1407.46	704.54	936.32	17.4
S	Q	1407.38	1406.63	1407.40	704.58	909.26	17.6
T	N	1407.38	1406.63	1407.40	704.58	923.31	17.6
N	N	1420.38	1419.63	1420.46	711.24		16.3
Q	T	1421.41	1420.65	1421.44	711.86	950.24	19.3
T	Q	1421.41	1420.65	1421.50	711.22	923.24	18.4
Q	N	1434.41	1433.64	1434.43	718.05	950.38	18.1
N	Q	1434.41	1433.64	1434.35	718.56	937.40	17.2
Q	Q	1448.44	1447.66	1448.47	724.87		18.8

[a] Retention time based on measurement on Aeris Widepore C4 250 x 4.6 mm column, using a gradient of 5-35-45 % B over 25 min, 0.7 ml/min.

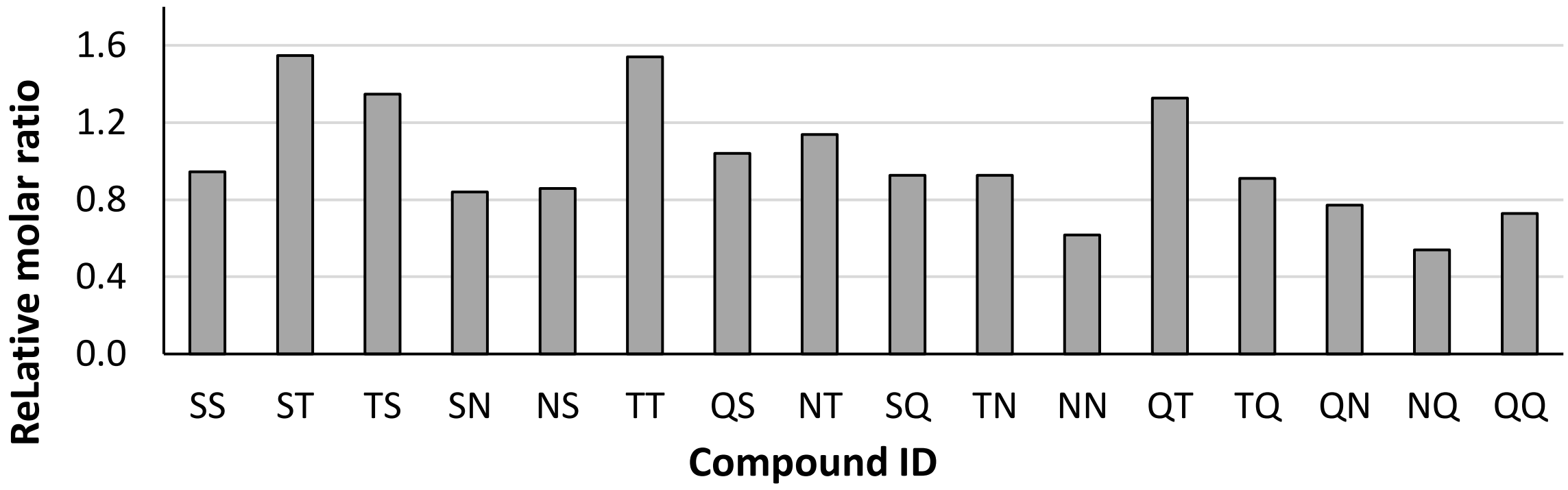

Fig. S20 Estimated equimolarity of **L4** based on LC-MS peak area integration (AUC) of selective ion chromatograms using $[M+H]^+$ charge states with 2 Da window. Compound IDs are generated using the single letter amino acid codes at the variable positions 4 and 8. Relative molar ratio is estimated using the following formula: $AUC_{compound}$ / (AUC_{total}/16). Due to insufficient separation SQ and TN were integrated together.

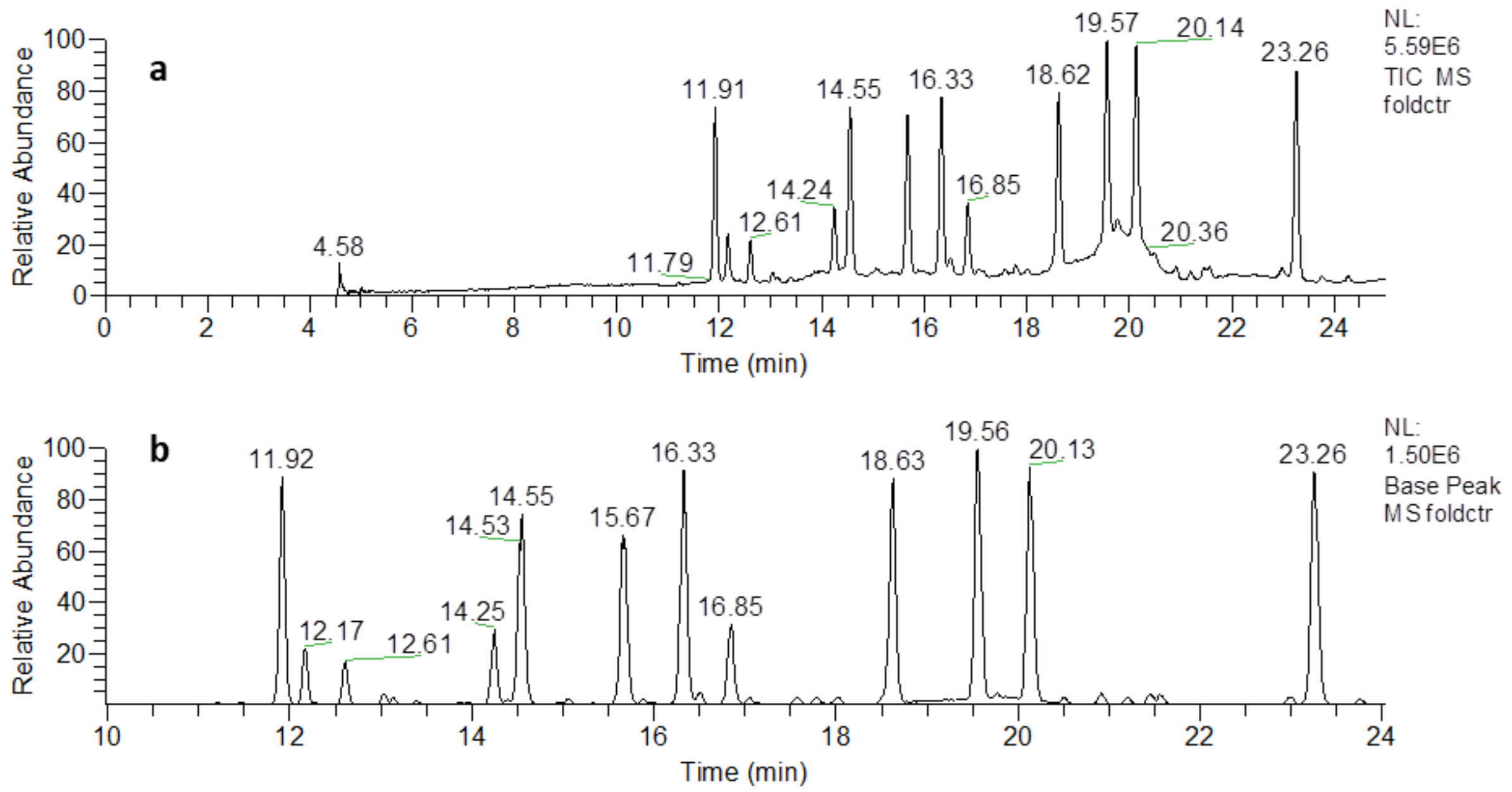

Fig. S21 LCMS chromatograms of **L5.** a) Total ion chromatogram, b) Base-peak chromatogram of compounds in the relevant retention time window. Estimated purity: 54% (Purity estimation was based on integration of total ion chromatogram of the compounds in the relevant retention time window, relative to total peak area of the entire chromatogram.)

Table S6. LC-MS characterization of Library 5 (**L5**), with the following amino acid pattern: ααβααβααβαα including 3 variable positions at the beta amino acid sites. The listed MS^2 ions were used to identify peptides with the same molecular mass.

L5 Ac-E-*-W-Q-E-*-L-Q-*-L-E-NH2; * = S,S-ACPC, β^3Ala									
**Amino acid at position:**			**Molecular weight**	**Exact mass**	**Detected ions m/z**				**Retention time (min)**[a]
**2**	**6**	**9**			$[M+H]^+$	$[M+2H]^{2+}$	b4	b8	
β^3A	β^3A	β^3A	1371.28	1370.59	1370.38	685.83			11.88
ACPC	β^3A	β^3A	1397.33	1396.61	1396.35	699.09	924.23	469.16	16.31
β^3A	ACPC	β^3A	1397.33	1396.61	1396.36	699.03	924.28	443.05	15.63
β^3A	β^3A	ACPC	1397.33	1396.61	1396.38	699.05	898.25	443.40	14.51
ACPC	β^3A	ACPC	1423.38	1422.64	1422.34	712.07	924.20	469.10	19.60
ACPC	ACPC	β^3A	1423.38	1422.64	1422.31	711.91	950.20	469.10	20.13
β^3A	ACPC	ACPC	1423.38	1422.64	1422.39	712.08	924.22	443.11	18.58
ACPC	ACPC	ACPC	1449.44	1448.66	1448.36	724.89			23.18

[a] Retention time based on measurement on Aeris Widepore C4 250 x 4.6 mm column, using a gradient of 5-30-45 % B over 25 min, 0.7 ml/min. ACPC: S,S-aminocyclopentane carboxylic acid; β^3A: beta3-homo-alanine

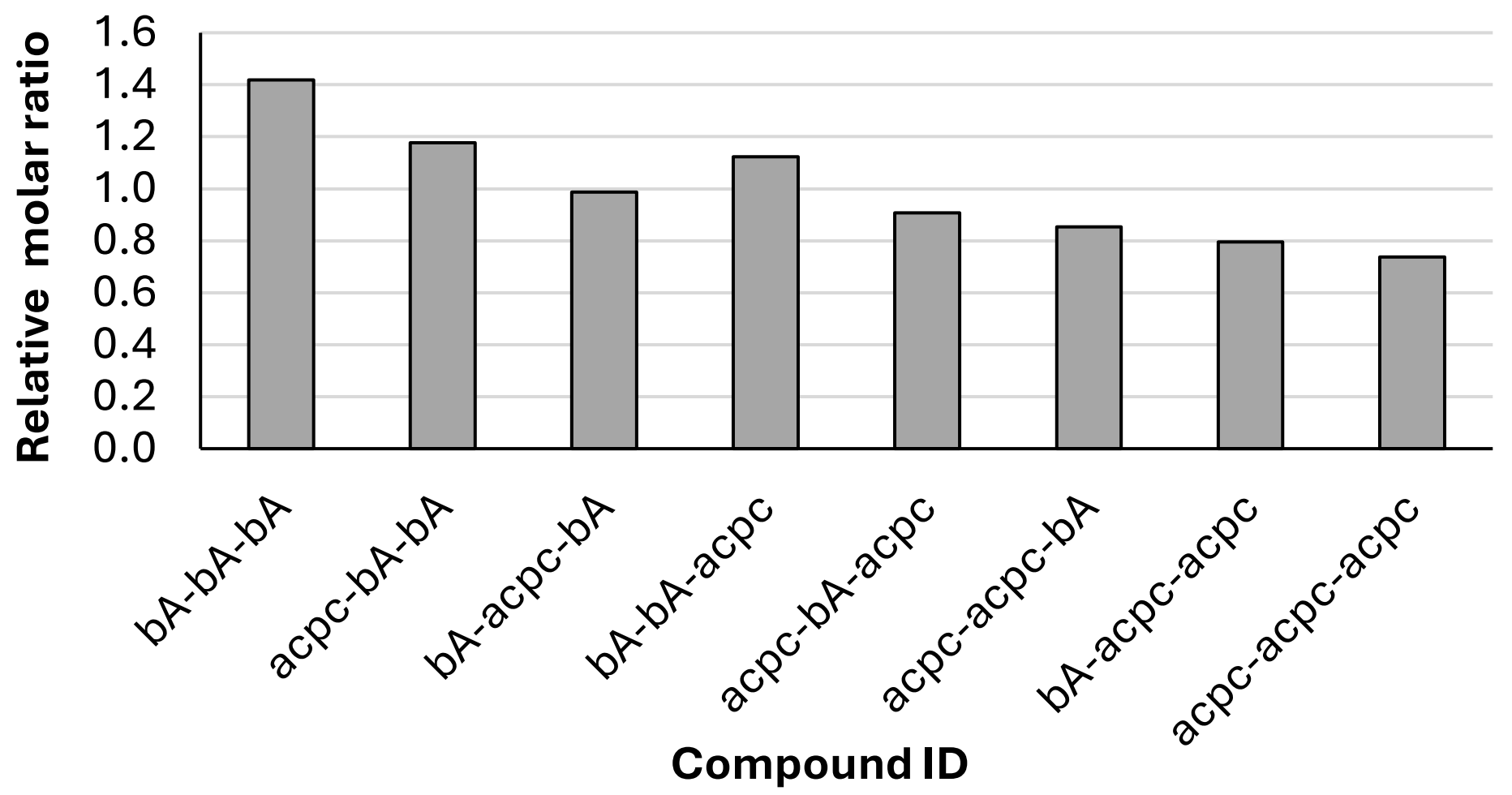

Fig. S22 Estimated equimolarity of **L5** based on LC-MS peak area integration (AUC) of selective ion chromatograms using $[M+H]^+$ charge states with 2 Da window. Compound IDs are generated using the abbreviations of the amino acids at positions 2, 6 and 9 (acpc: S,S-ACPC, bA: $beta^3$-homo-Alanine). Relative molar ratio is estimated using the following formula: $AUC_{compound}$ / (AUC_{total}/8).

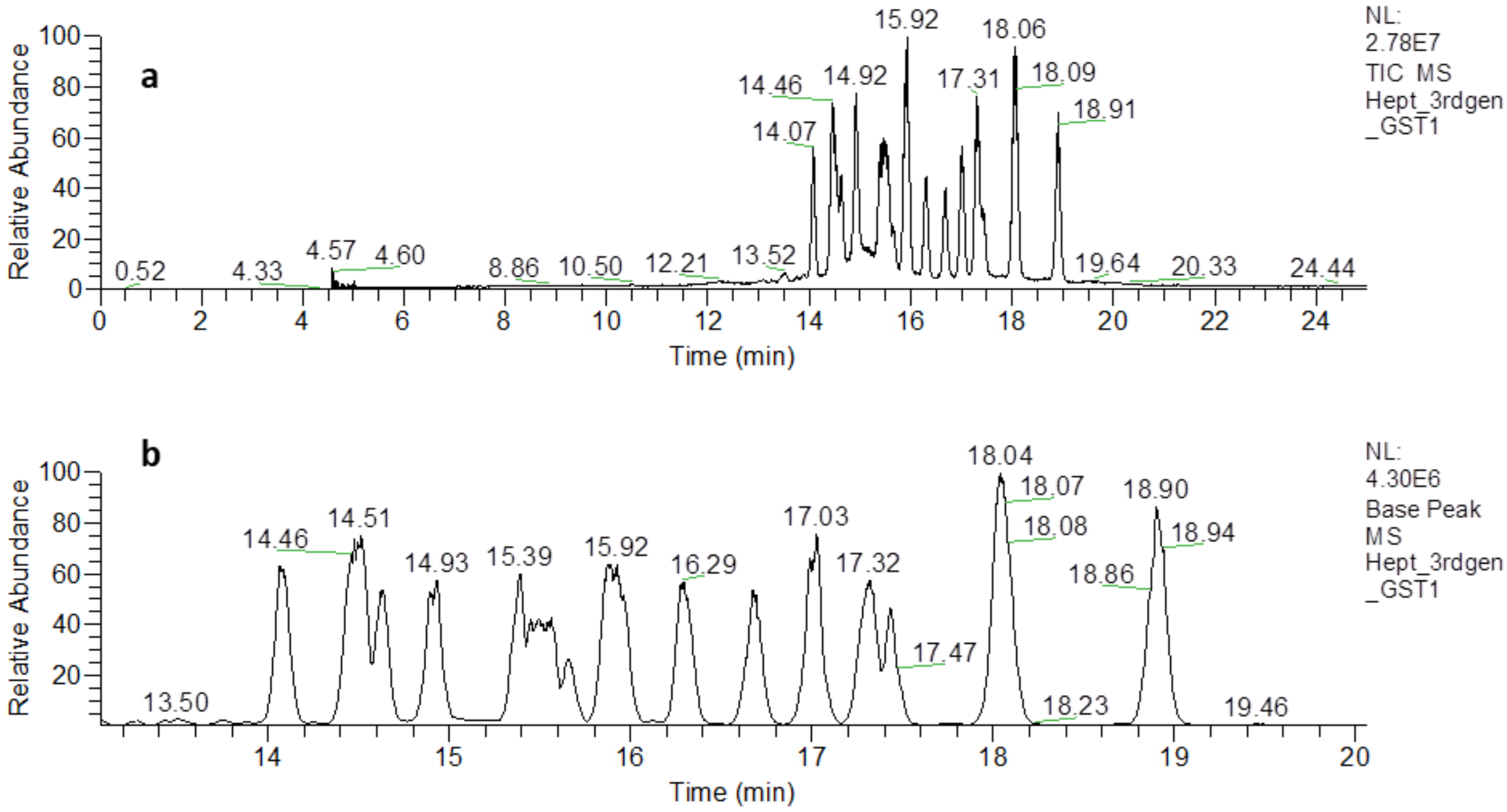

Fig. S23 LCMS chromatograms of **L6.** a) Total ion chromatogram, b) Base-peak chromatogram of compounds in the relevant retention time window. Estimated purity: 91% (Purity estimation was based on integration of total ion chromatogram of the compounds in the relevant retention time window, relative to total peak area of the entire chromatogram.)

Table S7. LC-MS characterization of Library 6 (**L6**), with the following amino acid pattern: ααβααβααβαα including 2 variable positions at hot-spot sites. The listed MS^2 ions were used to identify peptides with the same molecular mass.

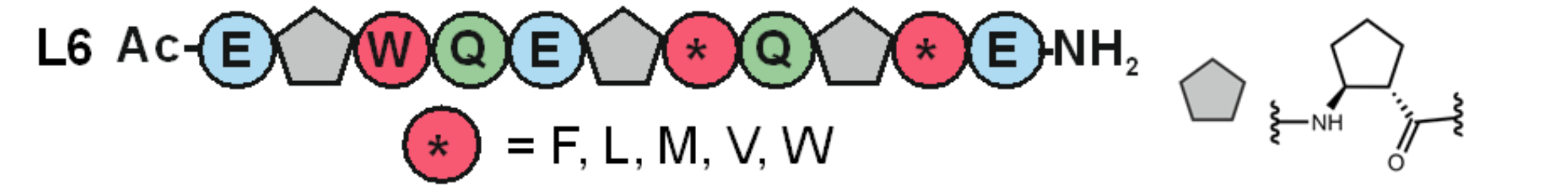

Amino acid at position:		Molecular weight	Exact mass	Detected ions m/z			Retention time (min)[a]
7	10			$[M+H]^+$	$[M+2H]^{2+}$	b5	
V	V	1420.38	1419.63	1420.27	711.50		14.07
L	V	1434.41	1433.64	1434.31	718.08	950.49	15.92
V	L	1434.41	1433.64	1434.35	718.05	936.36	17.02
L	L	1448.44	1447.66	1448.42	725.69		18.90
M	V	1452.45	1451.60	1452.32	727.04	968.44	14.45
V	M	1452.45	1451.60	1452.32	727.04	936.43	14.45
L	M	1466.47	1465.62	1466.34	734.05	950.44	15.88
M	L	1466.47	1465.62	1466.4	734.59	968.34	17.31
F	V	1468.42	1467.63	1468.33	735.53	984.54	14.90
V	F	1468.42	1467.63	1468.41	735.52	936.38	16.30
F	L	1482.45	1481.64	1482.39	742.15	984.4	18.03
L	F	1482.45	1481.64	1482.39	742.15	950.37	18.03
M	M	1484.51	1483.57	1484.27	743.12		14.60
F	M	1500.49	1499.60	1500.36	751.10	984.4	15.38
M	F	1500.49	1499.60	1500.39	751.10	968.41	16.69
V	W	1507.46	1506.64	1507.31	754.59	936.4	14.44
W	V	1507.46	1506.64	1507.45	754.58	1023.53	15.54
F	F	1516.47	1515.63	1516.37	758.78		17.31
L	W	1521.49	1520.65	1522.39	761.57	950.43	15.95
W	L	1521.49	1520.65	1521.37	761.57	1023.42	18.09
M	W	1539.53	1538.61	1539.25	770.60	968.41	14.92
W	M	1539.53	1538.61	1539.34	770.59	1023.39	15.44
F	W	1555.50	1554.64	1555.37	779.05	984.41	15.50
W	F	1555.50	1554.64	1555.32	778.58	1023.42	17.42
W	W	1594.54	1593.65	1594.28	797.78		15.62

[a] Retention time based on measurement on Aeris WP C4 250x4.6 mm column, using a gradient of 5-35-45 % B 25 min, 0.7 ml/min.

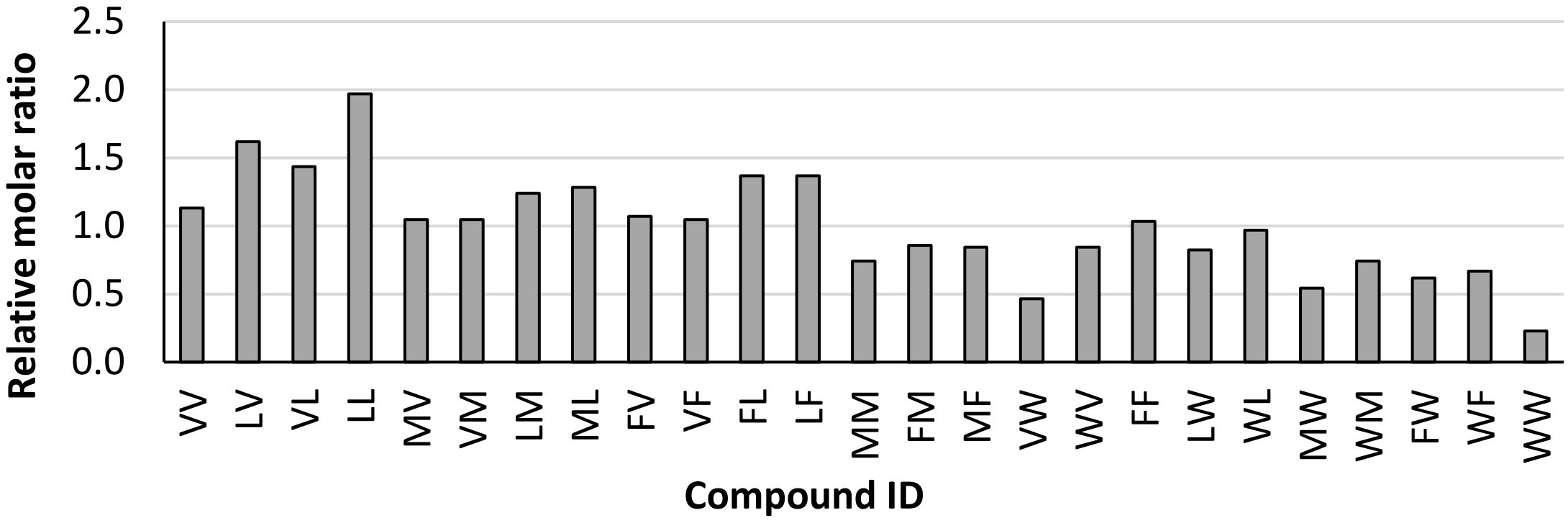

Fig. S24 Estimated equimolarity of **L6** based on LC-MS peak area integration (AUC) of selective ion chromatograms using $[M+H]^+$ charge states with 2 Da window. Compound IDs are generated using the single letter code of the amino acids at positions 7 and 10. Relative molar ratio is estimated using the following formula: $AUC_{compound}$ / (AUC_{total}/25). Due to insufficient separation MV, VM and FL, LF were integrated together.

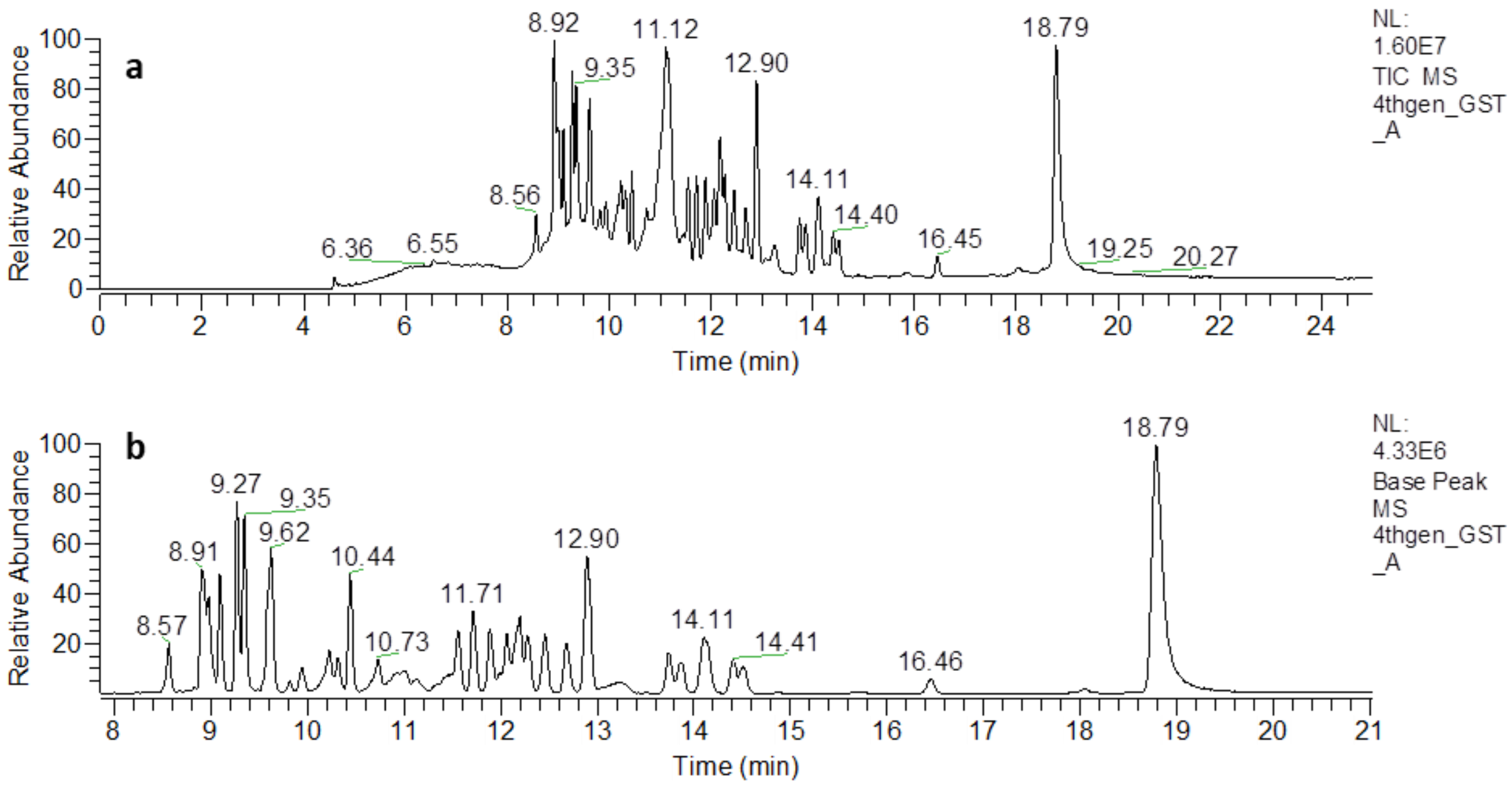

Fig. S25 LCMS chromatograms of **L7.** a) Total ion chromatogram, b) Base-peak chromatogram of compounds in the relevant retention time window. Estimated purity: 75% (Purity estimation was based on integration of total ion chromatogram of the compounds in the relevant retention time window, relative to total peak area of the entire chromatogram.)

Table S8. LC-MS characterization of Library 7 (**L7**), with the following amino acid pattern: ααβααβααβαα including 2 variable positions at hot-spot sites. The listed MS^2 ions were used to identify peptides with the same molecular mass.

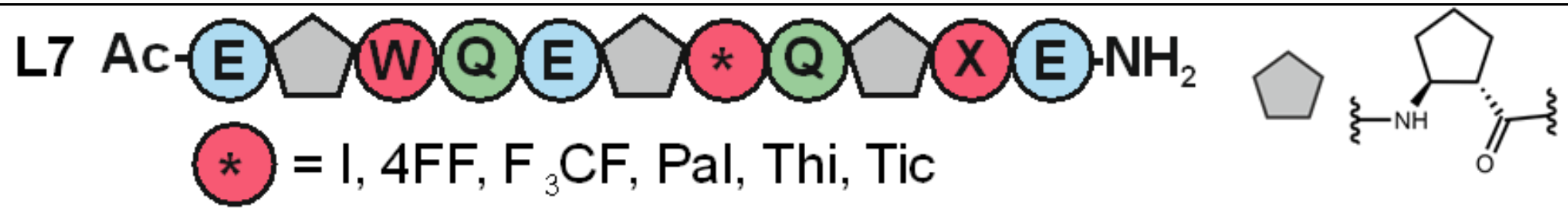

Amino acid at position:		Molecular weight	Exact mass	Detected ions m/z			Retention time (min)[a]
7	10			$[M+H]^+$	$[M+2H]^{2+}$	b7	
I	I	1448.44	1447.66	1448.33	724.74		12.15
I	Pal	1483.46	1482.75	1483.48	742.54	950.27	8.90
Pal	I	1483.46	1482.75	1483.45	742.46	n.d.	9.25
I	Thi	1488.50	1487.79	1488.31	744.90	950.25	11.86
Thi	I	1488.50	1487.79	1488.21	744.69	990.18	11.70
I	Tic	1494.48	1493.77	1494.38	748.57	950.29	10.94
Tic	I	1494.48	1493.77	1494.24	748.51	996.25	10.21
I	4FF	1500.46	1499.75	1500.32	751.11	950.29	12.39
4FF	I	1500.46	1499.75	1500.32	751.09	1002.28	12.23
Pal	Pal	1518.48	1517.83	1518.44	760.28		7.92
Pal	Thi	1523.52	1522.87	1523.43	762.55	985.33	9.35
Thi	Pal	1523.52	1522.87	1523.39	762.61	990.18	8.93
Thi	Thi	1528.56	1527.91	1528.2	765.02		11.53
Pal	Tic	1529.50	1528.85	1529.46	765.49	985.43	8.97
Tic	Pal	1529.50	1528.85	1529.42	765.58	996.22	8.56
Thi	Tic	1534.54	1533.89	1534.18	767.97	990.19	10.98
Tic	Thi	1534.54	1533.89	1534.24	768.09	996.23	10.32
4FF	Pal	1535.48	1534.83	1535.52	769.18	1002.19	9.10
Pal	4FF	1535.48	1534.83	1535.35	769.09	985.29	9.61
4FF	Thi	1540.52	1539.87	1540.21	770.91	1003.23	12.03
Thi	4FF	1540.52	1539.87	1540.16	771.08	990.08	12.09
Tic	Tic	1540.52	1539.87	n.d.	n.d.		n.d.
4FF	Tic	1546.50	1545.85	1546.26	773.94	1002.23	11.44
Tic	4FF	1546.50	1545.85	1546.34	774.04	996.23	10.71
I	F_3CF	1550.47	1549.76	1550.24	776.01	950.23	14.06
F3CF	I	1550.47	1549.76	1550.24	776.01	n.d.	14.06
4FF	4FF	1552.48	1551.83	1552.25	776.91		12.64
F3CF	Pal	1585.49	1584.84	1585.42	793.54	1052.12	9.58
Pal	F3CF	1585.49	1584.84	1585.42	793.61	985.37	10.43
F3CF	Thi	1590.53	1589.88	1590.16	796.62	1052.19	13.69
Thi	F_3CF	1590.53	1589.88	1590.3	796.02	990.14	13.81
F3CF	Tic	1596.51	1595.86	1596.34	799.56	1052.15	13.19
Tic	F_3CF	1596.51	1595.86	1596.18	799.00	996.27	11.96
4FF	F_3CF	1602.49	1601.84	1602.35	802.09	1002.23	14.45
F_3CF	4FF	1602.49	1601.84	1602.3	802.02	1052.17	14.34
F_3CF	F_3CF	1652.50	1651.85	1652.32	827.05		16.37

[a] Retention time based on measurement on Aeris WP C4 250x4.6 mm column, using a gradient of 5-40-55 % B 25 min, 0.7 ml/min. I: isoleucine, Pal: 3-(3-pyridyl)-L-alanine, Thi: 3-thienyl-L-alanine, Tic: (*S*)-1,2,3,4-tetrahydroisoquinoline-3-carboxylic acid, 4FF: 4-fluorophenylalanine, F_3CF: 4-trifluoromethyl-L-phenylalanine.

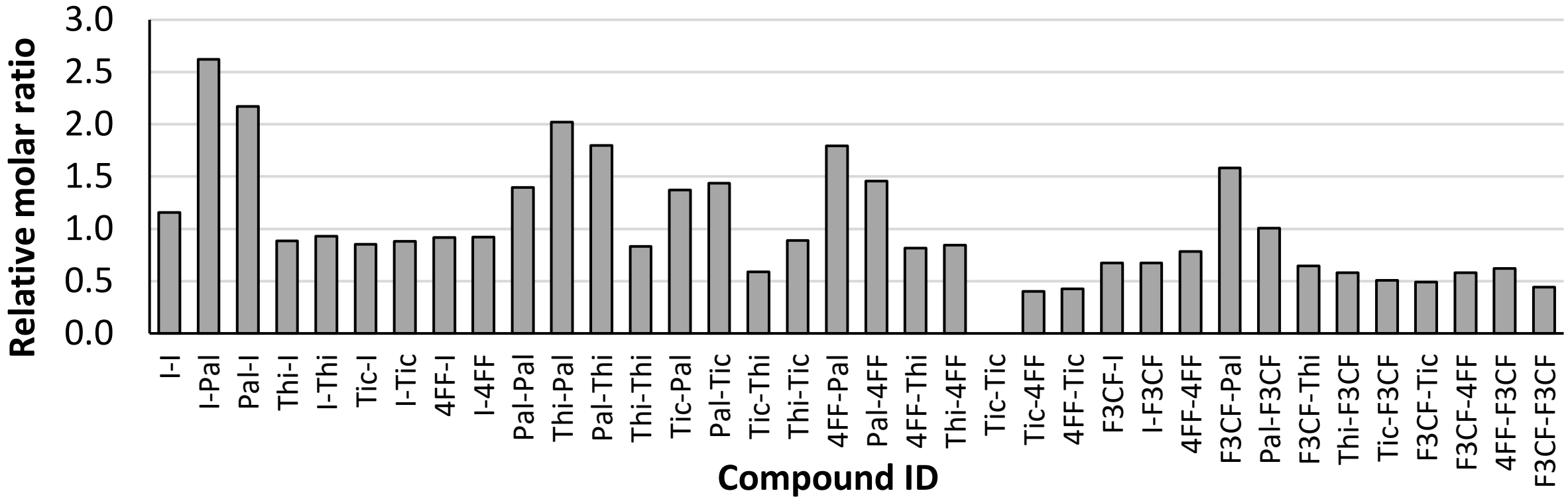

Fig. S26 Estimated equimolarity of **L7** based on LC-MS peak area integration (AUC) of selective ion chromatograms using $[M+H]^+$ charge states with 2 Da window. Compound IDs are generated using the amino acid abbreviations at positions 7 and 10. Relative molar ratio is estimated using the following formula: $AUC_{compound}$ / (AUC_{total}/36). Due to insufficient separation I-F3CF and F3CF-I were integrated together. I: isoleucine, Pal: 3-(3-pyridyl)-L-alanine, Thi: 3-thienyl-L-alanine, Tic: (*S*)-1,2,3,4-tetrahydroisoquinoline-3-carboxylic acid, 4FF: 4-fluorophenylalanine, F3CF: 4-trifluoromethyl-L-phenylalanine.

Table S9. LC-MS characterization of the disulphide dimer library (**DL1**) used in the DCL experiment. GSH = glutathione.

DL1

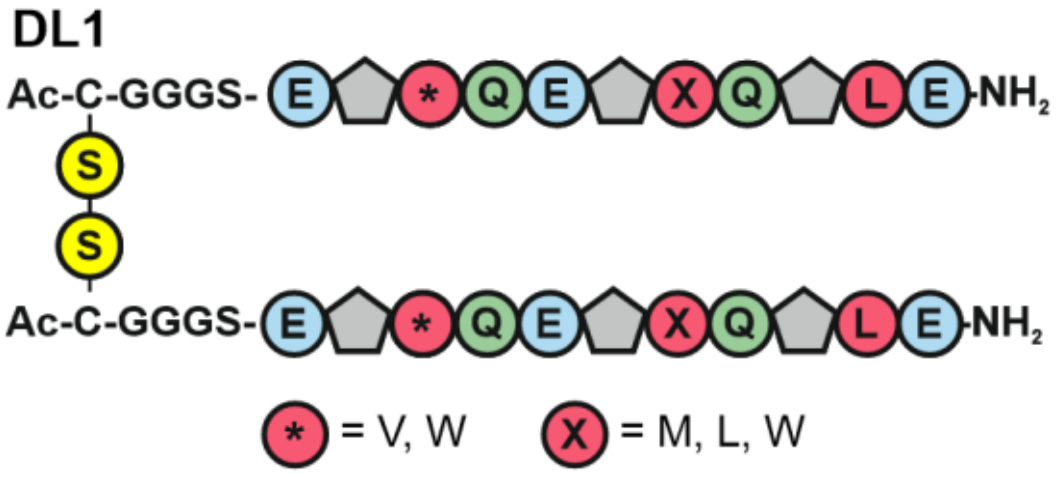

Monomer		Molecular weight	Detected ions m/z		Retention time (min)[a]
1	2		$[M+2H]^{2+}$	$[M+3H]^{3+}$	
VLL	-	1722.96	862.58		13.80
VML	-	1740.99	871.09		12.90
VWL	-	1796.01	899.1		13.15
WLL	-	1810.04	906.1		14.14
WML	-	1828.07	915.07		13.43
WWL	-	1883.09	942.1		13.86
VLL	GSH	2027.96	1014.78		12.01
VML	GSH	2045.99	1023.84		11.35
VWL	GSH	2101.01	1051.26		11.61
WLL	GSH	2115.04	1058.26		12.50
WML	GSH	2133.07	1067.27		11.94
WWL	GSH	2188.09	1094.79		12.31
VLL	VLL	3443.92		1149.18	18.13
VLL	VML	3461.95		1154.98	17.48
VML	VML	3479.98		1160.87	16.84
VWL	VLL	3516.97		1173.12	17.65
VWL	VML	3535.00		1179.55	17.01
VWL	VWL	3590.02		1197.80	17.20
WLL	VLL	3531.00		1177.91	18.36
WLL	VML	3549.03		1184.08	17.80
WLL	VWL	3604.05		1202.00	17.96
WLL	WLL	3618.08		1206.69	18.58
WLL	WML	3636.11		1212.71	18.09
WML	VLL	3549.03		1183.43	17.93
WML	VML	3567.06		1189.82	17.23
WML	VWL	3622.08		1208.21	17.46
WML	WML	3654.14		1218.65	17.59
WWL	VLL	3604.05		1202.17	18.21
WWL	VML	3622.08		1208.01	17.56
WWL	VWL	3677.10		1226.33	17.75
WWL	WLL	3691.13		1230.94	18.43
WWL	WML	3709.16		1237.63	17.92
WWL	WWL	3764.18		1255.80	18.21

Retention time based on measurement on Aeris WP C4 250x4.6 mm column, using a gradient of 5-35-60 % B 25 min (0 min 5% B, 5 min 35%B, 25 min 60% B, 0.7 ml/min.)

Table S10. LC-MS characterisation of Library **L8-SH**, with the following amino acid pattern: ααβααβααβαα including 3 variable positions at hot-spot sites. The peptides were synthesised with a cysteine residue at the N terminus to facilitate dimerization with Cl-W(F_3CF) to generate L9. PEG2: amino-3,6-dioxaoctylsuccinamic acid.

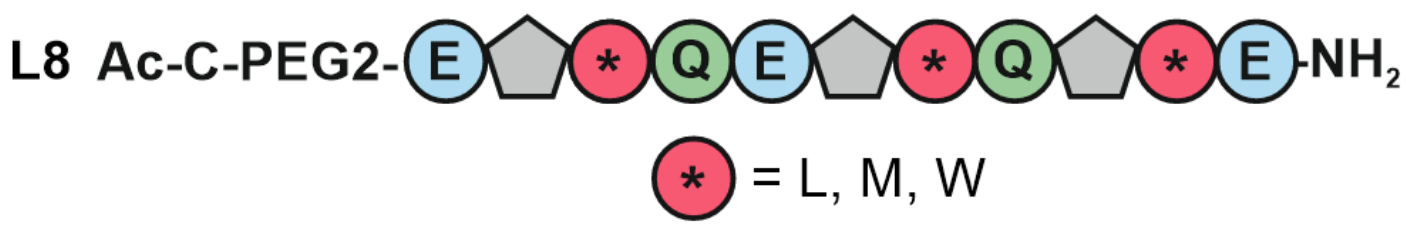

Amino acid at position:			Molecular weight	Exact mass	Detected ions m/z				Retention time (min)[a]
3	7	10			$[M+H]^+$	$[M+2H]^{2+}$	b5	b6	
L	L	L	1707.79	1706.79	1709.95	855.33			15.78
L	L	M	1725.75	1724.75	1727.93	864.34	1210.70	n.d.	14.16
L	M	L	1725.75	1724.75	1727.92	864.40	1228.63	n.d.	14.90
M	L	L	1725.75	1724.75	1727.91	864.36	1228.59	1115.75	13.90
L	M	M	1743.71	1742.71	1744.86	873.22	1228.64	n.d.	13.41
M	L	M	1743.71	1742.71	1745.88	873.32	1228.55	1115.67	12.58
M	M	L	1743.71	1742.71	1745.88	873.33	1246.51	n.d.	13.24
M	M	M	1761.66	1760.66	1763.86	882.31			12.02
W	L	L	1780.79	1779.79	1782.96	891.78	1283.66	1170.71	14.84
L	L	W	1780.79	1779.79	1782.92	891.83	1210.67	n.d.	14.01
L	W	L	1780.79	1779.79	1782.96	891.82	1283.69	n.d.	15.26
W	L	M	1798.75	1797.75	1800.89	900.93	1283.59	n.d.	13.80
W	M	L	1798.75	1797.75	1800.88	900.82	1301.56	1170.80	14.18
L	M	W	1798.75	1797.75	1800.94	900.75	1228.56	1097.68	13.40
L	W	M	1798.75	1797.75	1800.89	900.75	1228.56	n.d.	13.50
M	L	W	1798.75	1797.75	1800.95	900.92	1228.54	1115.55	12.64
M	W	L	1798.75	1797.75	1800.89	900.79	1301.64	n.d.	13.74
W	M	M	1816.70	1815.70	1818.92	909.83	1301.68	1170.62	12.86
M	M	W	1816.70	1815.70	1818.93	909.88	1246.53	n.d.	12.19
M	W	M	1816.70	1815.70	1818.93	909.81	1301.68	n.d.	12.50
W	L	W	1853.78	1852.78	1856.12	928.28	1283.70	1170.73	13.55
W	W	L	1853.78	1852.78	1856.14	928.33	1356.64	n.d.	14.55
L	W	W	1853.78	1852.78	1856.14	928.32	1283.67	1097.74	13.80
W	M	W	1871.74	1870.74	1874.18	937.32	1301.59	1170.64	13.05
W	W	M	1871.74	1870.74	1874.17	937.31	1356.69	n.d.	13.32
M	W	W	1871.74	1870.74	1874.15	937.34	1301.54	n.d.	12.63
W	W	W	1926.78	1925.78	1929.44	964.82			13.45

Retention time based on measurement on Aeris WP C4 250x4.6 mm column, using a gradient of 5-35-60 % B 25 min, 0.7 ml/min

Table S11. LC-MS characterisation of the thioether dimer library (**DL2**) containing $W(CF_3F)_2$ as a fixed monomer conjugated to **L8** to form dimers. The dimers were created through a stable thioether linkage between the chloroacetyl modified $W(F_3CF)_2$ and the members of **L8.**

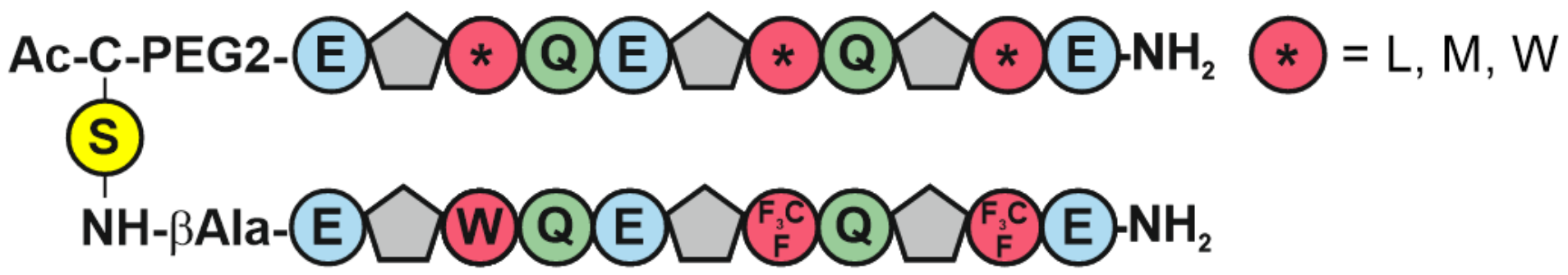

Amino acid at position:			Molecular weight	Exact mass	Detected ions m/z			Retention time (min)[a]
3	7	10			$[M+H]^+$	$[M+2H]^{2+}$	$[M+3H]^{3+}$	
W	F_3CF	F_3CF	1757.76	1756.76	1757.76	879.82		19.03
L	L	L	3430.05	3429.05		1715.53	1144.02	23.12
L	L	M	3448.01	3447.01		1724.51	1150.00	21.9
L	M	L	3448.01	3447.01		1724.51	1150.00	22.51
M	L	L	3448.01	3447.01		1724.51	1150.00	21.71
L	M	M	3465.97	3464.97		1733.48	1155.99	21.39
M	L	M	3465.97	3464.97		1733.48	1155.99	20.75
M	M	L	3465.97	3464.97		1733.48	1155.99	21.25
M	M	M	3483.92	3482.92		1742.46	1161.97	20.32
W	L	L	3503.05	3502.05		1752.02	1168.35	22.36
L	L	W	3503.05	3502.05		1752.02	1168.35	21.67
L	W	L	3503.05	3502.05		1752.02	1168.35	22.63
W	L	M	3521.01	3520.01		1761.00	1174.34	21.62
W	M	L	3521.01	3520.01		1761.00	1174.34	21.85
L	M	W	3521.01	3520.01		1761.00	1174.34	21.36
L	W	M	3521.01	3520.01		1761.00	1174.34	21.36
M	L	W	3521.01	3520.01		1761.00	1174.34	20.71
M	W	L	3521.01	3520.01		1761.00	1174.34	21.56
W	M	M	3538.96	3537.96		1769.98	1180.32	20.91
M	M	W	3538.96	3537.96		1769.98	1180.32	20.41
M	W	M	3538.96	3537.96		1769.98	1180.32	20.67
W	L	W	3576.04	3575.04		1788.52	1192.68	21.32
W	W	L	3576.04	3575.04		1788.52	1192.68	22.06
L	W	W	3576.04	3575.04		1788.52	1192.68	21.49
W	M	W	3594.00	3593.00		1797.50	1198.67	21.00
W	W	M	3594.00	3593.00		1797.50	1198.67	21.18
M	W	W	3594.00	3593.00		1797.50	1198.67	20.73
W	W	W	3649.04	3648.04		1825.02	1217.01	21.22

[a]Retention time based on measurement on Aeris WP C4 250x4.6 mm column, using a gradient of 5-35-60% B, 25min, 0.7ml/min. Retention time for compounds with the same molecular weight was determined based on relative retention times detected for L8 monomers (Table S10.)

4 Compound characterization

MLL Ac-GNILPSDIMDFVLKNTP-NH_2

MW: 1915.219; Exact mass: 1913.981

$[M+H]^+$: 1917.19. $[M+2H]^{2+}$: 959.09.

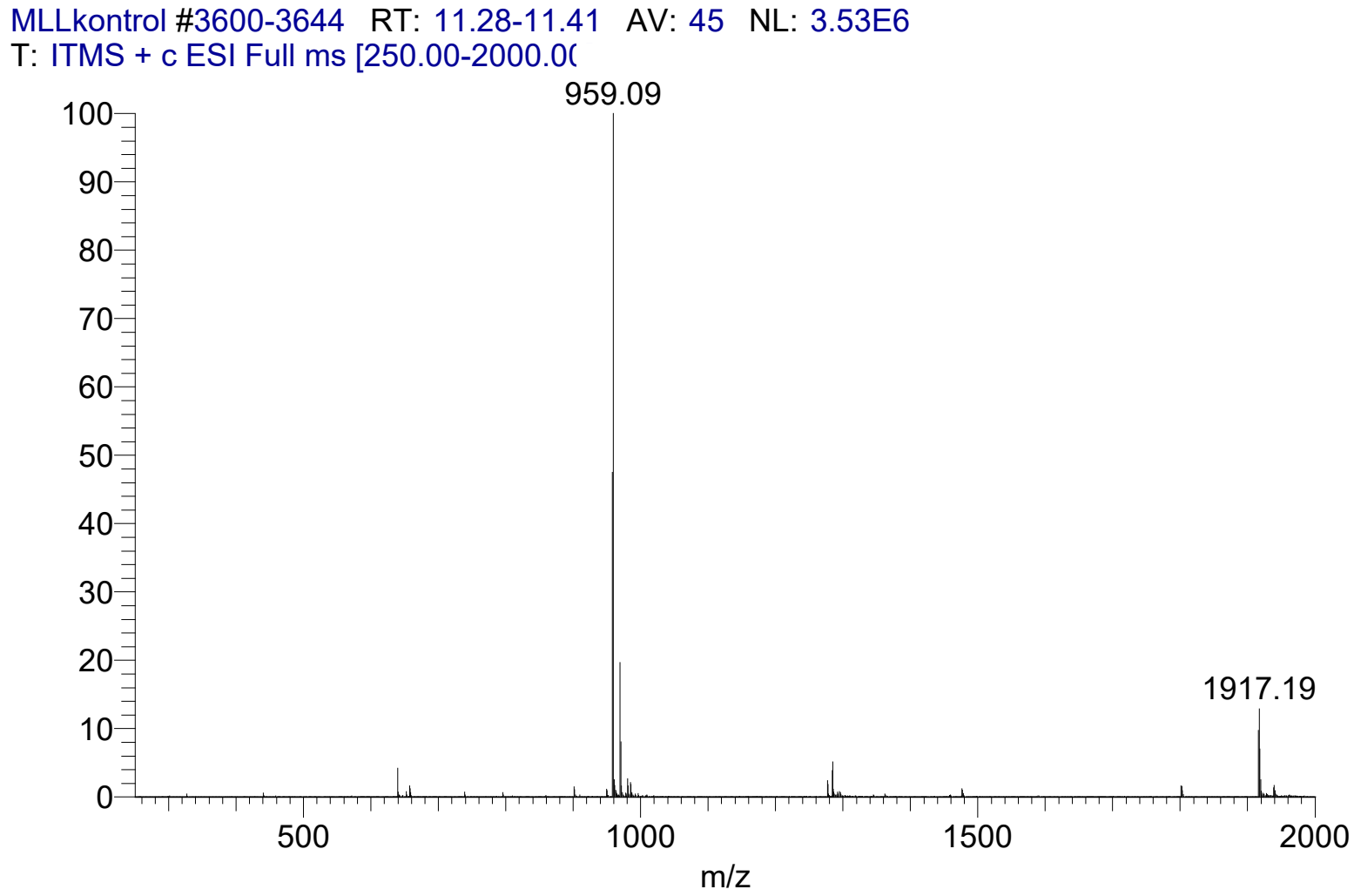

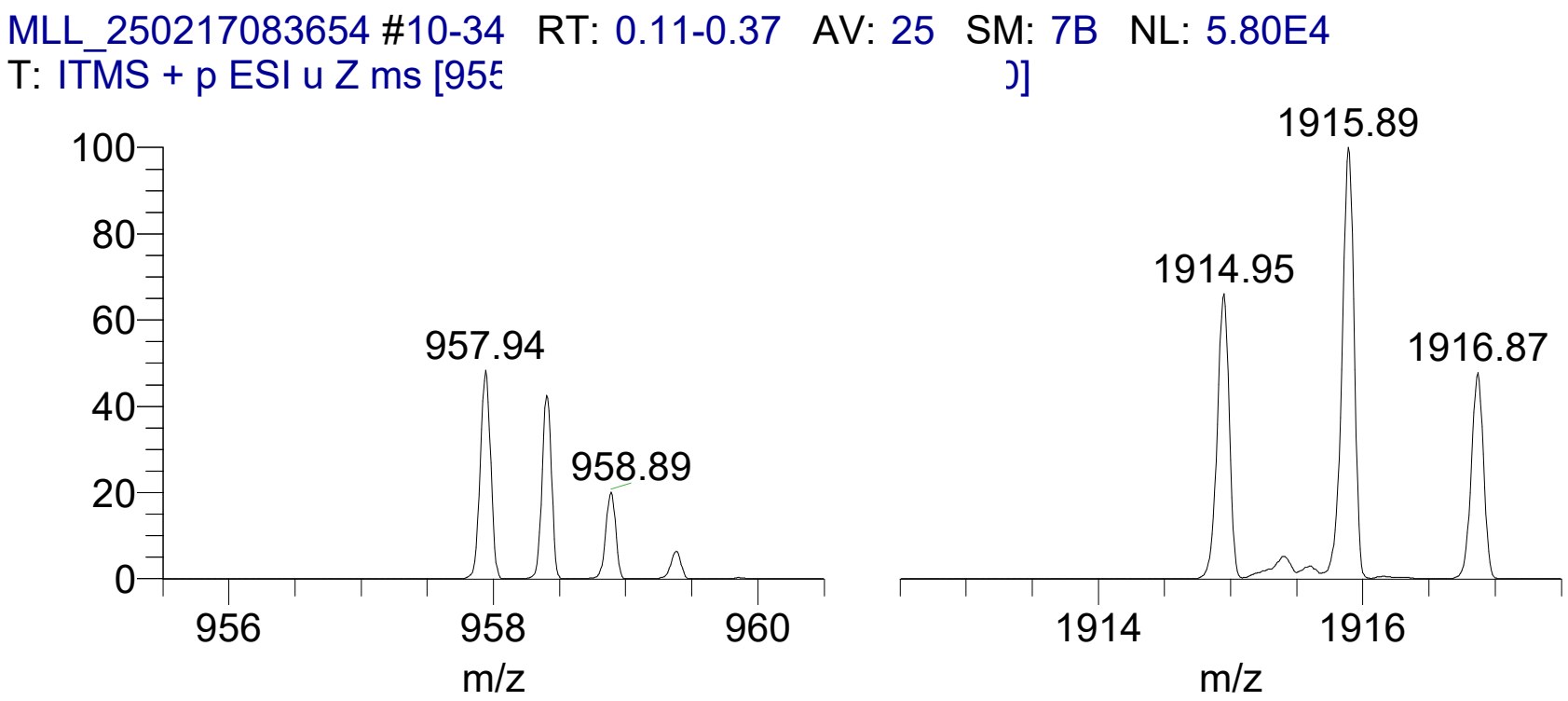

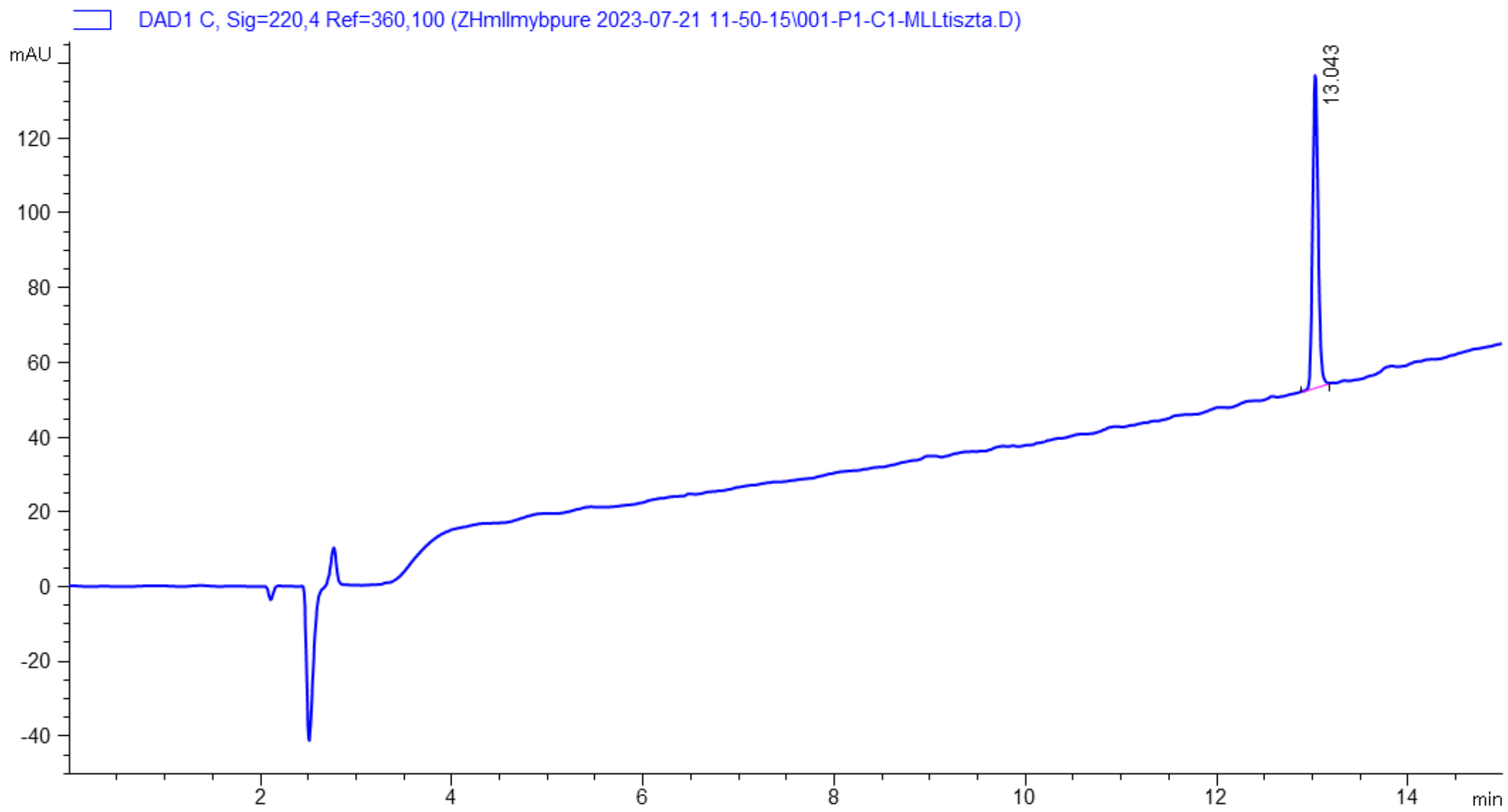

MYB Ac-KEKRIKELELLLMSTENELKGQQAL-NH_2

2983.500 (av.)

2981.648 (mono.)

$[M+2H]^{2+}$: 1493.41. $[M+3H]^{3+}$: 995.86. $[M+4H]^{4+}$: 747.26. $[M+5H]^{5+}$: 598.00

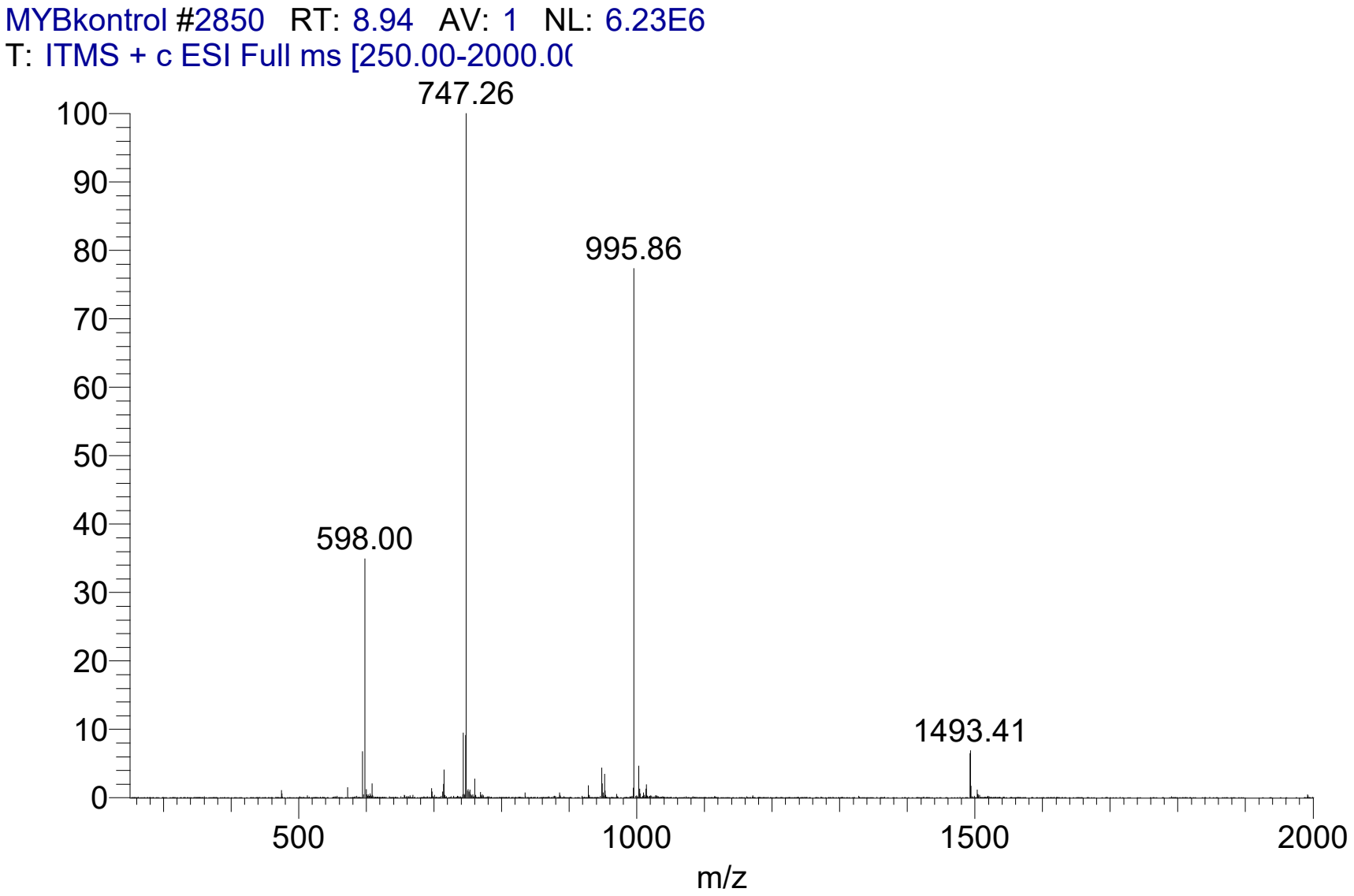

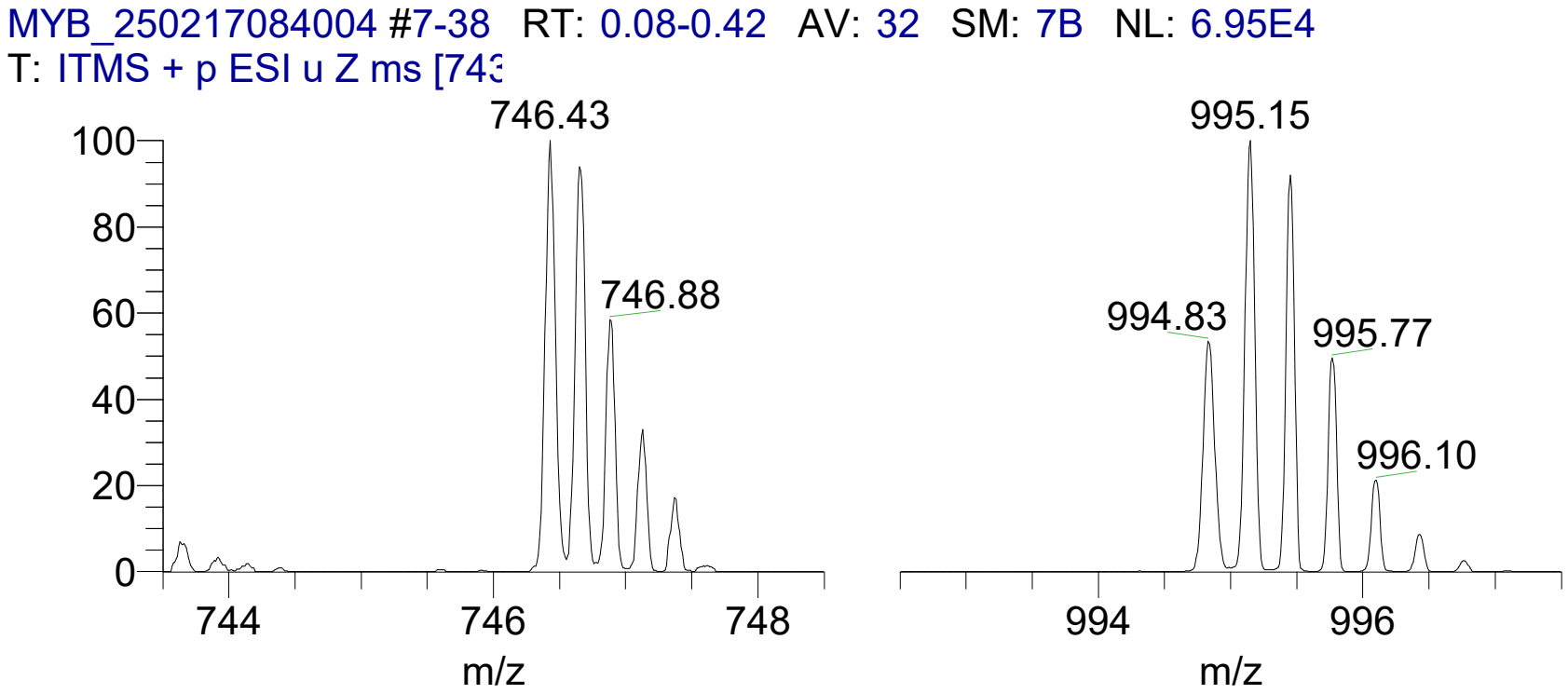

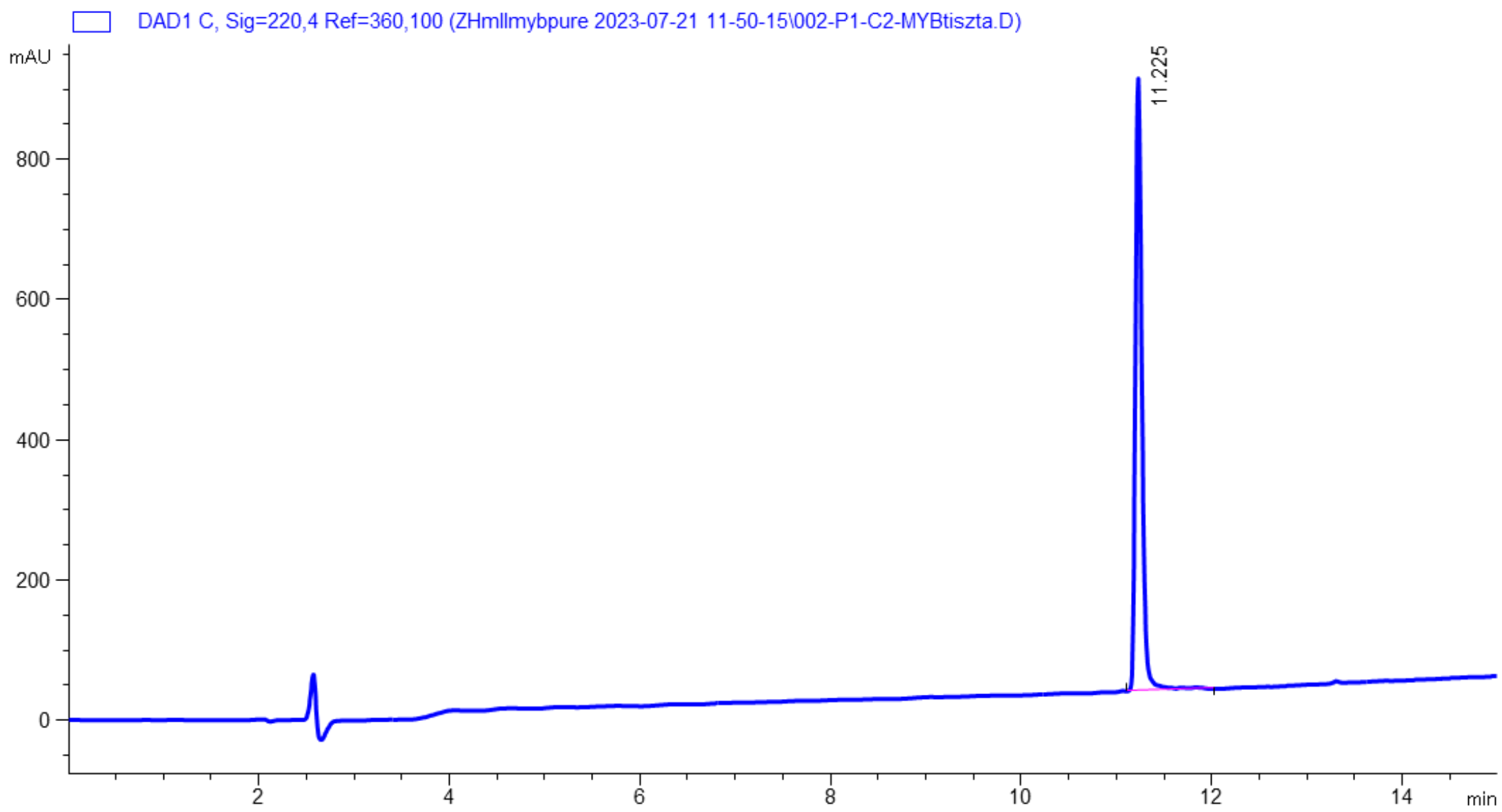

Flu-MLL

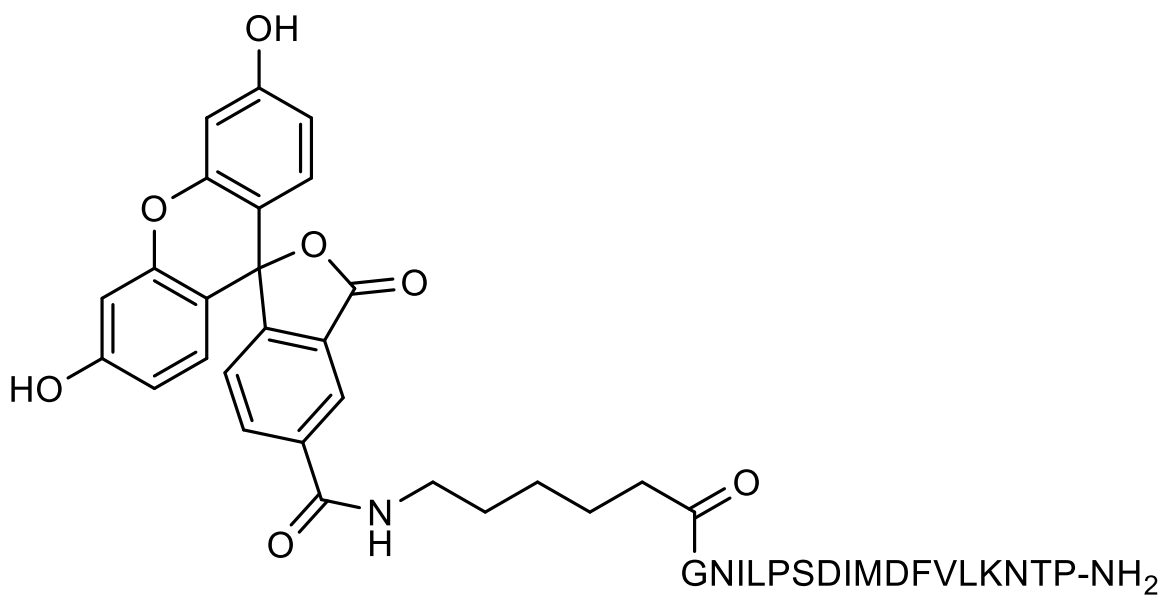

Exact Mass: 2343.110; Molecular Weight: 2344.559

$[M+2H]^{2+}$:1172.91. $[M+3H]^{3+}$:782.56

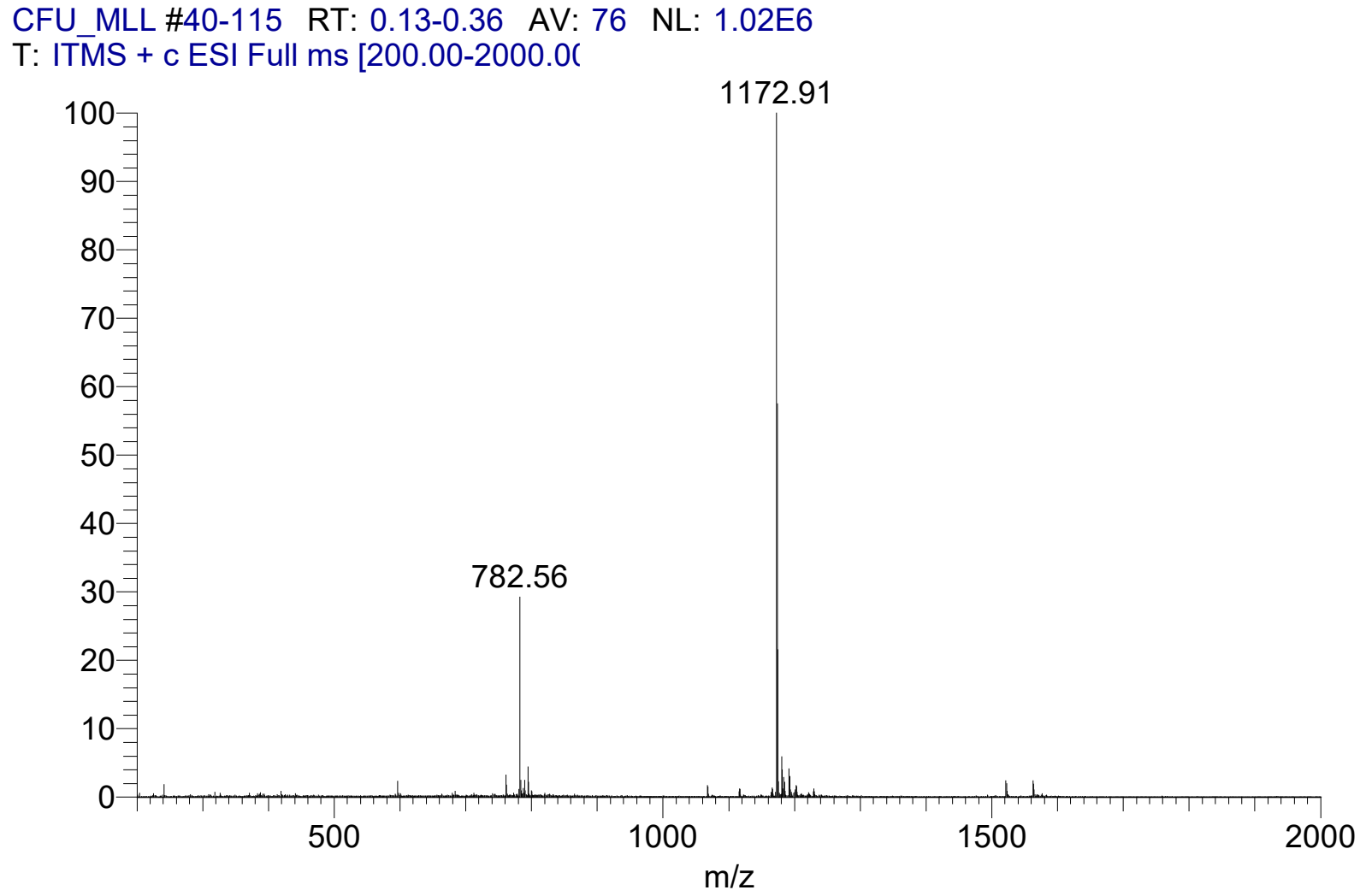

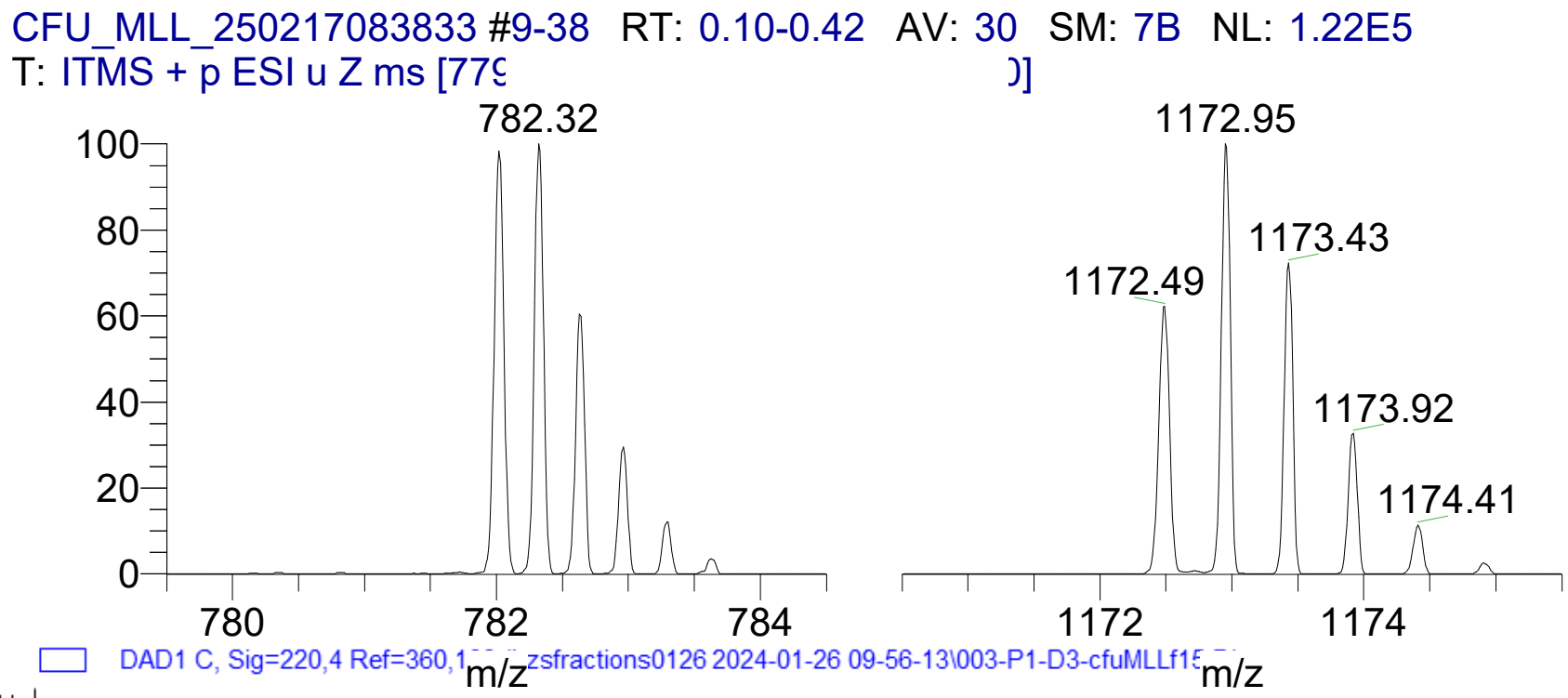

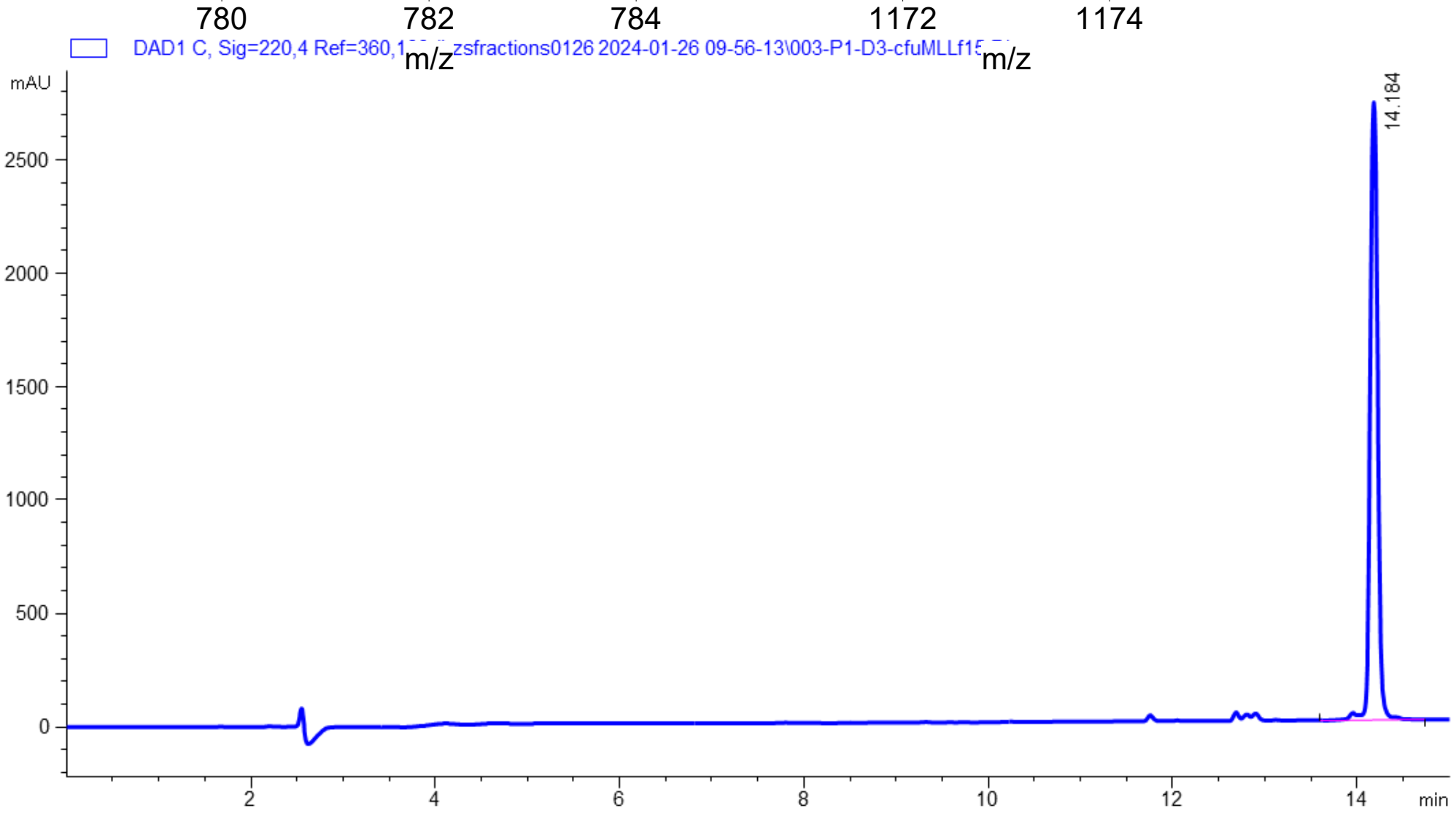

Flu-MYB

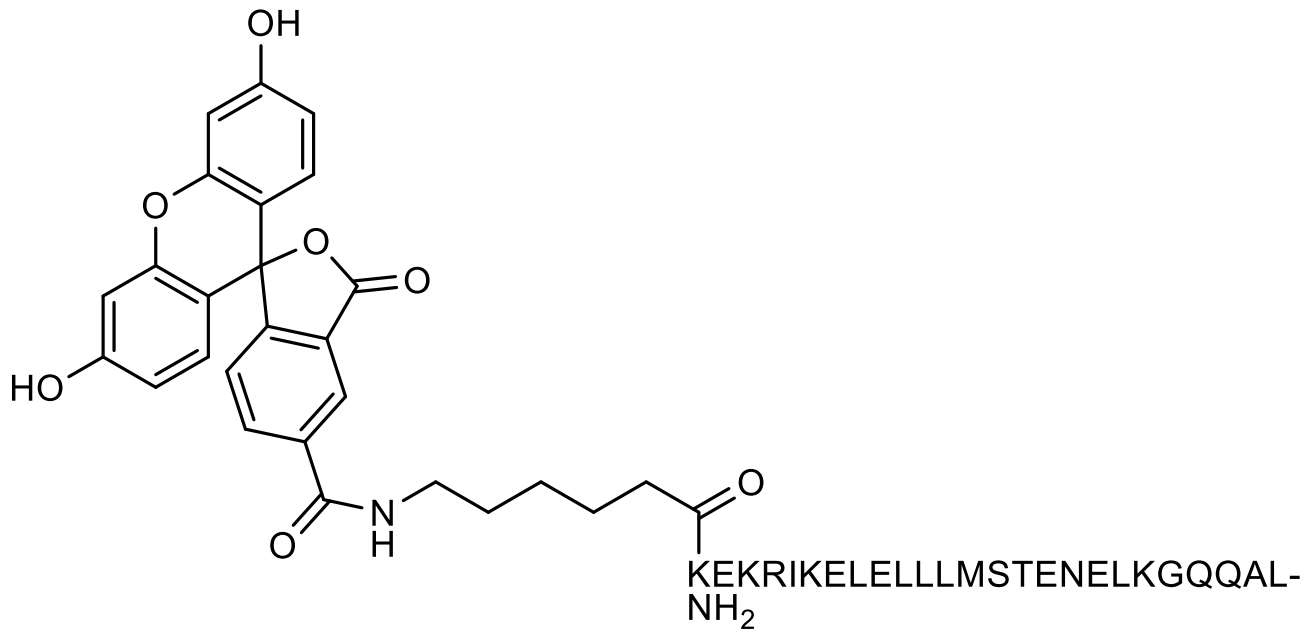

3412.936 (av.)

3412.629 (mono.)

$[M+3H]^{3+}$: 1138.71. $[M+4H]^{4+}$: 854.27. $[M+5H]^{5+}$: 683.66.

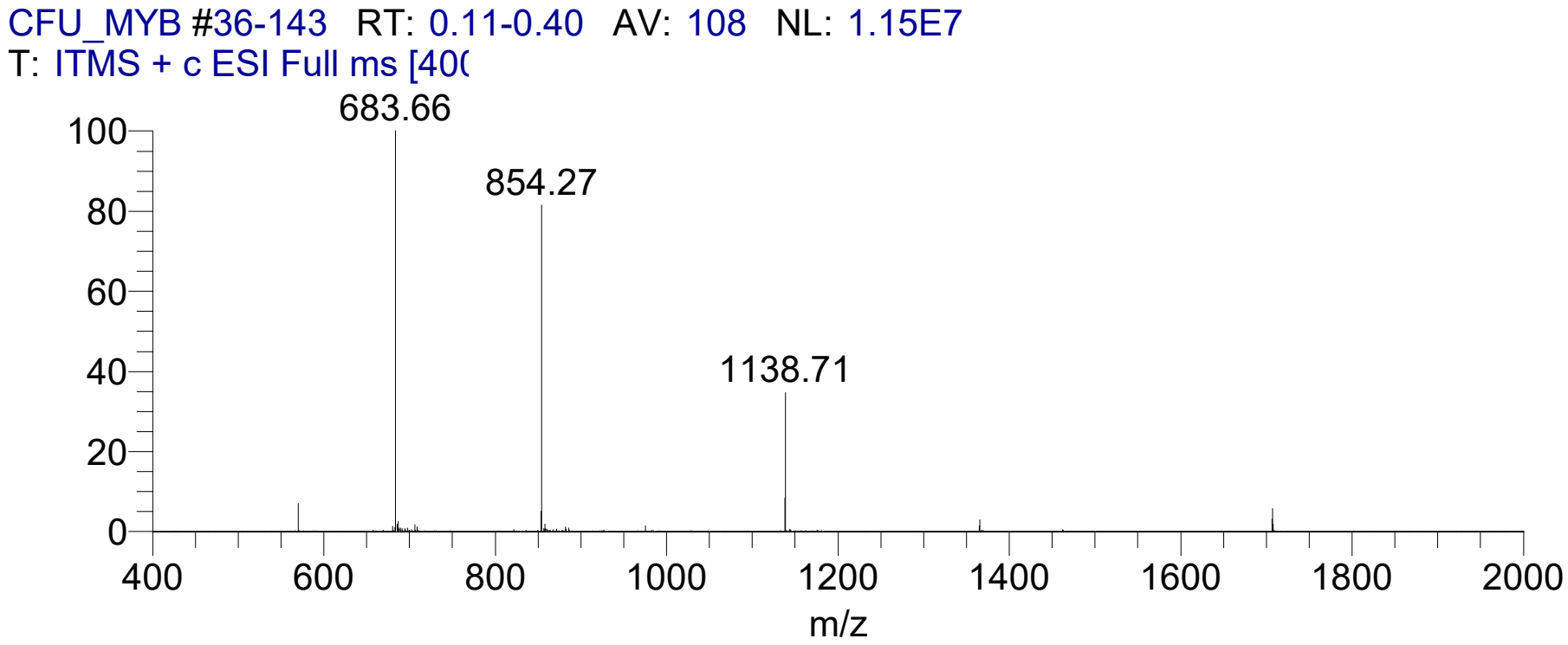

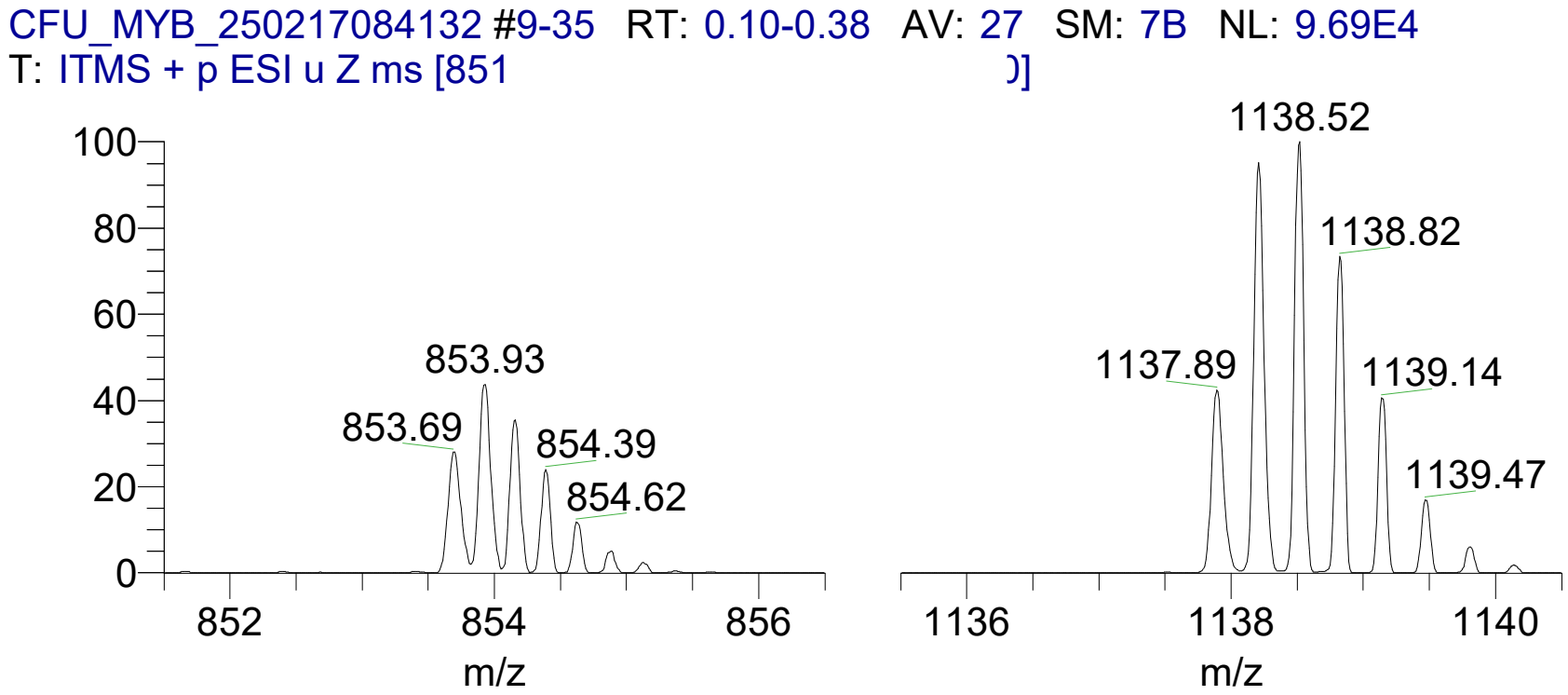

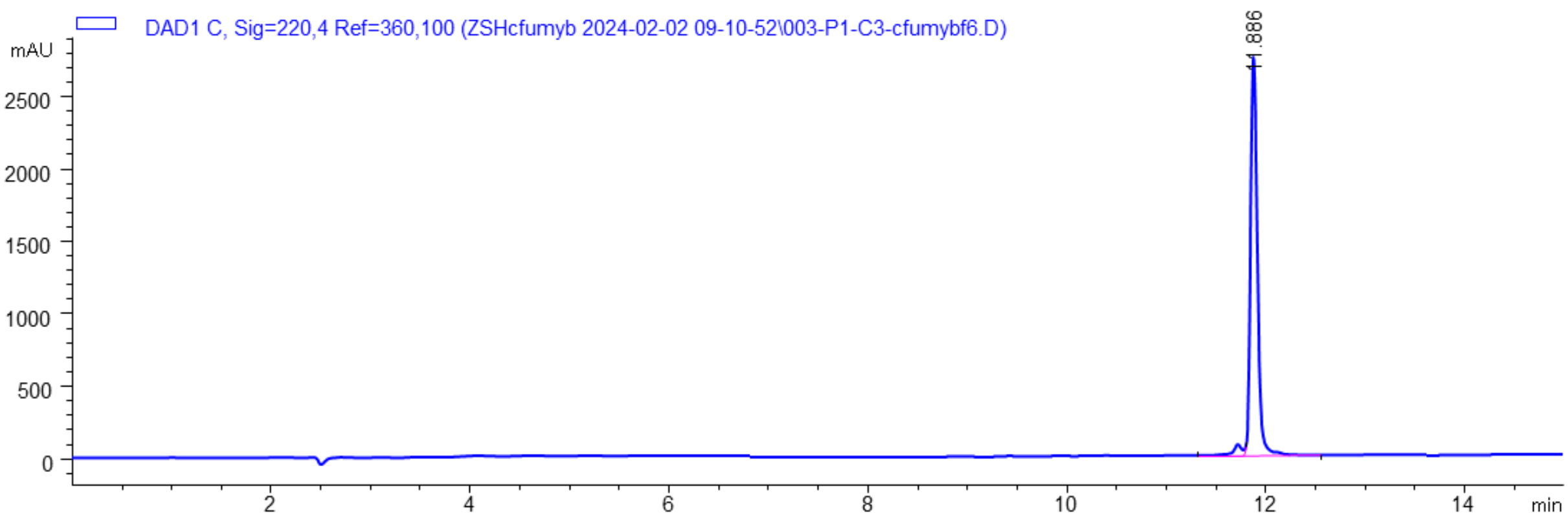

L1-WLL

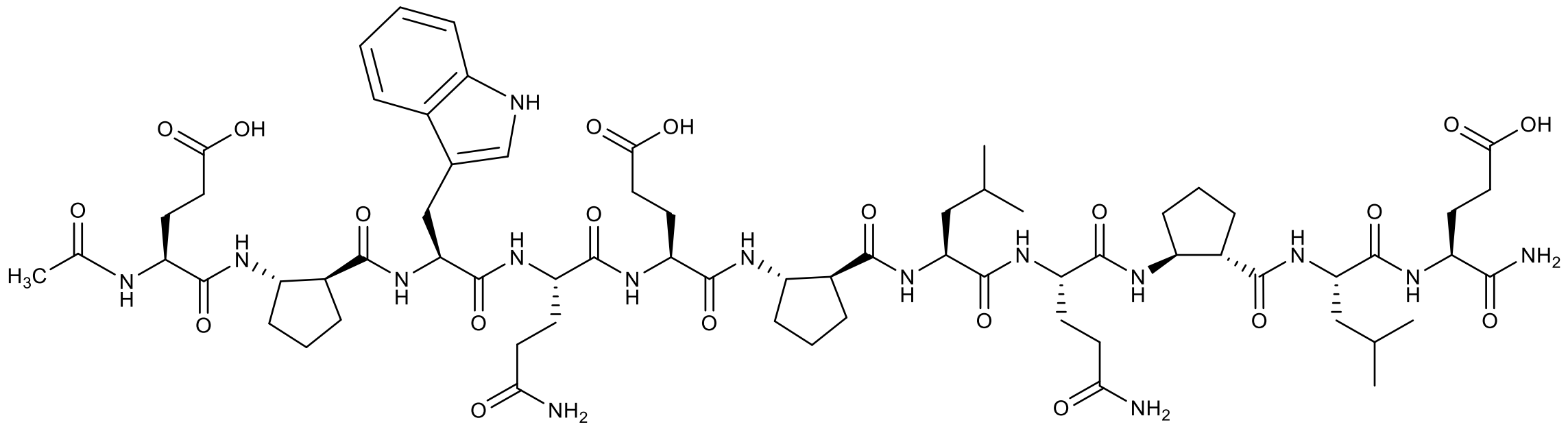

Exact Mass: 1447.735; Molecular Weight: 1448.641

$[M+H]^+$: 1448.39. $[M+2H]^{2+}$: 725.01

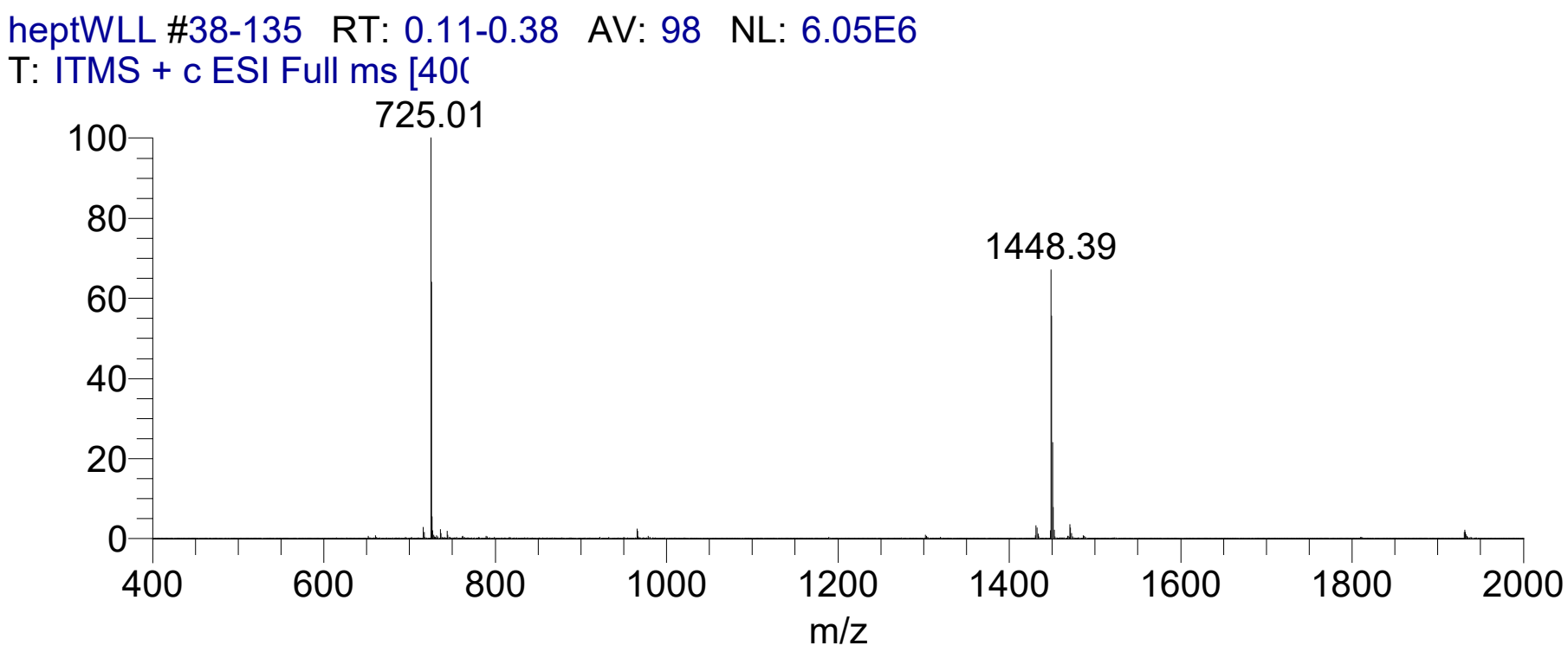

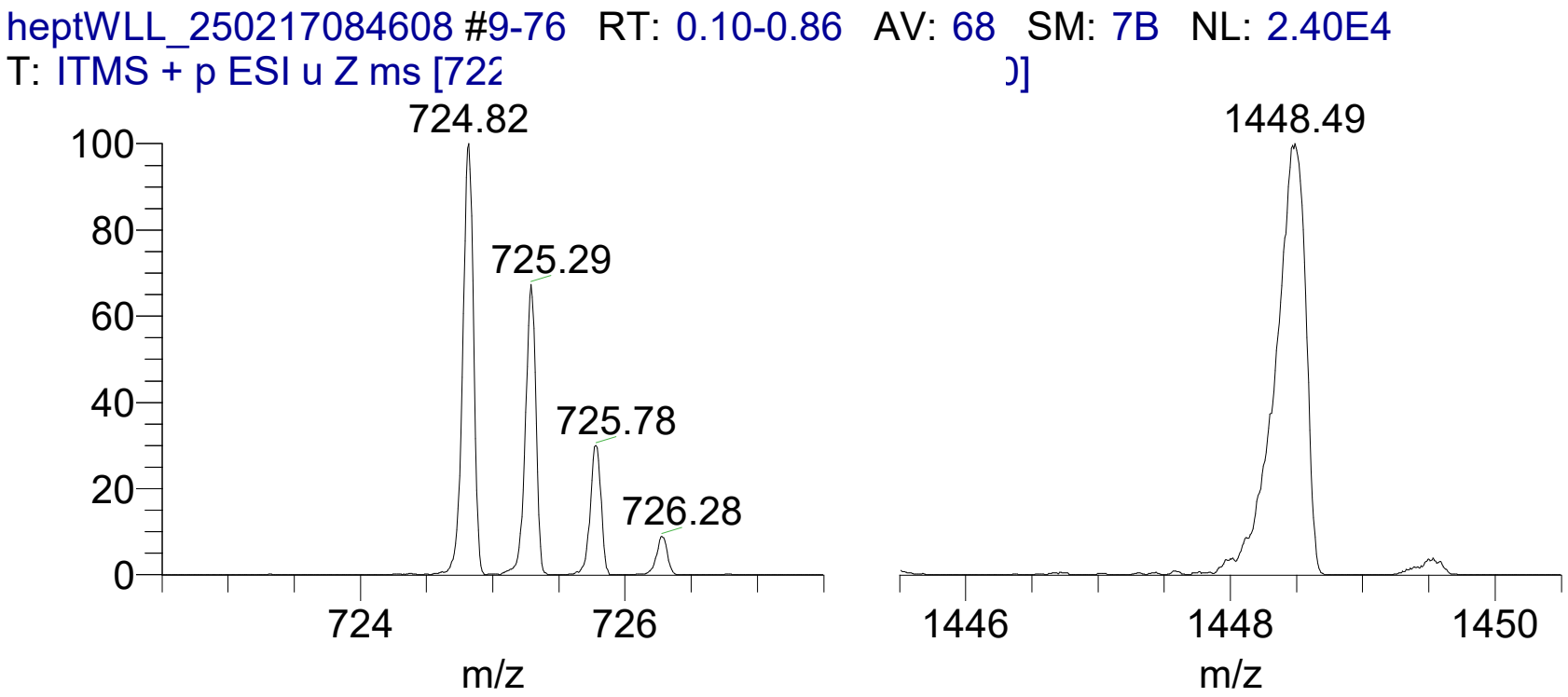

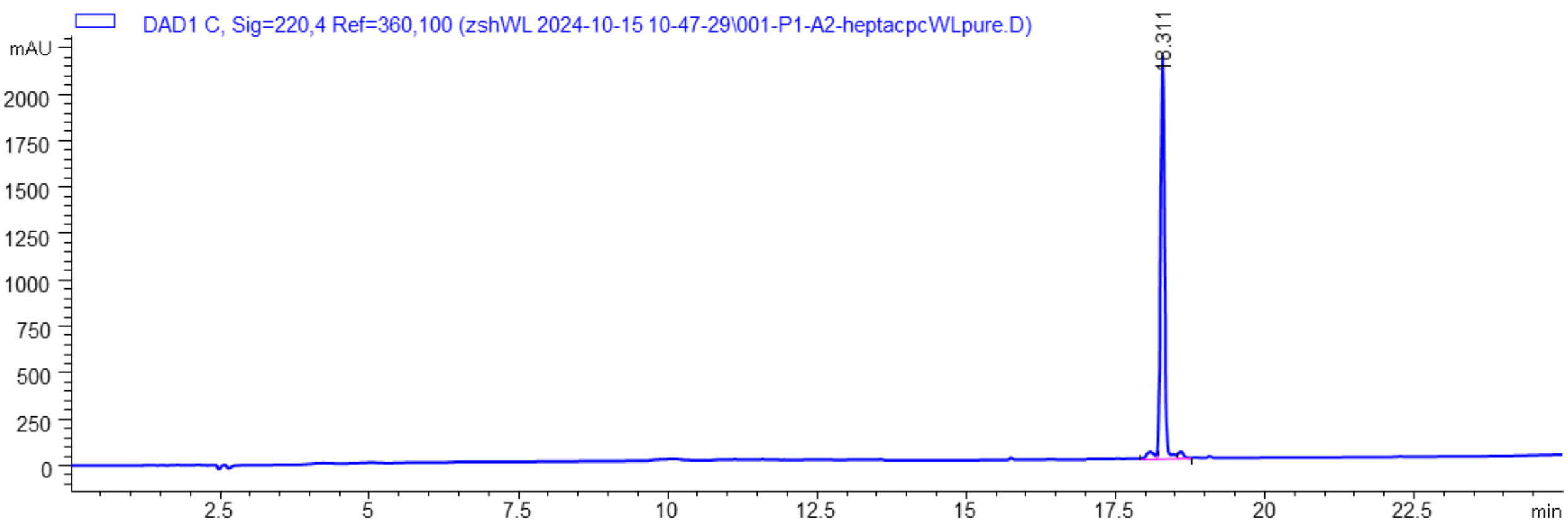

L7 – W(CF$_3$F)$_2$

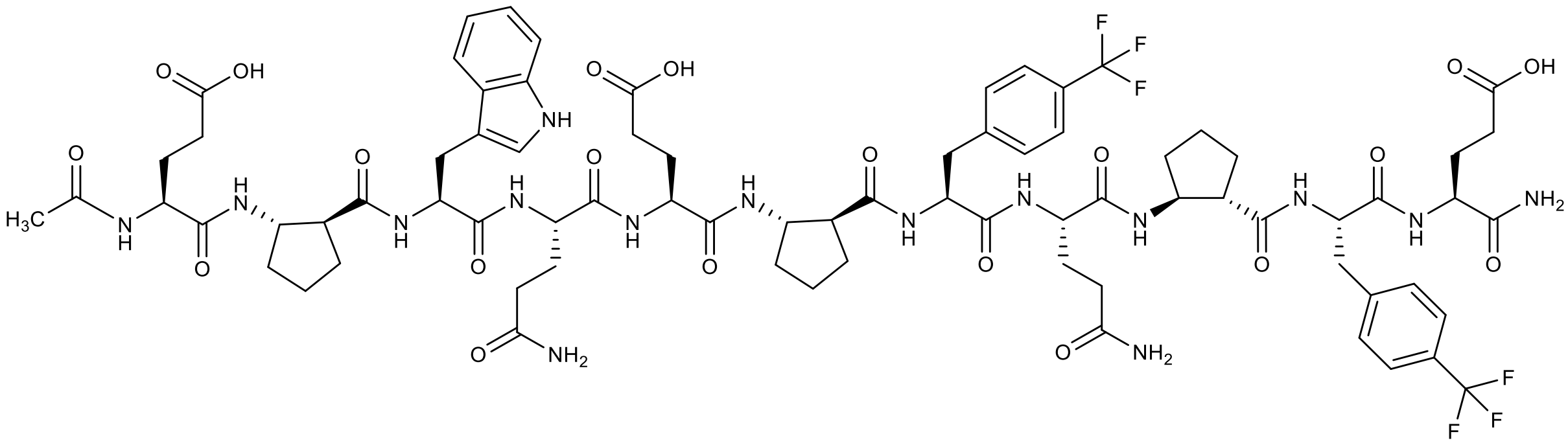

Exact Mass: 1651.678; Molecular Weight: 1652.671

$[M+H]^+$: 1652.43. $[M+2H]^{2+}$: 827.35

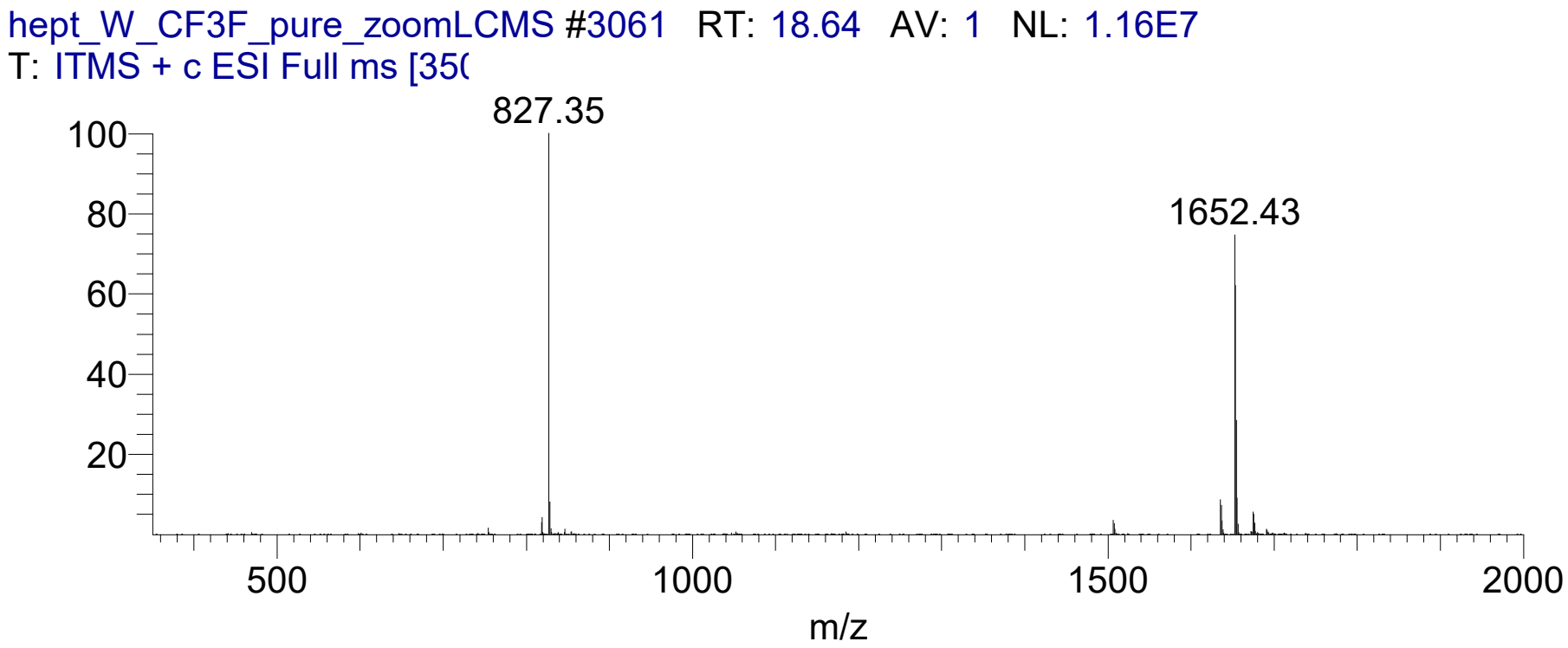

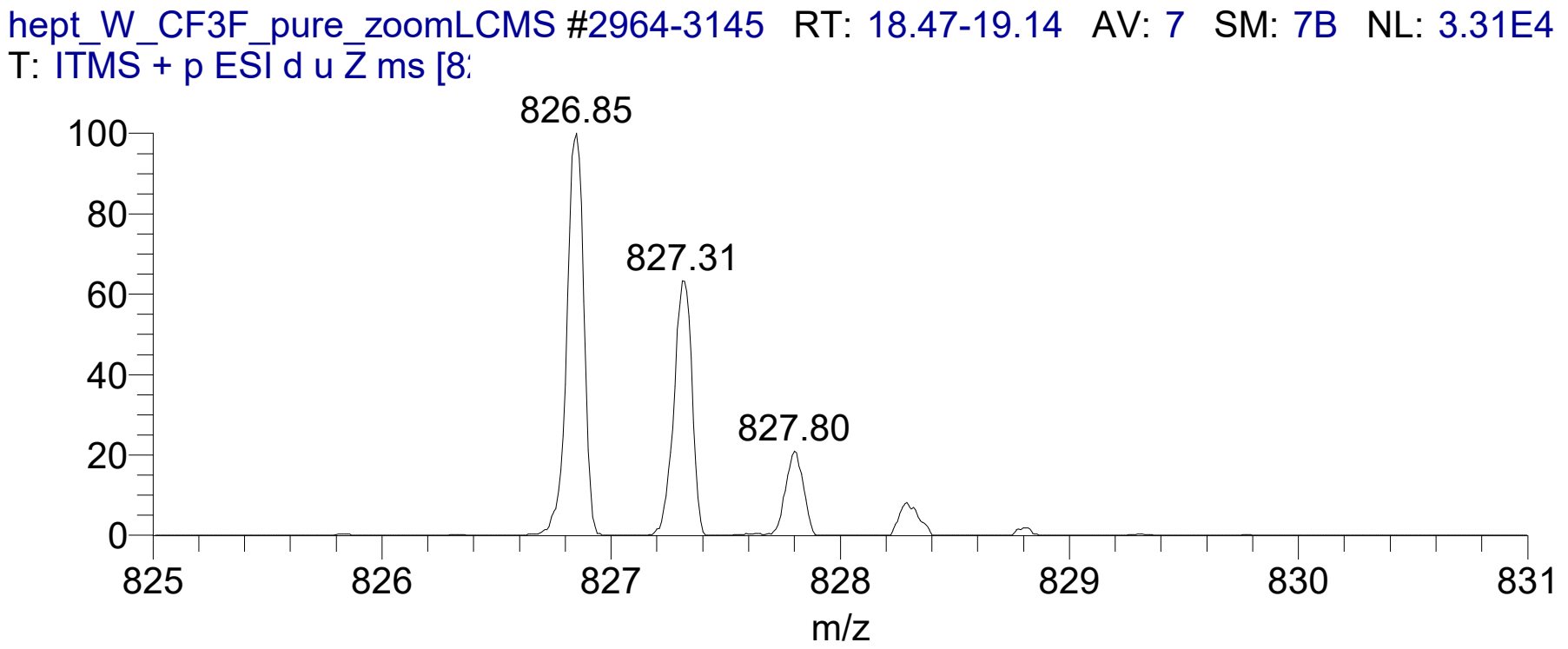

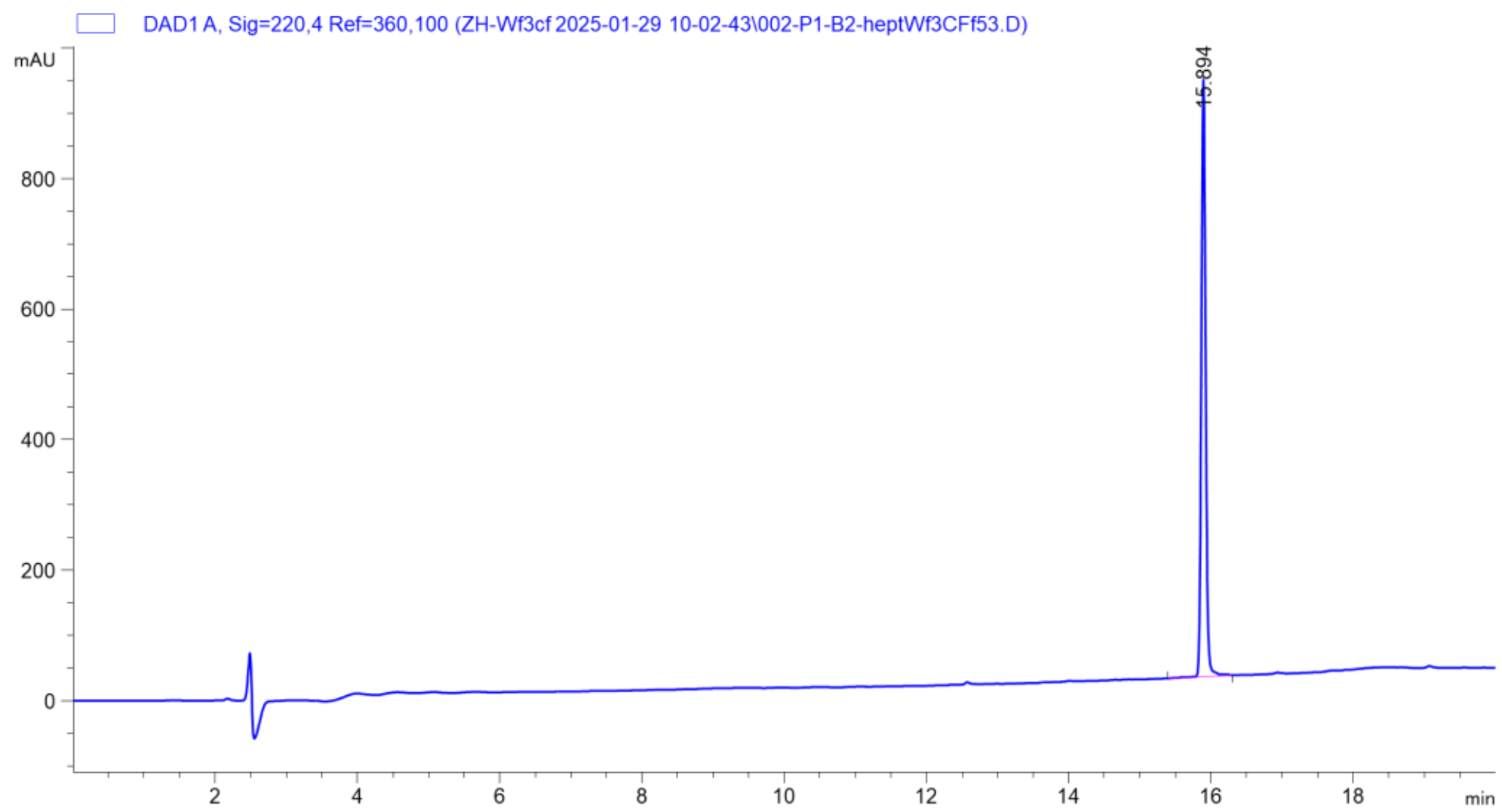

WLL-WWL dimer

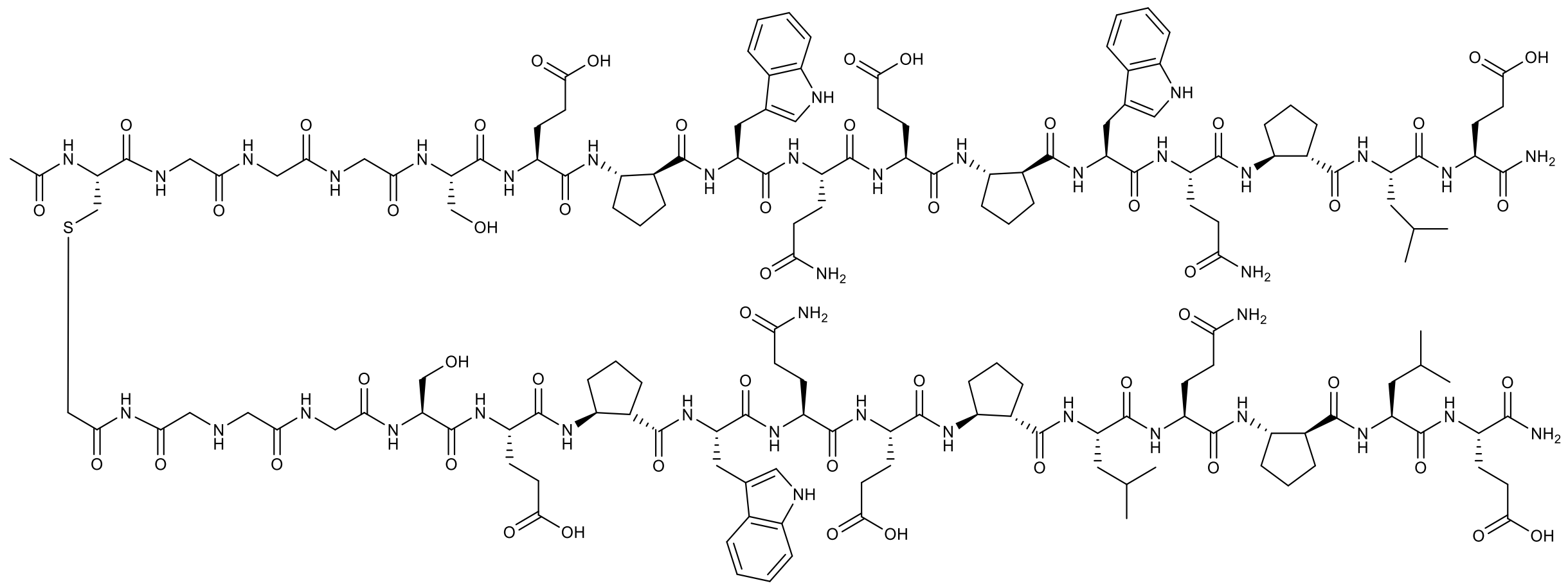

Exact Mass: 3585.65; Molecular Weight: 3587.93

$[M+2H]^{2+}$: 1794.64. $[M+3H]^{3+}$: 1196.98

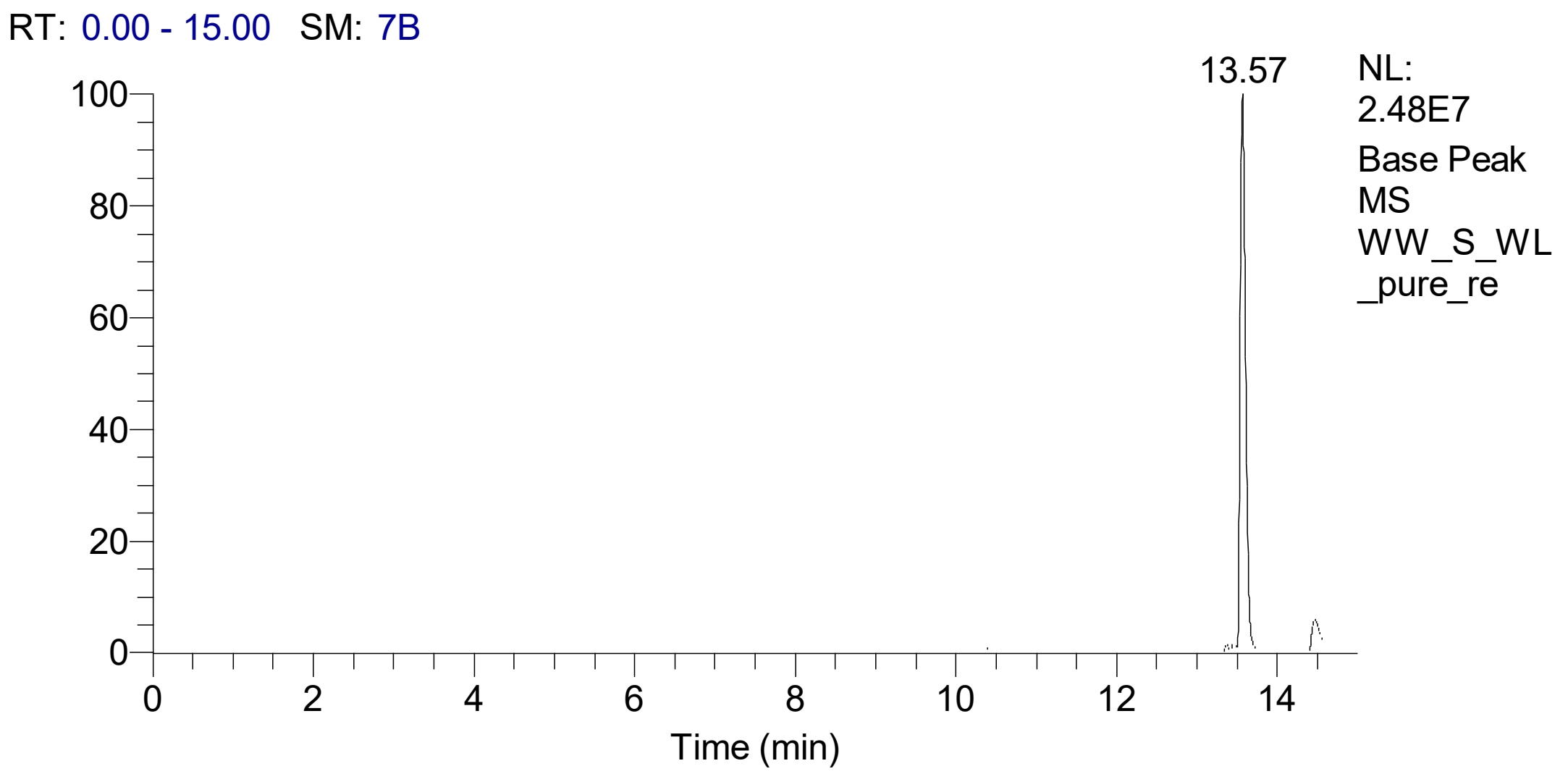

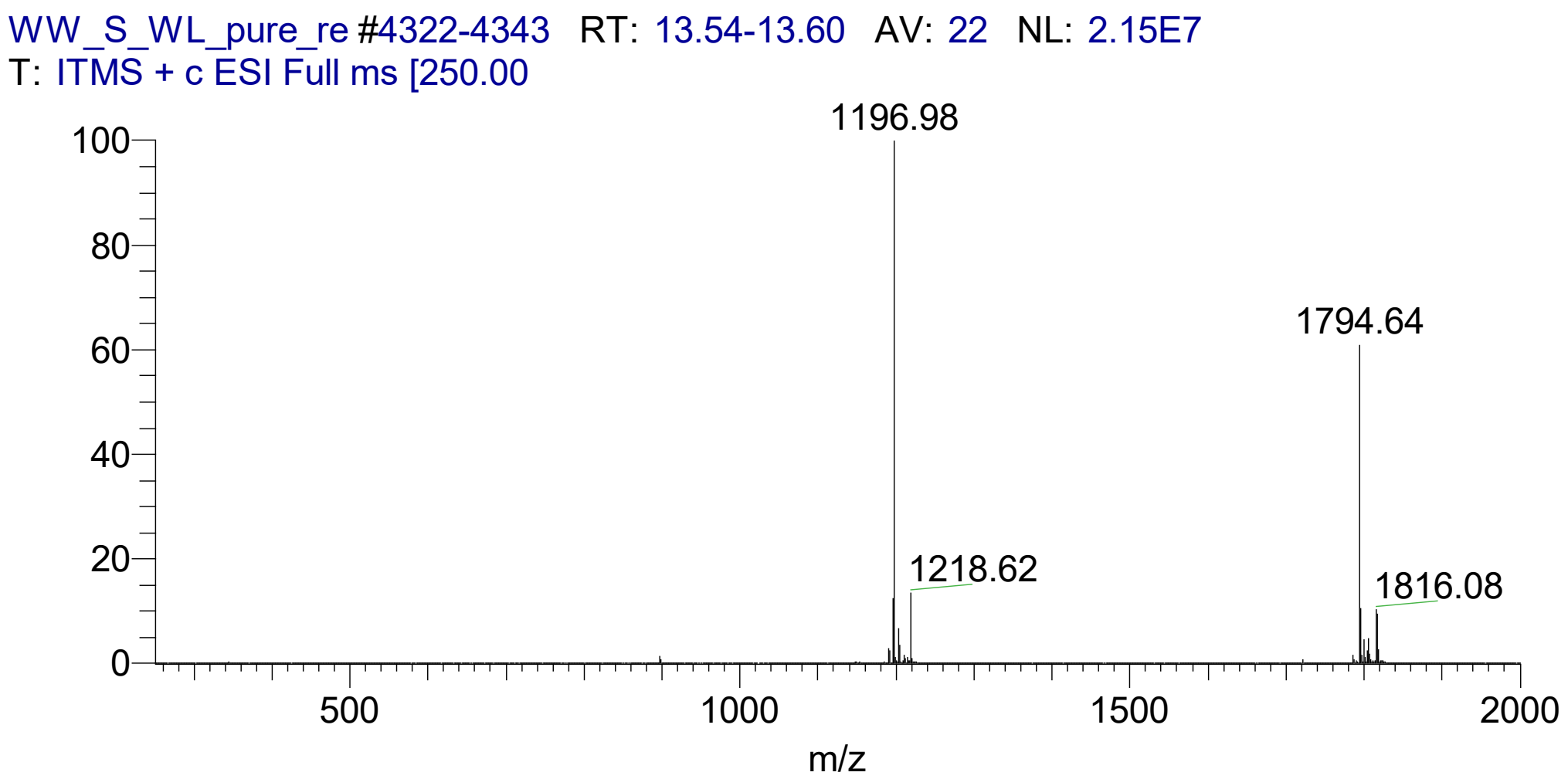

W(F$_3$CF)$_2$-WLL dimer

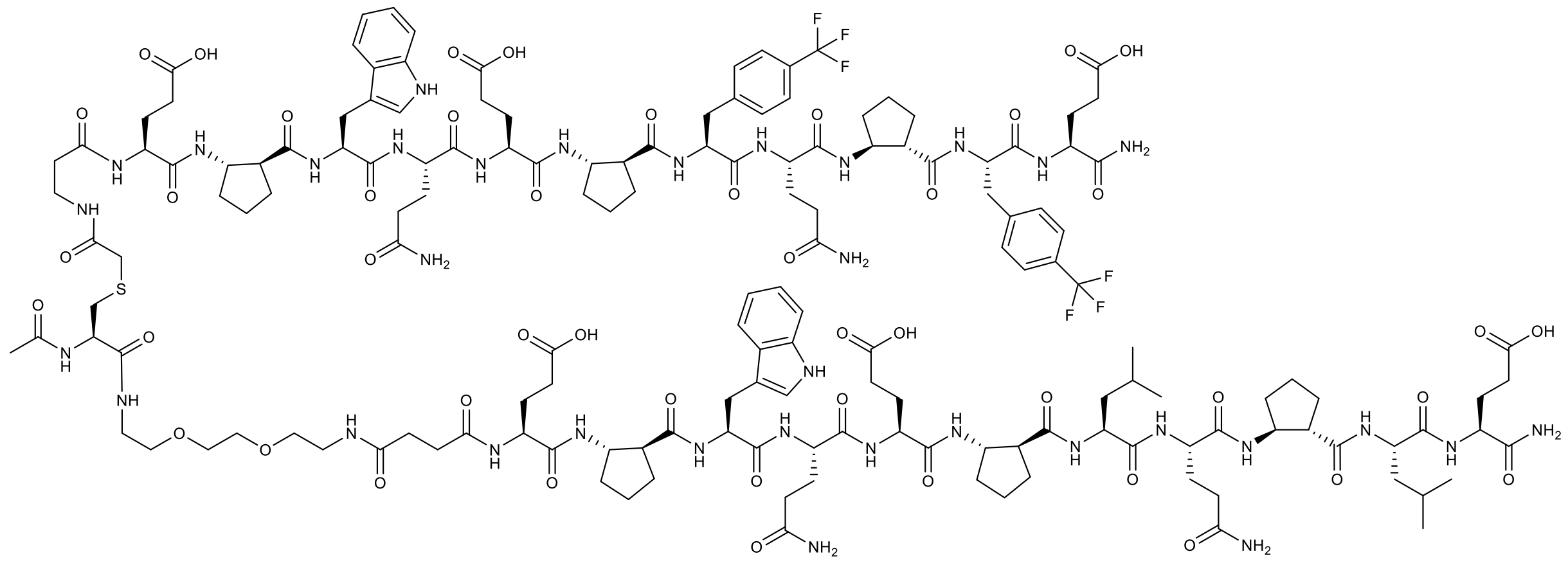

Exact Mass: 3501.570

Molecular Weight: 3503.778

$[M+2H]^{2+}$: 1753.63. $[M+3H]^{3+}$: 1169.53

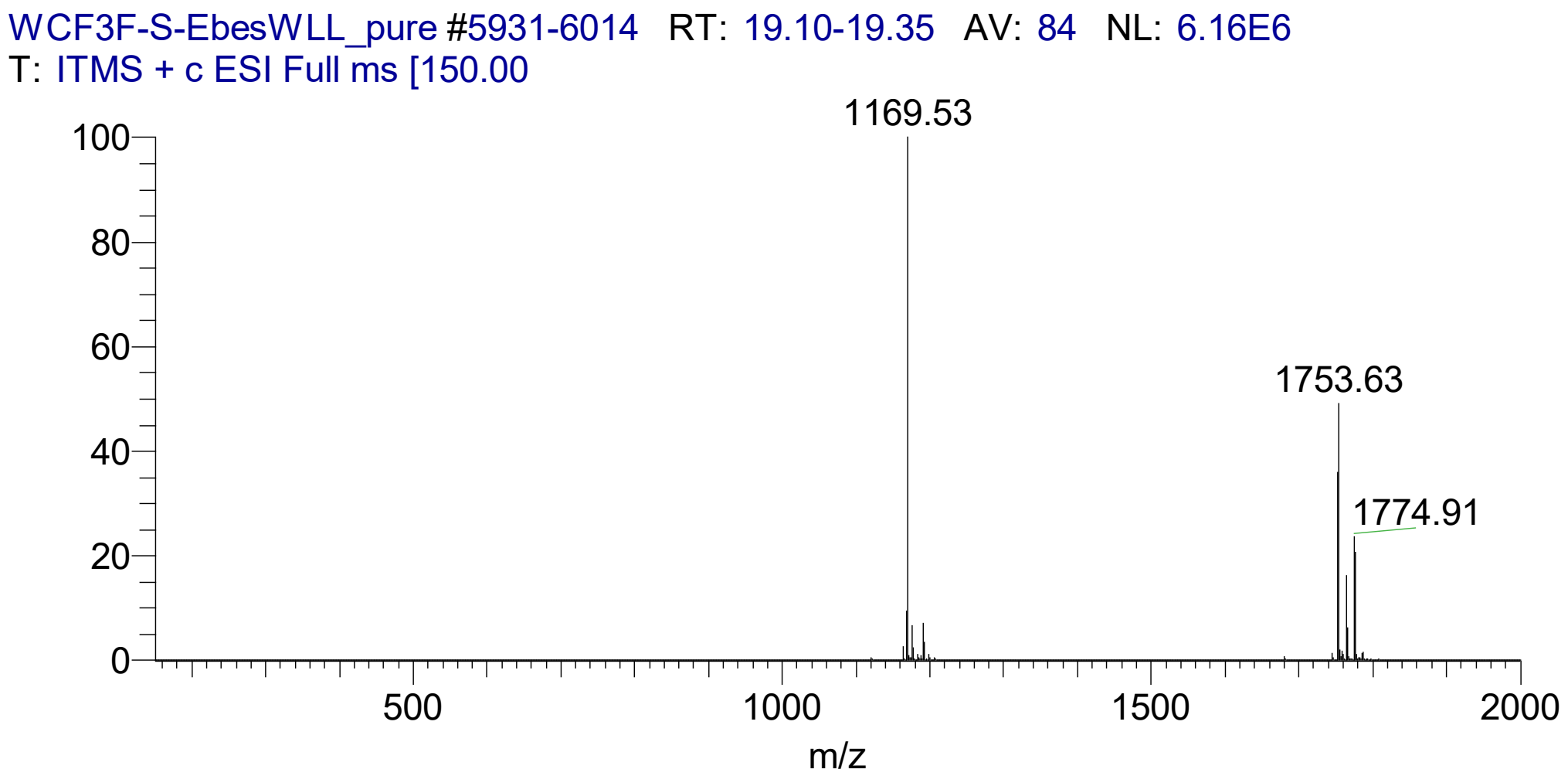

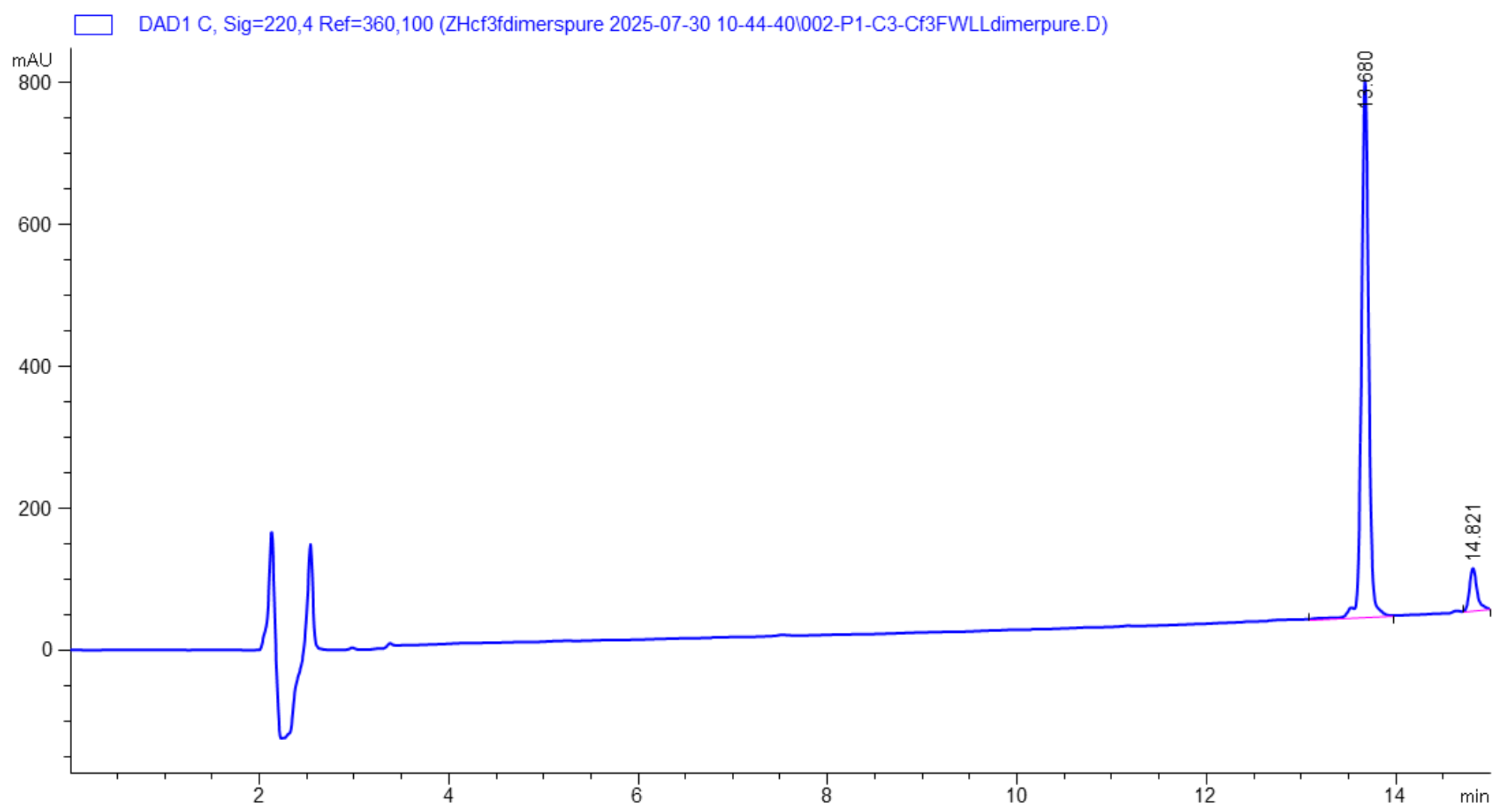

5 References

(1) Wood, C. W.; Ibarra, A. A.; Bartlett, G. J.; Wilson, A. J.; Woolfson, D. N.; Sessions, R. B. BAlaS: Fast, Interactive and Accessible Computational Alanine-Scanning Using BudeAlaScan. *Bioinformatics* **2020**, *36* (9), 2917–2919. https://doi.org/10.1093/BIOINFORMATICS/BTAA026.

(2) Marty, M. T.; Baldwin, A. J.; Marklund, E. G.; Hochberg, G. K. A.; Benesch, J. L. P.; Robinson, C. V. Bayesian Deconvolution of Mass and Ion Mobility Spectra: From Binary Interactions to Polydisperse Ensembles. *Anal. Chem.* **2015**, *87* (8), 4370–4376. https://doi.org/10.1021/acs.analchem.5b00140.

(3) Abdelkader, E. H.; Otting, G. NT*-HRV3CP: An Optimized Construct of Human Rhinovirus 14 3C Protease for High-Yield Expression and Fast Affinity-Tag Cleavage. *J Biotechnol* **2021**, *325*, 145–151. https://doi.org/10.1016/j.jbiotec.2020.11.005.

(4) Hobor, F.; Hegedüs, Z.; Ibarra, A. A.; Petrovicz, V.; Bartlett, G.; Sessions, R. B.; Wilson, A.; Edwards, T. A. Understanding P300-Transcription Factor Interactions Using Sequence Variation and Hybridization. *RSC Chemical Biology* **2022**, *3*, 546–550. https://doi.org/10.1039/d2cb00026a.

(5) Keller, S.; Vargas, C.; Zhao, H.; Piszczek, G.; Brautigam, C. A.; Schuck, P. High-Precision Isothermal Titration Calorimetry with Automated Peak-Shape Analysis. *Analytical Chemistry* **2012**, *84* (11), 5066–5073. https://doi.org/10.1021/AC3007522.

(6) Zhao, H.; Piszczek, G.; Schuck, P. SEDPHAT – A Platform for Global ITC Analysis and Global Multi-Method Analysis of Molecular Interactions. *Methods* **2015**, *76*, 137–148. https://doi.org/10.1016/J.YMETH.2014.11.012.

(7) Brautigam, C. A. Calculations and Publication-Quality Illustrations for Analytical Ultracentrifugation Data. *Methods in Enzymology* **2015**, *562*, 109–133. https://doi.org/10.1016/BS.MIE.2015.05.001.

(8) Lee, W.; Tonelli, M.; Markley, J. L. NMRFAM-SPARKY: Enhanced Software for Biomolecular NMR Spectroscopy. *Bioinformatics* **2015**, *31* (8), 1325–1327. https://doi.org/10.1093/bioinformatics/btu830.
